# Synthesis, In Silico and Kinetics Evaluation of *N*-(β-d-glucopyranosyl)-2-arylimidazole-4(5)-carboxamides and *N*-(β-d-glucopyranosyl)-4(5)-arylimidazole-2-carboxamides as Glycogen Phosphorylase Inhibitors

**DOI:** 10.3390/ijms25094591

**Published:** 2024-04-23

**Authors:** Levente Homolya, Rachel T. Mathomes, Luca Varga, Tibor Docsa, László Juhász, Joseph M. Hayes, László Somsák

**Affiliations:** 1 Department of Organic Chemistry, University of Debrecen, POB 400, H-4002 Debrecen, Hungary; levente.homolya@gmail.com (L.H.); somsak.laszlo@science.unideb.hu (L.S.); 2 School of Pharmacy & Biomedical Sciences, University of Central Lancashire, Preston PR1 2HE, UK; 3 Department of Medical Chemistry, Faculty of Medicine, University of Debrecen, Egyetem tér 1, H-4032 Debrecen, Hungary; varga.luca@med.unideb.hu (L.V.); tdocsa@med.unideb.hu (T.D.)

**Keywords:** glucose analogues, glycogen phosphorylase inhibitor, tautomers, type 2 diabetes

## Abstract

Recently studied *N*-(β-d-glucopyranosyl)-3-aryl-1,2,4-triazole-5-carboxamides have proven to be low micromolar inhibitors of glycogen phosphorylase (GP), a validated target for the treatment of type 2 *diabetes mellitus*. Since in other settings, the bioisosteric replacement of the 1,2,4-triazole moiety with imidazole resulted in significantly more efficient GP inhibitors, in silico calculations using Glide molecular docking along with unbound state DFT calculations were performed on *N*-(β-d-glucopyranosyl)-arylimidazole-carboxamides, revealing their potential for strong GP inhibition. The syntheses of the target compounds involved the formation of an amide bond between per-*O*-acetylated β-d-glucopyranosylamine and the corresponding arylimidazole-carboxylic acids. Kinetics experiments on rabbit muscle GP*b* revealed low micromolar inhibitors, with the best inhibition constants (*K*_i_s) of ~3–4 µM obtained for 1- and 2-naphthyl-substituted *N*-(β-d-glucopyranosyl)-imidazolecarboxamides, **2b**–**c**. The predicted protein–ligand interactions responsible for the observed potencies are discussed and will facilitate the structure-based design of other inhibitors targeting this important therapeutic target. Meanwhile, the importance of the careful consideration of ligand tautomeric states in binding calculations is highlighted, with the usefulness of DFT calculations in this regard proposed.

## 1. Introduction

*Diabetes mellitus* is a growing pandemic which poses many challenges for global health and economics, of which type 2 *diabetes mellitus* (T2DM) is the most common. The global prevalence of T2DM is projected to increase to 7079 people per 100,000 by 2030 and reflects a continuing trend across all the world [1]. It is important to also note that there has been a concerning rise in its prevalence in lower-income countries. T2DM is documented as a metabolic disorder, attributed to abnormal insulin production and/or peripheral resistance to the action of insulin, advancing the development of hyperglycaemia [2]. While there are different classes of approved antihyperglycaemic drugs [3], these treatments do not achieve the required degree of glucose control for a large number of patients [4]. Several complications may arise in individuals suffering from T2DM when their blood glucose levels are inadequately regulated, including cardiovascular disease, loss of vision, neuropathy and nephropathy [5]. Overall, there is an urgent need for new and more effective therapeutic options, combined with clinical preventive measures.

The glycogenolysis pathway has a direct impact on blood glucose levels; as glycogen phosphorylase (GP; EC 2.4.1.1) plays a fundamental role in this pathway, it is therefore an attractive target for the treatment of T2DM. GP exists in three isoforms: the liver, muscle and brain. More specifically, the liver isoform is of interest as it catalyses the breakdown of glycogen into glucose in the liver, the inhibition of which has potential to reduce hyperglycaemia in T2DM patients [6]. Additionally, GP is also considered of promise as a target for other conditions such as myocardial and cerebral ischemias [7,8] and different cancers such as glioblastoma [9,10,11]. Seven different GP ligand-binding sites have been discovered to date: catalytic, allosteric, new allosteric, glycogen storage, inhibitor, benzimidazole and quercetin-binding sites, with the catalytic site being a focal point of research to date [12,13,14,15]. Physiological inhibitors of GP’s catalytic site are typically glucose analogues such as α-d-glucose, with an inhibition constant (*K*_i_) of 1.7 mM, and its anomer, β-d-glucose, which binds with a *K_i_* of 7.4 mM [16,17]. The most potent glucose analogue inhibitors of GP are based on β-d-glucose and possess a carefully designed linker group attached to the β-position of the anomeric C-1 atom of the glucose ring and an extension of the linker, usually with a planar aromatic group, exploiting the favourable interactions in the so-called β-cavity of the GP catalytic site [12,18,19]. The most efficient nanomolar inhibitors are exemplified in Table 1, representing the best three scaffolds to date: *N*-(β-d-glucopyranosyl)-*N*’-aroyl ureas (e.g., **I**) [15], glucopyranosylidene-spiro-heterocycles (e.g., **II**, **III**) [20] and *C*-β-d-glucopyranosyl heterocycles (e.g., **IV**, **V**) [21]. A comparison of the inhibitory efficiency of **IV** and **V** shows the beneficial effect of switching to the imidazole **V** from the 1,2,4-triazole **IV**.

Non-classical bioisosteric replacements [22,23] of the highlighted amide moiety in **I** with several oxadiazole-type heterocycles were previously studied (Table 1, **VI**–**VIII**), which, however, resulted in no better inhibitors than **I**. Meanwhile, in silico docking calculations have been performed on *N*-(β-d-glucopyranosyl)-3-substituted-1,2,4-triazole-5-carboxamide analogues **IX** with aryl substitutions: phenyl (**a**), 1-naphtyhl (**b**) and 2-naphthyl (**c**) [24]. The phenyl-substituted **IXa** was reported as the most potent of the three analogues acting at the catalytic site, with a *K*_i_ of 1 µM for rabbit muscle glycogen phosphorylase *b* (rmGP*b*).

**Table 1:** Selected glucose-derived inhibitors of glycogen phosphorylase (*K*_i_ [µM]) ^a^ and the target compounds of this study.

Submicromolar Inhibitors with Diverse Scaffolds				
** 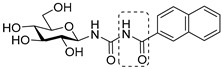 **	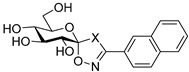		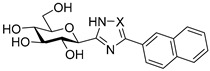	
**I** (0.35) [15]	**II** X = S (0.16) [25]		**IV** X = N (0.41) [21]	
	**III** X = CH_2_ (0.63) [20]		**V** X = CH (0.031) [21]	
Bioisosteric replacements in **I** and compounds studied				
	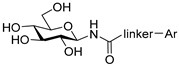			
Linker	Ar			
	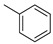	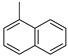		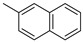
	**a**	**b**		**c**
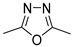	545 ^b^	172 ^b^		30
**VI** [26]				
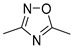	136 ^b^	33		no inh.
**VII [26]**				
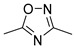	104	145 ^b^		no inh.
**VIII [26]**				
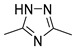	1	not studied kinetically		9.2
**IX [24]**				
			
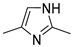	Target compounds in this study			
**1**				
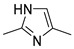				
**2**				

^a^ Inhibition data refer to rabbit muscle glycogen phosphorylase *b* (rmGP*b*). ^b^ Calculated from the IC_50_ values using the Cheng–Prusoff equation: *K*_i_ = IC_50_/(1 + [S]/*K*_m_).

As a continuation of the bioisosteric replacement studies in **I**, and based on the experiences with **IV** and **V**, computational, synthetic and enzyme kinetic investigation of imidazole-containing compounds was undertaken in this work (Table 1, target compounds **1** and **2**). Docking predictions for the bound states and DFT calculations on the unbound states revealed potential for potent GP inhibition, consistent with that observed previously for the *N*-(β-d-glucopyranosyl)-3-substituted-1,2,4-triazole-5-carboxamides analogues. Synthesis and subsequent kinetic evaluation of GP inhibition revealed this to be the case.

## 2. Results and Discussion

### 2.1. Computational

#### 2.1.1. Unbound State Calculations

For the ligands, using Ar = phenyl as a prototype for the calculations, we first computed the preferred unbound state ionisation/tautomer states and the conformations of the prototype target ligands **1a** and **2a**, which would have a considerable impact on the binding potential for GP inhibition [18,24,27]. The potential for the protonation of the ligands was determined by using DFT and Jaguar *pK*_a_ calculations, revealing that the ligands would be in a neutral charge state. 

**Figure 1:**
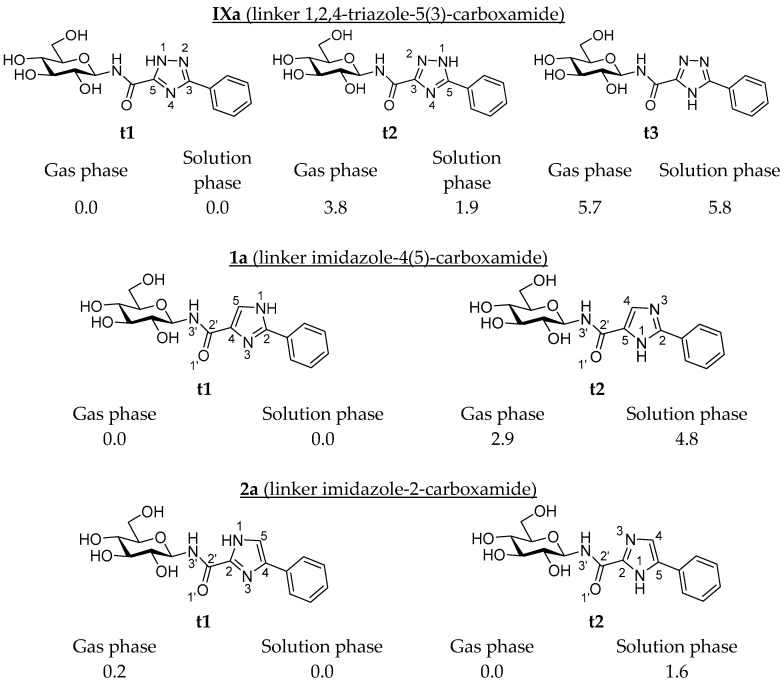
A comparison of the various tautomeric forms of prototype benchmark **IXa** and prototype target **1a** and **2a** ligands with their predicted relative energies (kcal mol^−1^) calculated using DFT. Gas-phase energies were from M06-2X/6-31+G* optimisations, with solution-phase energies (M06-2X/6-31+G* and SM8 model of water solvation) calculated as single-point energies at the gas-phase optimised geometries.

The predicted *pK*_a_s for the protonated +1-charged compound **1a** considering the deprotonation of N2(H) and N4(H) were 4.03 and 2.96, respectively, while for compound **2a**, the corresponding values for N1(H) and N4(H) were 2.21 and 1.95, respectively. Considering the unbound tautomeric states of **1a** and **2a** (with the previously studied **IXa** also calculated for comparison), Monte Carlo Multiple Minima (MCMM) conformational search calculations were performed and post-processed using DFT (M06-2X/6-31+G*) gas- and solution-phase (SM8) energy calculations. Previous successful applications of this approach to deciphering the tautomeric state preferences of β-d-glucose-heterocyclic-type compounds and their influence on binding have been reported [18,27]. The results of these calculations are shown in Figure 1. For the **IXa** and **1a** ligands, the gas- and solution-phase-favoured tautomers were consistently **t1.** For ligand **IXa**, **t1** was predicted to be more favourable in solution by 1.9 kcal/mol, and for ligand **1a**, **t1** was more strongly favoured, by 4.8 kcal/mol. In the case of **2a**, the **t1** and **t2** tautomers were similar in their gas-phase energies; however, the **t1** tautomeric state was more favourable by 1.6 kcal/mol in solution. Solution-phase energies assume priority in terms of the favoured unbound states, and hence **t1** was favoured for all three of the prototype ligands **IXa**, **1a** and **2a**. A study of the tautomeric preferences of the ligands in the solved PDB complexes revealed that the most stable water solution-phase tautomer was predominantly favoured for binding, depending on the free energy difference between the relevant tautomers [28]. 

#### 2.1.2. Bound State Calculations

For the bound states, all possible tautomeric states (Table 2) were considered in terms of the binding to GP, with the docking calculations performed using Glide in standard precision (SP) mode. The Jaguar *pK*_a_s of the protonated (+1-charged) target prototype ligands **1a** (*pK_a_*s~3–4) and **2a** (*pK*_a_s~2) were quite low, as mentioned above. Nevertheless, within the core of a protein matrix, the pH may substantially differ compared to in solvent alone [29]. With this in mind, an initial test of the prototype ligands **1a** and **2a** docked in their protonated state revealed that they did not outscore the neutral tautomers. Only the neutral tautomers, therefore, were considered from hereon. The results of the docking predictions in terms of their Glidescores and docking scores are shown in Table 2. Docking scores incorporate the approximate tautomeric state penalties into their final scores (an adjusted Glidescore) based on the LigPrep (unbound state)-predicted tautomeric state preferences. However, the unbound tautomeric state Monte Carlo/DFT calculations (alternative method) assume greater accuracy and priority in the analysis, as outlined below.

As mentioned, ligands **IXa**–**c** were used as a benchmark for comparison. For **IXa**–**c**, while the Glidescores were relatively similar for the three tautomers **t1**–**t3**, a preference for **t1** binding was clearly predicted by the docking scores, which were in the range of −9.42–−10.71 for the three aromatic substituents. This is consistent with our DFT-calculated preference for **t1** in the unbound state.

**Figure 2:**
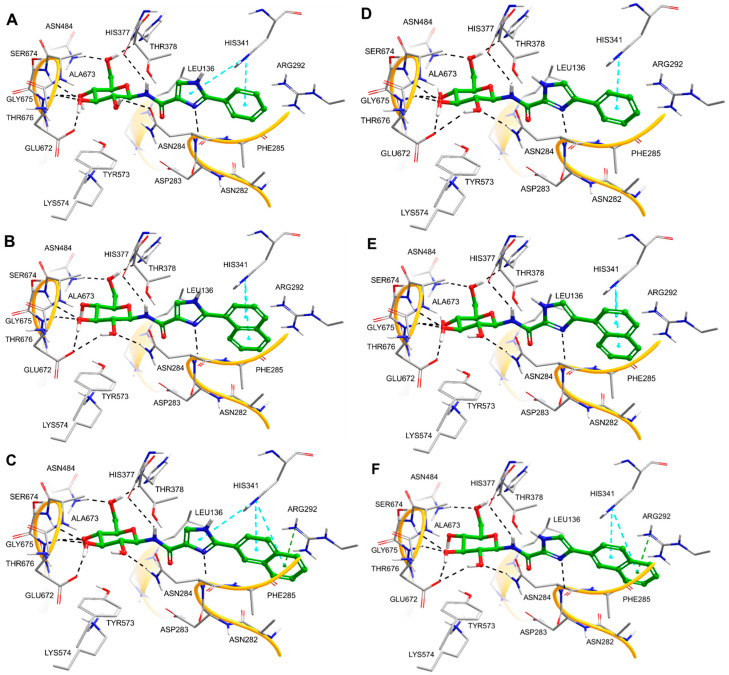
Predicted GP-binding interactions of target ligands **1a**–**c** in their respective panels (**A**–**C**) and **2a**–**c** shown in their respective panels (**D**–**F**). Binding poses were based on Glide SP docking calculations, as described in the text. Hydrogen bonds are shown as black dash lines; π-π interactions as dashed cyan lines; cation-π interactions as dashed green lines.

For **1a**–**c**, the docking scores (and Glidescores) were similar to those of ligands **IXa**–**c**. However, the docking scores for **1a**–**c** were comparable for the tautomers **t1** (−9.17–−10.33) and **t2** (−9.30–−10.72), as were the Glidescores. Given that the **t1** tautomer was most favourable in the solution phase by 4.8 kcal/mol, it can be proposed that the compound binds as **t1**. The binding of the three analogues **1a**–**c** is shown in Figure 2A–C, where we can see that the ligands exploit similar interactions at the GP catalytic site, with the predicted binding poses close to superimposable, apart from the differing aromatic groups. The standard glucose moiety hydrogen bonds involving the hydroxyl groups with the Asn284, His377, Asn484, Glu672 and Gly675 residues were expected and observed. The 2-substituted-imidazole-4(5)-carboxamide is involved in important hydrogen bonding interactions from N(3’)H with the His377 backbone’s CO (interactions with this group are recognised as crucial to the good inhibitory potential of glucose analogues [12,15]) and close to (~3.2 Å) the hydrogen bonding distance of the ligand O(1’) with the Leu136 backbone’s NH. The Asn284 backbone’s NH is able to exploit a favourable interaction (~2.6 Å) with imidazole N(3). Protein–ligand interaction analysis using the Maestro program [30] reported π-π interactions between the ligand (**1a** and **1c**) imidazoles and the His341 side-chain. For the inhibitor aromatic groups, there were also π-π interactions for all three analogues **1a**–**c** with His341, but cation-π interactions with the +1-charged Arg292 side-chain were best positioned for the bound **1c** (2-naphthyl) analogue.

For ligands **2a**–**c**, the docking scores and Glidescores were again quite similar to those of **IXa**–**c**. Considering the two tautomers of **2a**–**c**, the docking scores were again similar for **t1** (−9.29–−10.59) and **t2** (−9.18–−10.55), as were the Glidescores. From the Monte Carlo/DFT unbound state calculations, **t1** was the most stable unbound state tautomer in solution by 1.6 kcal/mol, and it can be proposed that the ligands bind in this state. The predicted binding of **t1** for ligands **2a**–**c** is shown in Figure 2D–F, where, again, we can see that the three analogues bind in a similar manner. Indeed, the ligand poses are close to superimposable with the respective **1a**–**c** ligands (i.e., including the aromatic groups). The protein–ligand interactions include the aforementioned standard glucose moiety hydroxyl interactions at the binding site, described above for **1a**–**c**. The 4(5)-substituted-imidazole-2-carboxamide is involved in hydrogen bond interactions with the His377 backbone’s CO through ligand N(3′)H. The O(1′) is close to hydrogen bonding distance from the NH in Leu136 (~3.1 Å), as also observed for **1a**–**c**. However, for **2a**–**c**, the imidazole N(1)H also is directed towards the His377 backbone’s CO, although the distances range between 3.3 and 3.6 Å in the predicted models. As with **1a**–**c**, the ligand aromatic groups are predicted to form π-π interactions with the His341 sidechain, and in the case of **1c**, cation-π interactions involving the Arg292 side-chain with the 2-naphthyl ring.

Based on the predictions, therefore, it was concluded that the imidazole-4(5)-carboxamide (**1**) and imidazole-2-carboxamide (**2**)-type linkers may reveal ligands with at least a similar GP inhibitory potential to the previously reported 1,2,4-triazole-5-carboxamide analogues [24], so compounds **1a**–**c** and **2a**–**c** were considered for synthesis.

### 2.2. Synthesis

**Scheme 1:**
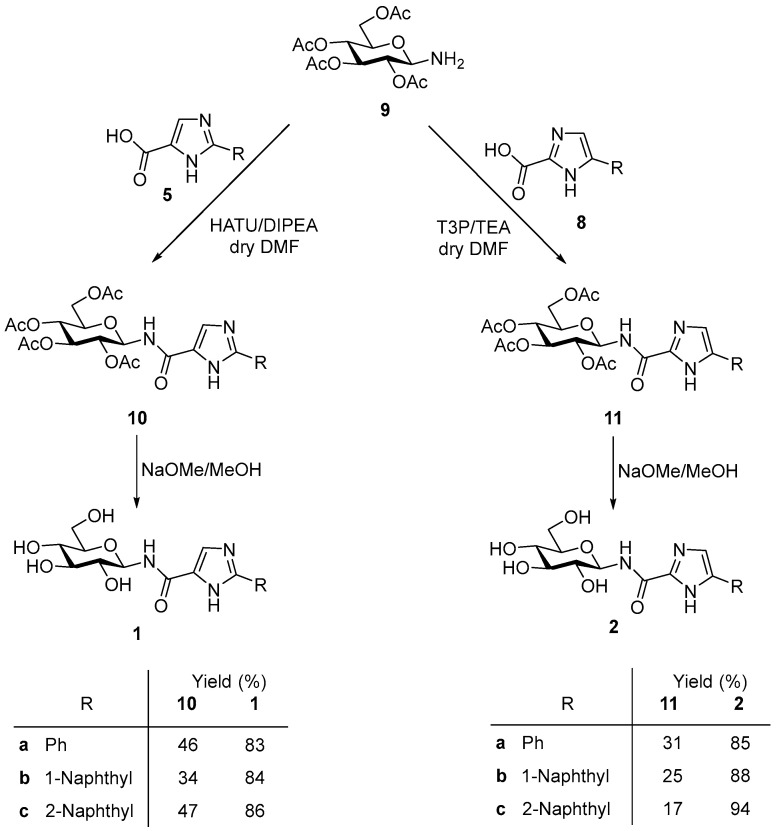
Preparation of the target *N*-(β-d-glucopyranosyl) imidazolecarboxamides **1** and **2**.

The syntheses of the target *N*-(β-d-glucopyranosyl)-arylimidazole-carboxamides (**1a**–**c** and **2a**–**c**) were based on the formation of an amide bond between 2,3,4,6-tetra-*O*-acetyl-β-d-glucopyranosylamine (**9**) and the corresponding arylimidazole-carboxylic acids **5a**–**c** and **8a**–**c** (Scheme 1). Our work started with the synthesis of the desired arylimidazole-carboxylic acids **5a**–**c** and **8a**–**c** (Table 3 and Table 4). A Suzuki reaction was performed from **3** [31] using arylboronic acids to give ethyl 2-arylimidazole-4(5)-carboxylates **4a**–**c** in low and moderate yields (Table 3). Based on the work of Thurkauf and co-workers [32], the ethyl 4(5)-arylimidazole-2-carboxylates 7**a**–**c** were gained from the ring closure of the corresponding 2-amino-1-arylethan-1-one [33] derivatives, with **6** previously treated with triethyloxonium tetrafluoroborate (Table 4). Hydrolysis of esters **4a**–**c** and **7a**–**c** under alkaline conditions gave the carboxylic acids **5a**–**c** and **8a**–**c** in good yields. Phenyl derivatives of the imidazole carboxylic acids **5a** [34] and **8a** [35] and the ethyl ester derivatives **4a** [36] and **7a** [32] thereof have been reported previously in the literature.

Amide bond formation was carried out under different conditions for the synthesis of the 2-arylimidazole-4(5)-carboxamides **1a**–**c** and 4(5)-arylimidazole-2-carboxamides **2a**–**c**. HATU [37] for the former ones and T3P [38] for the latter ones were used as coupling agents to give the desired compounds **10a**–**c** and **11a**–**c** in moderate and good yields. Removal of the *O*-acetyl protecting groups from **10a**–**c** and **11a**–**c** under Zemplén conditions gave the target molecules **1a**–**c** and **2a**–**c** in good and excellent yields. The structure and the purity of the intermediates **4a**–**c**, **5a**–**c**, **7a**–**c, 8a**–**c**, **10a**–**c** and **11a**–**c** and the target compounds **1a**–**c** and **2a**–**c** were determined using 1D- and 2D-NMR experiments and HRMS measurements. The sugar ring conformations, the anomeric configurations and the final formation of the amide bonds followed from the above spectra and require no special comments. The NMR spectra of these derivatives clearly showed the presence of a tautomeric mixture of imidazole derivatives (see Supplementary Information). The duplication of the signals in the ^1^H- and ^13^C-NMR spectra indicates the presence of the tautomeric 2,4- and 2,5-disubstituted-1*H*-imidazoles.

### 2.3. Kinetics

Inhibitory constants (*K*_i_) were determined for rabbit muscle glycogen phosphorylase. Enzyme activity was assayed in the direction of glycogen synthesis, as previously published [39]. The experiments were performed using the rabbit muscle phosphorylase *b* (dephosphorylated, GP*b*) isoform. Kinetic data for the inhibition of GP*b* by the compounds were obtained in the presence of 10 μg/mL of the enzyme, varying the concentrations of α-d-glucose-1-phosphate (4–40 mM) and using constant concentrations (1%) of glycogen and AMP (1 mM). The enzymatic activities were presented in the form of a double reciprocal plot (Lineweaver–Burk). The plots were analysed using a non-linear data analysis program. The inhibitor constants (*K_i_*) were determined using secondary plots, replotting the slopes from the Lineweaver–Burk plot against the inhibitor concentrations. The means of the standard errors for all calculated kinetic parameters averaged to less than 10% [40].

The results of the kinetics experiments for the target and synthesised compounds **1a**–**c** and **2a**–**c** are shown in Table 5. All the ligands were revealed as low micromolar potent inhibitors of rmGP*b* (≤16 µM), but with **2a** being slightly less potent (*K*_i_ = 67.4 µM).

The better potency of the **2b**–**c** ligands (*K*_i_s~3–4 µM) compared to their **1b**–**c** (*K*_i_s~13–16 µM) counterparts is likely due to the potential of additional interactions from imidazole N(1)H with the His377 backbone’s CO (predicted as slightly longer than hydrogen bonding, Figure 2), in combination with those from carboxamide N(3’)H. It is unclear, however, regarding the degree of the **2a** ligands’ lesser potency, except that it is unable to exploit the same interactions as the more extended 1- and 2-naphthyl groups in the GP catalytic site β-cavity. This, however, was not the case for **1a** (*K*_i_ = 10.4 µM). The previously reported benchmark ligand **IXa** had a *K*_i_ of 1 µM in comparison; however, both **1c** (*K*_i_ = 12.8 µM) and **2c** (*K*_i_ = 3.3 µM) were similar or more potent, respectively, compared to the related **IXc** (*K*_i_ = 9.2 µM) compound.

## 3. Conclusions

Glycogen phosphorylase is an important therapeutic target for predominant conditions such as T2D and cancer. The design of effective glucose analogue inhibitors targeting the catalytic site is strongly dependent on the structure of the linker group, linking the glucose moiety with different (mainly aromatic) groups that extend into the catalytic site’s β-cavity. In this study, we investigated the potential of ligands **1** and **2** with imidazole-4(5)-carboxamide (**1**) and imidazole-2-carboxamide (**2**)-type linkers in terms of their GP inhibitory potential, as a follow-up study to the previously reported 1,2,4-triazole-5-carboxamide (**IX**) linkers [24]. Monte Carlo and DFT calculations on the unbound states of the ligands considering the different tautomers and Glide docking calculations for the prediction of the protein–ligand binding modes to GP predicted that both linkers may have at least similar effectiveness to the 1,2,4-triazole-5-carboxamide (**IX**), and ligands **1a**–**c** and **2a**–**c** with phenyl, 1-naphthyl and 2-naphthyl groups were considered for subsequent synthesis. The key step in the syntheses of the target compounds **1a**–**c** and **2a**–**c** was the formation of an amide bond between per-*O*-acetylated β-d-glucopyranosylamine and the corresponding arylimidazole-carboxylic acids. Enzyme activity was assayed in the direction of glycogen synthesis using the rabbit muscle glycogen phosphorylase *b* (dephosphorylated, GP*b*) isoform. These kinetic data revealed that all the synthesised compounds are low micromolar inhibitors of rmGP*b* (≤16 µM), but with **2a** being slightly less potent. Novel potent inhibitors of GP have been reported which can be considered in cellular models of disease, as well as facilitating the structure-based design of new glucose analogue GP inhibitors. For example, their effects on glycogenolysis at the cellular level could next be probed [18]; also, the effectiveness of glucose analogue GP inhibitors against glioblastoma would be of interest [11]. The importance of consideration of the tautomeric preferences of ligands in protein–ligand binding studies has again been highlighted in this study, as well as the usefulness of DFT calculations for this purpose [27].

## 4. Computational Details

### 4.1. Protein Preparation

Using the co-crystallised complex of rmGP*b* with a 3-(β-d-glucopyranosyl)-5-substituted-1,2,4-triazole fluorene derivative (PDB ID: 6F3R) [41], Schrodinger’s ‘Protein Preparation Wizard’ [30] was employed to prepare the GP*b* receptor structure for the calculations. Water molecules within 5 Å of the cognate ligand were initially retained (deleted for subsequent docking), missing hydrogen atoms added, bond orders assigned and the tautomer/ionisation states of the protein residues established according to *pK*_a_ predictions using the PROPKA program [42] at a pH of 7. Minimisation was performed using the OPLS3e forcefield [43] to alleviate steric clashes and restrain heavy atoms to root mean square deviation (RMSD) within 0.3 Å of their crystallographic locations.

### 4.2. Ligand Preparation

All the ligands (**IXa**–**c**, **1a**–**c** and **2a**–**c**) were prepared for calculations using Schrodinger’s Maestro and the LigPrep 5.6 program with Epik. [30] Favourable minimised tautomeric/ionisation states were produced for each ligand with a target pH of 7 ± 2. The 1,2,4-triazole (**IX**) linker produced three tautomeric forms; the imidazole ligands **1** and **2** both produced two tautomers, as shown in Figure 1. To check the potential for protonation (+1 charge) of the imidazole heterocycles, Jaguar *pK*_a_ calculations [30] were performed on **1a** (*pK*_a_~3–4) and **2a** (*pK*_a_~2), indicating these ligand states are not favoured in the free state and are unlikely in the bound state.

To calculate the unbound state tautomeric preferences, Monte Carlo Multiple Minima (MCMM) conformational searches using MacroModel 13.0 [30] were initially performed on the unbound prototype **IXa**, **1a** and **2a** ligands in their different tautomeric states. A total of 20,000 Monte Carlo steps were used, each followed by 200-step minimisation (truncated Newton conjugate gradient method); the OPLS3e forcefield was used together with an energy window of 7.5 kcal/mol above the global minimum to store the conformations. Redundant conformations were considered using an RMSD of 1.0 Å. The generated conformations were post-processed using DFT gas-phase (M06-2X/6-31 + G*) [44,45,46] optimisations, followed by solution-phase single-point energy calculations at the gas-phase optimum geometries (most stable conformations determined). For this purpose, M06-2X/6-31 + G* together with the SM8 continuum model [47] for water solvation were employed. The DFT calculations were performed using Jaguar 11.0. [30]

### 4.3. Docking Details

For the docking calculations using Glide 8.9 [30], the GP*b* catalytic binding site’s shape and properties were mapped onto grids with dimensions of 27.6 Å × 27.6 Å × 27.6 Å centred on the native co-crystallised ligand (PDB code: 6F3R). The default parameters were applied, including van der Waals radius scaling (by 0.8). Positional constraints (1.0 Å) were applied to the four glucose hydroxyl O atoms to retain the β-d-glucopyranosyl moiety in its well-established crystallographic location. The docking calculations were performed in standard precision (SP) mode, including rewards for intra-molecular hydrogen bonds, enhanced planarity for conjugated π-groups and post-docking minimisation with strain correction, with up to 10 poses per ligand saved.

## 5. Experimental

### 5.1. General Methods

The solvents were purified by distillation. Dichloromethane (DCM) was refluxed and distilled from P_4_O_10_ and stored over 4 Å molecular sieves. Methanol was refluxed with magnesium turnings and iodine for a couple of hours and was distilled. Further, 1,4-dioxane was distilled from sodium sand and stored over sodium wires. TLC was performed using DC Kieselgel 60 F_254_ (Merck, Rahway, NJ, USA) plates, developed under 254 nm UV light and/or sprayed with EtOH/cc. H_2_SO_4_/p-anisaldehyde (96:5:1) and heated to 150 °C. For column chromatography, the Kieselgel 60 (Merck, particle size 0.063–0.200 mm) was used. Optical rotations were determined using a Jasco P-2000 polarimeter at 25 °C. The NMR spectra were recorded using a Bruker Avance 400 MHz (400/100 MHz for ^1^H/^13^C) spectrometer at 298 ± 0.1 K. Chemical shifts are referenced to TMS or the residual solvent peaks. Chemical shifts are reported in ppm. All the compounds were characterised according to their one- (^1^H and ^13^C) and two-dimensional (COSY, HSQC, HMBC) NMR spectra. High-resolution mass spectra were recorded using a Bruker maXis II UHR ESI-TOF MS instrument in positive mode. The 2,3,4,6-tetra-*O*-acetyl-β-d-glucopyranosylamine (**9**) [48], 2-amino-1-arylethan-1-one hydrochlorides [33] and ethyl 2-bromo-1*H*-imidazole-4(5)-carboxylate (**3**) [31] were synthesised according to the procedures in the literature.

#### 5.1.1. General Procedure A for the Synthesis of Ethyl 2-aryl-1*H*-imidazole-4(5)-carboxylate Derivatives

Ethyl 2-bromo-1*H*-imidazole-4(5)-carboxylate (**3**) (1 equiv.), arylboronic acid (2 equiv.) and Pd(amphos)Cl_2_ (0.05 equiv.) were dissolved in dry 1,4-dioxane (2 mL/mmol as the starting materials), and then dry TEA (2 equiv.) was added to the solution. The mixture was stirred under an inert atmosphere (argon) at 140 °C for 2 h using MW irradiation. The reaction mixture was diluted with water and extracted with EtOAc. The combined organic layers were washed with water and brine, dried over MgSO_4_ and filtered, and the solvent was removed under reduced pressure. The crude product was purified using column chromatography.

#### 5.1.2. General Procedure B for the Synthesis of Ethyl 4(5)-aryl-1*H*-imidazole-2-carboxylate Derivatives

To the solution of ethyl thiooxamate (1 equiv.) in dry DCM (2 mL/mmol), a solution of triethyloxonium tetrafluoroborate (1 equiv.) in dry DCM (2 mL/mmol) was added dropwise, and the resulting mixture was stirred at room temperature for 16 h and then evaporated to dryness. The residue was dissolved in glacial acetic acid (2 mL/mmol) and anhydrous NaOAc (2 equiv.), 2-amino-1-arylethan-1-one hydrochloride (1 equiv.) was added to the solution and the mixture was stirred at 70 °C under an inert atmosphere (argon) for 2 h. The reaction mixture was concentrated under reduced pressure. Then, water was added, and the mixture was neutralised with saturated NaHCO_3_ solution and extracted with EtOAc. The combined organic layers were washed with water and brine, dried over MgSO_4_ and filtered, and the solvent was removed under reduced pressure. The crude product was purified using column chromatography.

#### 5.1.3. General Procedure C for the Ester Hydrolysis

The corresponding ethyl aryl-1*H*-imidazole-carboxylate (1 equiv.) was dissolved/suspended in EtOH (2 mL), and a solution of LiOH ∙ H_2_O (3 equiv.) in water (2 mL) was added to it. The resulting mixture was stirred overnight at 50 °C. Subsequently, the pH was set to 5–6 with 1M HCl solution; then, the solvent was removed under reduced pressure, and the residue was purified using column chromatography.

#### 5.1.4. General Procedure D for the Synthesis of *N*-(2,3,4,6-tetra-*O*-acetyl-β-d-glucopyranosyl)-2-aryl-1*H*-imidazole-4(5)-carboxamides

To the solution of 2-aryl-1*H*-imidazole-4(5)-carboxylic acid (1 equiv.) in dry DMF (1 mL), DIPEA (5 equiv.) and HATU (1.2 equiv.) were added, and the mixture was stirred for 15 min; then, a solution of 2,3,4,6-tetra-*O*-acetyl-β-d-glucopyranosyl amine (**9**) (1.5 equiv.) in dry DMF (1 mL) was added to it. The reaction mixture was stirred overnight at room temperature. Then, the reaction mixture was diluted with water and extracted with EtOAc. The combined organic layers were washed with water and brine, dried over MgSO_4_ and filtered, and the solvent was removed under reduced pressure. The crude product was purified using column chromatography.

#### 5.1.5. General Procedure E for the Synthesis of *N*-(2,3,4,6-tetra-*O*-acetyl-β-d-glucopyranosyl)-4(5)-aryl-1*H*-imidazole-2-carboxamides

To the solution of a 4(5)-aryl-1*H*-imidazole-2-carboxylic acid (1 equiv.) in dry DMF (2 mL), a 50% solution of T3P in DMF (3 equiv.) and dry TEA (3 equiv.) were added, and the resulting mixture was stirred for 15 min. Then, a solution of 2,3,4,6-tetra-*O*-acetyl-β-d-glucopyranosyl amine (1 equiv.) in dry DMF (1 mL) was added to it. The reaction mixture was stirred overnight at room temperature. The reaction mixture was diluted with water and extracted with EtOAc. The combined organic layers were washed with water and brine, dried over MgSO_4_ and filtered, and the solvent was removed under reduced pressure. The crude product was purified using column chromatography.

#### 5.1.6. General Procedure F for Zemplén Deacetylation

To the solution of an *N*-(2,3,4,6-tetra-*O*-acetyl-β-d-glucopyranosyl)-aryl-*1H*-imidazole-carboxamide in dry MeOH (5 mL), 3 drops of 1M NaOMe solution in MeOH were added, and the resulting mixture was stirred at room temperature. After the completion of the reaction monitored using TLC, the pH was set to 6–7 with glacial acetic acid, the mixture was concentrated under reduced pressure and the residue was purified using column chromatography.

### 5.2. Synthesis and Characterisation of the Compounds

#### 5.2.1. Ethyl 2-Phenyl-1*H*-imidazole-4(5)-carboxylate (**4a**) [36]

Prepared from ethyl 2-bromo-1*H*-imidazole-4(5)-carboxylate (**3**) (219 mg, 1 mmol, 1.0 eq.), phenylboronic acid (244 mg, 2 mmol, 2.0 eq.), Pd(amphos)Cl_2_ (35 mg, 0.05 mmol, 0.05 eq.), dry TEA (279 µL, 2 mmol, 2 eq.) and dry 1,4-dioxane (6 mL) according to general procedure A. Purified using column chromatography (eluent: hexane:EtOAc = 2:1 to 1:3 gradient) to give 69 mg (32%) of **4a** as a pale yellow amorphous solid (R_f_ = 0.48, hexane:EtOAc = 1:1). The product was a tautomeric mixture. ^1^H NMR (400 MHz, CDCl_3_) *δ* (ppm): 13.04 and 12.88 (1H, brs and brs, imidazole NH), 7.89–7.87 (m, aromatics), 7.80 (s, imidazole), 7.63–7.61 (m, aromatics), 7.46–7.31 (m, aromatics), 4.25 and 4.15 (2H, q, *J* = 7 Hz CH_2_), 1.26 and 1.18 (3H, t, *J* = 7 Hz, CH_3_). ^13^C NMR (100 MHz, CDCl_3_) *δ* (ppm): 163.0 and 159.9 (C=O), 146.2, 138.3, 136.4, 136.0, 133.8, 129.6, 129.2, 128.9, 128.5, 128.0, 127.8, 127.6, 127.5, 117.4 (aromatics), 60.3 and 59.4 (CH_2_), 14.1 (CH_3_). HRMS positive mode *m*/*z*: calculated C_12_H_13_N_2_O_2_^+^ [M+H]^+^ 217.0972, found 217.0970.

#### 5.2.2. Ethyl 2-(1-Naphthyl)-1*H*-imidazole-4(5)-carboxylate (**4b**)

Prepared from ethyl 2-bromo-1*H*-imidazole-4(5)-carboxylate (**3**) (219 mg, 1 mmol, 1.0 eq.), naphthalene-1-boronic acid (344 mg, 2 mmol, 2.0 eq.), Pd(amphos)Cl_2_ (35 mg, 0.05 mmol, 0.05 eq.), dry TEA (279 µL, 2 mmol, 2 eq.) and dry 1,4-dioxane (6 mL) according to general procedure A. Purified using column chromatography (eluent: hexane:EtOAc = 3:1 to 1:3 gradient) to give 98 mg (37%) of **4b** as a pale yellow amorphous solid (R_f_ = 0.46, hexane:EtOAc = 1:1). The product was a tautomeric mixture. ^1^H NMR (400 MHz, CDCl_3_) *δ* (ppm): 13.28 and 13.00 (1H, brs, imidazole NH), 8.04–7.91 (m, aromatics), 7.78 (d, *J* = 8.5 Hz, aromatics), 7.60–7.41 (m, aromatics), 3.99 and 3.90 (2H, q, *J* = 7.1 Hz, CH_2_), 0.87 and 0.78 (3H, t, *J* = 7.1, Hz CH_3_). ^13^C NMR (100 MHz, CDCl_3_) *δ* (ppm): 162.5 and 159.7 (C=O), 145.4, 138.4, 136.2, 134.6, 133.0, 132.9, 132.2, 131.7, 129.8, 128.9, 128.4, 128.3, 128.2, 128.1, 128.0, 126.5, 126.0, 125.8, 125.6, 125.2, 125.1, 124.9, 124.2, 119.5 (aromatics), 59.8 and 59.0 (CH_2_), 13.6 (CH_3_). HRMS positive mode *m*/*z*: calculated C_16_H_15_N_2_O_2_^+^ [M+H]^+^ 267.1128, found 267.1129.

#### 5.2.3. Ethyl 2-(2-Naphthyl)-1*H*-imidazole-4(5)-carboxylate (**4c**)

Prepared from ethyl 2-bromo-1*H*-imidazole-4(5)-carboxylate (**3**) (219 mg, 1 mmol, 1.0 eq.), naphthalene-2-boronic acid (344 mg, 2 mmol, 2.0 eq.), Pd(amphos)Cl_2_ (35 mg, 0.05 mmol, 0.05 eq.), dry TEA (279 µL, 2 mmol, 2 eq.) and dry 1,4-dioxane (6 mL) according to general procedure A. Purified using column chromatography (eluent: hexane:EtOAc = 3:1 to 1:3 gradient) to give 75 mg (28%) of **4c** as a pale yellow amorphous solid (R_f_ = 0.43, hexane:EtOAc = 1:1). The product was a tautomeric mixture. ^1^H NMR (400 MHz, CDCl_3_) *δ* (ppm): 13.12 and 13.03 (1H, brs, imidazole NH), 8.47 and 8.17 (1H, s, aromatics), 8.04 (d, *J* = 8.7 Hz aromatics), 7.98–7.91 (m, aromatics), 7.86 (s, aromatics), 7.74 (d, *J* = 8.4 Hz, aromatics), 7.63 (s, aromatics), 7.58–7.55 and 7.53–7.51 (m, aromatics), 7.26 (brs, aromatics), 4.28 and 4.17 (2H, q, *J* = 7.1 Hz, CH_2_), 1.26 and 1.17 (3H, t, *J* = 7.1 Hz, CH_3_). ^13^C NMR (100 MHz, CDCl_3_) *δ* (ppm): 163.0 and 160.0 (C=O), 145.9, 138.4, 136.4, 136.2, 132.6, 132.5, 132.4, 131.3, 128.2, 128.1, 127.9, 127.6, 127.5, 127.2, 127.1, 126.9, 126.7, 126.5, 126.3, 126.2, 117.8, 115.7 (aromatics), 60.4 and 59.5 (CH_2_), 14.1 (CH_3_). HRMS positive mode *m*/*z*: calculated C_16_H_15_N_2_O_2_^+^ [M+H]^+^ 267.1128, found 267.1127.

#### 5.2.4. 2-Phenyl-1*H*-imidazole-4(5)-carboxylic Acid (**5a**) [34]

Prepared from **4a** (135 mg, 0.62 mmol, 1.0 eq.), LiOH ∙ H_2_O (78 mg, 1.86 mmol, 3.0 eq.) in EtOH:H_2_O = 1:1 (4 mL) according to general procedure C. Purified using column chromatography (eluent: DCM:MeOH = 10:1) to give 80 mg (68%) of **5a** as a white solid (R_f_ = 0.13 DCM:MeOH = 10:1). The product was a tautomeric mixture. ^1^H NMR (400 MHz, DMSO-*d*_6_) *δ* (ppm): 12.87 (2H, brs, imidazole NH + COOH), 7.89 (2H, brs, aromatics), 7.81 (1H, s, imidazole CH), 7.39 (2H, pt, *J* = 7.2 Hz, aromatics), 7.32 (1H, t, *J* = 7.1 Hz, aromatics). ^13^C NMR (100 MHz, DMSO-*d*_6_) *δ* (ppm): 145.3, 137.0, 133.9, 128.9, 2 × 127.7, 118.6. HRMS positive mode *m*/*z*: calculated C_10_H_9_N_2_O_2_^+^ [M+H]^+^ 189.0659, found 189.0659.

#### 5.2.5. 2-(1-Naphthyl)-1*H*-imidazole-4(5)-carboxylic Acid (**5b**)

Prepared from **4b** (98 mg, 0.37 mmol, 1.0 eq.) and LiOH ∙ H_2_O (47 mg, 1.11 mmol, 3.0 eq.) in EtOH:H_2_O = 1:1 (4 mL) according to general procedure C. Purified using column chromatography (eluent: DCM:MeOH = 10:1) to give 55 mg (63%) of **5b** as a white solid (R_f_ = 0.14 DCM:MeOH = 10:1). The product was a tautomeric mixture. ^1^H NMR (400 MHz, DMSO-*d*_6_) *δ* (ppm): 13.13 and 12.91 (1H, brs, imidazole NH), 12.34 (1H, brs, COOH), 7.95–7.45 (8H, m, aromatics). ^13^C NMR (100 MHz, DMSO-*d*_6_) *δ* (ppm): 161.2 (C=O), 145.0, 137.9, 133.0, 132.5, 131.8, 128.9, 128.4, 128.0, 126.6, 126.1, 125.6, 125.1 (aromatics). HRMS positive mode *m*/*z*: calculated C_14_H_11_N_2_O_2_^+^ [M+H]^+^ 239.0815, found 239.0814.

#### 5.2.6. 2-(2-Naphthyl)-1*H*-imidazole-4(5)-carboxylic Acid (**5c**)

Prepared from **4c** (75 mg, 0.28 mmol, 1.0 eq.) and LiOH ∙ H_2_O (35 mg, 0.84 mmol, 3.0 eq.) in EtOH:H_2_O = 1:1 (4 mL) according to general procedure C. Purified using column chromatography (eluent: DCM:MeOH = 10:1) to give 41 mg (61%) of **5c** as a white solid (R_f_ = 0.12 DCM:MeOH = 10:1). The product was a tautomeric mixture. ^1^H NMR (400 MHz, DMSO-*d*_6_) *δ* (ppm): 12.98 (2H, brs, imidazole NH, COOH), 8.49 (1H, brs, aromatics), 8.08 (1H, brs, aromatics), 7.94–7.88 (4H, m, aromatics), 7.53–7.51 (2H, m, aromatics). ^13^C NMR (100 MHz, DMSO-*d*_6_) *δ* (ppm): 161.4 (C=O), 137.9, 132.6, 132.5, 128.2, 127.8, 127.5, 127.2, 126.9, 126.2. HRMS positive mode *m*/*z*: calculated C_14_H_11_N_2_O_2_^+^ [M+H]^+^ 239.0815, found 239.0818.

#### 5.2.7. Ethyl 4(5)-Phenyl-1*H*-imidazole-2-carboxylate (**7a**) [32]

Prepared from ethyl thiooxamate (**6**) (266 mg, 2.0 mmol, 1.0 eq.), triethyloxonium tetrafluoroborate (380 mg, 2.0 mmol, 1 eq.), anhydrous NaOAc (328 mg, 4.0 mmol, 2 eq.) and 2-amino-1-phenylethan-1-one hydrochloride (344 mg, 2.0 mmol, 1 eq.) according to general procedure B. Purified using column chromatography (eluent: hexane:EtOAc = 3:1 to 1:1 gradient) to give 246 mg (57%) of **7a** as a yellowish foam (R_f_ = 0.44, hexane:EtOAc = 1:1). The product was a tautomeric mixture. ^1^H NMR (360 MHz, CDCl_3_) δ (ppm): 7.78 (2H, d, *J* = 7.7 Hz, aromatics), 7.56 (1H, s, imidazole CH), 7.37 (2H, pt, *J* = 7.7 and 7.1 Hz, aromatics), 7.29 (1H, t, *J* = 7.1 Hz, aromatics), 4.38 (2H, q, *J* = 7.1 Hz, CH_2_), 1.30 (3H, t, *J* = 7.1 Hz, CH_3_). ^13^C NMR (90 MHz, CDCl_3_) *δ* (ppm): 159.4, 138.1, 128.8, 128.0, 2 × 125.5 (aromatics), 62.0 (CH_2_), 14.2 (CH_3_) (2C are missing). HRMS positive mode *m*/*z*: calculated C_12_H_13_N_2_O_2_^+^ [M+H]^+^ 217.0972, found 217.0976.

#### 5.2.8. Ethyl 4(5)-(1-Naphthyl)-1*H*-imidazole-2-carboxylate (**7b**)

Prepared from ethyl thiooxamate (**6**) (266 mg, 2.0 mmol, 1.0 eq.), triethyloxonium tetrafluoroborate (380 mg, 2.0 mmol, 1 eq.), anhydrous NaOAc (328 mg, 4.0 mmol, 2 eq.) and 2-amino-1-(1-naphthyl)ethan-1-one hydrochloride (444 mg, 2.0 mmol, 1 eq.) according to general procedure B. Purified using column chromatography (eluent: hexane:EtOAc = 3:1 to 1:1 gradient) to give 156 mg (36%) of **7b** as a yellowish foam (R_f_ = 0.45, hexane:EtOAc = 1:1). The product was a tautomeric mixture. ^1^H NMR (400 MHz, CDCl_3_) *δ* (ppm): 10.90 (1H, brs, NH), 8.39–8.36 and 8.08–8.06 (1H, m, aromatic), 7.92–7.83 (2H, m, aromatics), 7.73 and 7.57–7.47 (5H, d and m, *J* = 7.1 Hz, aromatics), 4.42 and 4.36 (2H, q, *J* = 7.1 Hz, CH_2_), 1.40 and 1.39 (3H, t, *J* = 7.1 Hz, CH_3_). ^13^C NMR (100 MHz, CDCl_3_) *δ* (ppm): 159.5 + 159.4 (C=O), 143.6, 138.2, 137.9, 134.0, 133.5, 131.5, 131.3, 131.2, 131.1, 129.7, 128.8, 128.5 (2x), 127.5, 127.5, 127.3, 126.7, 126.6, 126.5, 125.9, 125.7, 125.5, 125.4, 124.9, 118.5 (aromatics), 62.2 (CH_2_), 14.5 and 14.4 (CH_3_). HRMS positive mode *m*/*z*: calculated C_16_H_15_N_2_O_2_^+^ [M+H]^+^ 267.1128, found 267.1126.

#### 5.2.9. Ethyl 4(5)-(2-Naphthyl)-1*H*-imidazole-2-carboxylate (**7c**)

Prepared from ethyl thiooxamate (**6**) (266 mg, 2.0 mmol, 1.0 eq.), triethyloxonium tetrafluoroborate (380 mg, 2.0 mmol, 1 eq.), anhydrous NaOAc (328 mg, 4.0 mmol, 2 eq.) and 2-amino-1-(2-naphthyl)ethan-1-one hydrochloride (444 mg, 2.0 mmol, 1 eq.) according to general procedure B. Purified using column chromatography (eluent: hexane:EtOAc = 2:1 to 1:1 gradient) to give 402 mg (75%) of **7c** as a yellowish foam (R_f_ = 0.47, hexane:EtOAc = 1:1). The product was a tautomeric mixture. ^1^H NMR (400 MHz, CDCl_3_) *δ* (ppm): 11.10 and 10.88 (1H, brs, NH), 8.40 and 8.06 (1H, brs, aromatics), 7.93–7.81 and 7.73 (4H, m and d, *J* = 8.5 Hz, aromatics), 7.66 and 7.61 (1H, s, aromatic), 7.54–7.43 (2H, m, aromatics), 4.49 and 4.46 (2H, q, *J* = 6.8 Hz, CH_2_), 1.43 and 1.42 (3H, t, *J* = 6.8 Hz, H_3_). ^13^C NMR (100 MHz, CDCl_3_) *δ* (ppm): 159.4 + 159.3 (C=O), 144.6 (2x), 138.4, 138.2, 135.2, 133.7, 133.5, 133.2, 133.1, 130.5, 129.2, 128.8, 128.4, 128.3, 128.2, 128.0, 127.8, 127.1, 126.9, 126.4, 126.0, 124.2 (2x), 123.8, 123.3, 115.8 (aromatics), 62.3 + 62.2 (CH_2_), 14.5 + 14.4 (CH_3_). HRMS positive mode *m*/*z*: calculated C_16_H_15_N_2_O_2_^+^ [M+H]^+^ 267.1128, found 267.1127.

#### 5.2.10. 4(5)-Phenyl-1*H*-imidazole-2-carboxylic Acid (**8a**) [35]

Prepared from **7a** (120 mg, 0.55 mmol, 1.0 eq.) and LiOH ∙ H_2_O (69 mg, 1.65 mmol, 3.0 eq.) in THF:H_2_O = 1:1 (4 mL) according to general procedure C. Purified using column chromatography (eluent: DCM:MeOH = 10:1) to give 80 mg (77%) of **8a** as a white solid (R_f_ = 0.15, DCM:MeOH = 10:1). The product was a tautomeric mixture. ^1^H NMR (360 MHz, MeOD) *δ* (ppm): 7.71 (2H, d, *J* = 7.7 Hz, aromatics), 7.45 (1H, brs, imidazole CH), 7.37 (2H, pt, *J* = 7.7, 7.4 Hz, aromatics), 7.27 (1H, t, *J* = 7.4 Hz, aromatics). ^13^C NMR (90 MHz, MeOD) *δ* (ppm): 164.3 (C=O), 145.7, 139.3, 132.7, 129.8, 128.7, 126.4, 119.4 (aromatics). HRMS positive mode *m*/*z*: calculated C_10_H_9_N_2_O_2_^+^ [M+H]^+^ 189.0659, found 189.0661.

#### 5.2.11. 4(5)-(1-Naphthyl)-1*H*-imidazole-2-carboxylic Acid (**8b**)

Prepared from **7b** (150 mg, 0.56 mmol, 1.0 eq.) and LiOH ∙ H_2_O (71 mg, 1.68 mmol, 3.0 eq.) in THF:H_2_O = 1:1 (4 mL) according to general procedure C. Purified using column chromatography (eluent: DCM:MeOH = 10:1) to give 101 mg (75%) of **8b** as a white solid (R_f_ = 0.18, DCM:MeOH = 10:1). ^1^H NMR (400 MHz, DMSO-*d*_6_) *δ* (ppm): 8.66–8.63 (1H, m, aromatics), 7.95–7.93 (1H, m, aromatics), 7.86 (1H, d, *J* = 8.2 Hz, aromatics), 7.71 (1H, d, *J* = 7.1 Hz, aromatics), 7.54–7.50 (3H, m, aromatics), 7.48 (1H, s, imidazole CH). ^13^C NMR (100 MHz, DMSO-*d*_6_) *δ* (ppm): 161.2 (C=O), 143.4, 138.6, 133.6, 131.3, 130.6, 128.2, 127.3, 126.3, 126.2, 126.1, 125.7, 125.5 (aromatics), 118.9 (imidazole CH). HRMS positive mode *m*/*z*: calculated C_14_H_11_N_2_O_2_^+^ [M+H]^+^ 239.0815, found 239.0814.

#### 5.2.12. 4(5)-(2-Naphthyl)-1*H*-imidazole-2-carboxylic Acid (**8c**)

Prepared from **7c** (200 mg, 0.75 mmol, 1.0 eq.) and LiOH ∙ H_2_O (95 mg, 2.25 mmol, 3.0 eq.) in THF:H_2_O = 1:1 (4 mL) according to general procedure C. Purified using column chromatography (eluent: DCM:MeOH: = 10:1) to give 147 mg (82%) of **8c** as a white solid (R_f_ = 0.19, DCM:MeOH = 10:1). ^1^H NMR (400 MHz, DMSO-*d*_6_) *δ* (ppm): 8.36 (1H, brs, aromatics), 7.98 (1H, dd, *J* = 8.6, 1.3 Hz, aromatics), 7.90–7.85 (3H, m, aromatics), 7.72 (1H, s, imidazole CH), 7.50–7.41 (2H, m, aromatics). ^13^C NMR (100 MHz, DMSO-*d*_6_) *δ* (ppm): 161.4 (C=O), 144.4, 139.3, 133.4, 131.9, 131.6, 127.9, 127.8, 127.6, 126.2, 125.3, 123.8, 122.2, 116.6 (imidazole CH). HRMS positive mode *m*/*z*: calculated C_14_H_11_N_2_O_2_^+^ [M+H]^+^ 239.0815, found 239.0816.

#### 5.2.13. *N*-(2,3,4,6-Tetra-*O*-acetyl-β-**d**-glucopyranosyl)-2-phenyl-1*H*-imidazole-4(5)-carboxamide (**10a**)

Prepared from 2-phenyl-1*H*-imidazole-4(5)-carboxylic acid (**5a**) (40 mg, 0.213 mmol, 1 eq.), HATU (243 mg, 0.639 mmol, 3 eq.), DIPEA (111 μL, 0.639 mmol, 3 eq.) and 2,3,4,6-tetra-*O*-acetyl-β-d-glucopyranosylamine (**9**) (74 mg, 0.213 mmol, 1 eq.) according to general procedure D. Purified using column chromatography (eluent: hexane:EtOAc = 3:1 to 0:1 gradient) to give 51 mg (46%) of **10a** as a white solid (R_f_ = 0.59, EtOAc); [α]_D =_ -28 (c 0.15, CHCl_3_). ^1^H NMR (400 MHz, CDCl_3_) *δ* (ppm): 10.22 (1H, brs, imidazole NH), 7.95 (1H, d, *J* = 9.9 Hz, CONH), 7.89–7.86 (2H, m, aromatics), 7.72 (1H, d, *J* = 2.3 Hz, imidazole CH), 7.49–7.40 (3H, m, aromatics), 5.48 (1H, pt, *J* = 9.6 Hz, H-1), 5.36 (1H, pt, *J* = 9.6 Hz, H-3), 5.16 (1H, pt, *J* = 9.6 Hz, H-2), 5.14 (1H, pt, *J* = 9.6 Hz, H-4), 4.31 (1H, dd, *J* = 12.5, 4.3 Hz, H-6), 4.09 (1H, dd, *J* = 12.5, 2.1 Hz, H-6′), 3.87 (1H, ddd, *J* = 9.6, 4.3, 2.1 Hz, H-5), 2.07 (3H, s, CH_3_CO), 2.05 (3H, s, CH_3_CO), 2.04 (3H, s, CH_3_CO), 1.98 (3H, s, CH_3_CO). ^13^C NMR (100 MHz, CDCl_3_) *δ* (ppm): 170.9, 170.4, 170.2, 169.7 (C=O_OAc_), 163.4 (C=O_CONH_), 146.7, 136.4, 129.7, 129.3, 129.2, 125.7, 120.7 (aromatics), 78.1 (C-1), 73.8 (C-5), 73.3 (C-3), 70.6 (C-2), 68.3 (C-4), 61.9 (C-6), 20.9, 20.8, 20.8, 20.8 (CH_3_CO). HRMS positive mode *m*/*z*: calculated C_24_H_28_N_3_O_10_^+^ [M+H]^+^ 518.1769, found 518.1770.

#### 5.2.14. *N*-(2,3,4,6-Tetra-*O*-acetyl-β-d-glucopyranosyl)-2-(1-naphthyl)-1*H*-imidazole-4(5)-carboxamide (**10b**)

Prepared from 2-(1-naphthyl)-1*H*-imidazole-4(5)-carboxylic acid (**5b**) (50 mg, 0.210 mmol, 1 eq.), HATU (240 mg, 0.630 mmol, 3 eq.), DIPEA (110 μL, 0.630 mmol, 3 eq.) and 2,3,4,6-tetra-*O*-acetyl-β-d-glucopyranosylamine (**9**) (73 mg, 0.210 mmol, 1 eq.) according to general procedure D. Purified using column chromatography (eluent: hexane:EtOAc = 3:1 to 0:1 gradient) to give 41 mg (34%) of **10b** as a white solid (R_f_ = 0.60, EtOAc); [α]_D_ = -4 (c 0.14, CHCl_3_). ^1^H NMR (400 MHz, CDCl_3_) *δ* (ppm): 9.54 (1H, brs, imidazole NH), 7.99 (1H, d, *J* = 9.6 Hz, CONH), 7.95 (1H, d, *J* = 8.1 Hz, aromatics), 7.91 (1H, d, *J* = 8.1 Hz, aromatics), 7.62–7.58 (3H, m, aromatics), 7.55–7.42 (3H, m, aromatics), 5.25 (1H, pt, *J* = 9.6 Hz, H-1), 5.24 (1H, t, *J* = 9.6 Hz, H-3), 5.09 (1H, pt, *J* = 9.6 Hz, H-2), 5.08 (1H, pt*, J* = 9.6 Hz, H-4), 4.22 (1H, dd, *J* = 12.4, 4.1 Hz, H-6), 4.01 (1H, dd, *J* = 12.4, 2.0 Hz, H-6), 4.01 (1H, ddd, *J* = 10.0, 4.1, 2.0 Hz, H-5), 2.04 (3H, s, CH_3_CO), 2.01 (3H, s, CH_3_CO), 2.01 (3H, s, CH_3_CO), 2.01 (3H, s, CH_3_CO). ^13^C NMR (100 MHz, CDCl_3_) *δ* (ppm): 170.9, 170.7, 170.2, 169.7 (C=O_OAc_), 162.7 (C=O_CONH_), 134.2, 133.7, 133.0, 132.0, 131.7, 130.1, 129.1, 128.8, 126.9, 126.7, 126.4, 125.2, 125.1 (aromatics), 78.0 (C-1), 73.5 (C-5), 73.3 (C-3), 70.5 (C-2), 68.3 (C-4), 61.8 (C-6), 20.9, 20.9, 20.8, 20.8 (CH_3_CO). HRMS positive mode *m*/*z*: calculated C_28_H_30_N_3_O_10_^+^ [M+H]^+^ 568.1926, found 568.1926.

#### 5.2.15. *N*-(2,3,4,6-Tetra-*O*-acetyl-β-d-glucopyranosyl)-2-(2-naphthyl)-1*H*-imidazole-4(5)-carboxamide (**10c**)

Prepared from 2-(2-naphthyl)-1*H*-imidazole-4(5)-carboxylic acid (**5c**) (40 mg, 0.168 mmol, 1 eq.), HATU (192 mg, 0.504 mmol, 3 eq.), DIPEA (88 μL, 0.504 mmol, 3 eq.) and 2,3,4,6-tetra-*O*-acetyl-β-d-glucopyranosylamine (**9**) (58 mg, 0.168 mmol, 1 eq.) according to general procedure D. Purified using column chromatography (eluent: hexane:EtOAc = 3:1 to 0:1 gradient) to give 45 mg (47%) of **10c** as a white solid (R_f_ = 0.69, EtOAc); [α]_D_ = -11 (c 0.16, CHCl_3_). ^1^H NMR (400 MHz, CDCl_3_) *δ* (ppm): 9.77 (1H, brs, imidazole NH), 8.16 (1H, brs, aromatics), 8.12 (1H, d, *J* = 9.7 Hz, CONH), 7.85–7.82 (4H, m, aromatics), 7.56 (1H, s, imidazole CH), 7.53–7.47 (2H, m, aromatics), 5.39 (1H, pt, *J* = 9.7 Hz, H-1), 5.30 (1H, t, *J* = 9.6 Hz, H-3), 5.12 (1H, pt, *J* = 9.6 Hz, H-2), 5.10 (1H, pt, *J* = 9.6 Hz, H-4), 4.25 (1H, dd, *J* = 12.5, 4.2 Hz, H-6), 4.06 (1H, dd, *J* = 12.5, 2.0 Hz, H-6′), 3.76 (1H, ddd, *J* = 9.6, 4.2, 2.0 Hz, H-5), 2.04 (3H, s, CH_3_CO), 2.02 (6H, s, 2 × CH_3_CO), 2.00 (3H, s, CH_3_CO). ^13^C NMR (100 MHz, CDCl_3_) *δ* (ppm): 170.9, 170.7, 170.2, 169.7 (C=O_OAc_), 163.3 (C=O_CONH_), 135.2, 134.1, 133.5, 133.0, 129.8, 128.5, 128.2, 127.9, 127.1, 126.7, 126.5, 126.3 (aromatics), 78.1 (C-1), 73.5 (C-5), 73.3 (C-3), 70.5 (C-2), 68.4 (C-4), 61.9 (C-6), 20.9, 20.8, 20.8, 20.8 (CH_3_CO). HRMS positive mode *m*/*z*: calculated C_28_H_30_N_3_O_10_^+^ [M+H]^+^ 568.1926, found 568.1925.

#### 5.2.16. *N*-(2,3,4,6-Tetra-*O*-acetyl-β-d-glucopyranosyl)-4(5)-phenyl-1*H*-imidazole-2-carboxamide (**11a**)

Prepared from 4(5)-phenyl-1*H*-imidazole-2-carboxylic acid (**8a**) (69 mg, 0.367 mmol, 1 eq.), a 50% solution of T3P in DMF (797 μL, 1.101 mmol, 3 eq.), dry TEA (153 μL, 1.101 mmol, 3 eq.) and 2,3,4,6-tetra-*O*-acetyl-β-d-glucopyranosylamine (**9**) (127 mg, 0.367 mmol, 1 eq.) according to general procedure E. Purified using column chromatography (eluent: hexane:EtOAc = 3:1 to 0:1 gradient) to give 59 mg (31%) of **11a** as a white solid (R_f_ = 0.45, hexane:EtOAc = 1:2); [α]_D_ = -21 (c 0.10, CHCl_3_). ^1^H NMR (400 MHz, DMSO-*d*_6_) *δ* (ppm): 11.30 and 10.87 (1H, brs, imidazole NH), 8.04 and 8.02 (1H, d, *J* = 9.1 Hz, CONH), 7.82–7.80 and 7.62–7.60 (2H, m, aromatics), 7.49–7.45 and 7.42–7.38 (2H, m, aromatics), 7.46 and 7.40 (1H, s, imidazole CH), 7.42–7.38 and 7.31–7.28 (1H, m, aromatics), 5.49–5.37 and 5.19–5.10 (2H, m, H-1–H-4), 4.30 and 4.25 (1H, dd, *J* = 12.6, 4.2 Hz, H-6), 4.11 and 4.07 (1H, dd, *J* = 12.6, 2.2 Hz, H-6′), 3.88 and 3.76 (1H, ddd, *J* = 10.1, 4.2, 2.2 Hz, H-5), 2.08 (3H, s, CH_3_CO), 2.05 (3H, s, CH_3_CO), 2.00 (3H, s, CH_3_CO), 1.99 (3H, s, CH_3_CO). ^13^C NMR (100 MHz, DMSO-*d*_6_) *δ* (ppm): 170.8 and 170.8, 170.5 and 170.3, 170.3 and 170.3, 169.6 and 169.6, 159.2 and 159.1 (C=O), 143.6 and 135.4 (imidazole C-2), 140.1 and 139.6 (imidazole C-4(5)), 133.2 and 128.9, 129.4 and 128.9, 128.8 and 127.8, 125.5 and 125.4 (aromatics), 127.6 and 115.5 (imidazole CH), 78.2 and 78.1 (C-1), 73.8 and 73.7 (C-5), 73.2 and 73.1, 70.5 and 70.5, 68.2 (C-2, C-3, C-4), 61.8 (C-6), 20.9, 2 × 20.8, 20.7 (CH_3_CO). HRMS positive mode *m*/*z*: calculated C_24_H_28_N_3_O_10_^+^ [M+H]^+^ 518.1769, found 518.1768.

#### 5.2.17. *N*-(2,3,4,6-Tetra-*O*-acetyl-β-d-glucopyranosyl)-4(5)-(1-naphthyl)-1*H*-imidazole-2-carboxamide (**11b**)

Prepared from 4(5)-(1-naphthyl)-1*H*-imidazole-2-carboxylic acid (**8b**) (70 mg, 0.294 mmol, 1 eq.), a 50% solution of T3P in DMF (639 μL, 0.882 mmol, 3 eq.), dry TEA (123 μL, 0.882 mmol, 3 eq.) and 2,3,4,6-tetra-*O*-acetyl-β-d-glucopyranosylamine (**9**) (102 mg, 0.294 mmol, 1 eq.) according to general procedure E. Purified using column chromatography (eluent: hexane:EtOAc = 3:1 to 0:1 gradient) to give 41 mg (25%) of **11b** as a white solid (R_f_ = 0.46, hexane:EtOAc = 1:2); [α]_D_ = -30 (c 0.15, CHCl_3_). ^1^H NMR (400 MHz, CDCl_3_) *δ* (ppm): 11.86 and 11.01 (1H, brs, imidazole NH), 8.49 (d, *J* = 7.8 Hz), 8.14–7,48 (8H, m, CONH and aromatics), 7.46 and 7.36 (1H, d, *J* = 2.2 and 1.3 Hz, imidazole CH), 5.38–5.25 and 5.13–4.94 (2H, m, H-1, H-4), 4.25 and 4.10 (1H, dd, *J* = 12.5, 4.1 Hz, H-6), 4.06 and 3.90 (1H, dd, *J* = 12.5, 2.0 Hz, H-6′), 3.75 and 3.30 (1H, ddd, *J* = 10.1, 4.1, 2.0 Hz, H-5), 2.09 + 2 × 2.06, 2.05, 2.03, 2.02, 2.01, 2.00 (12H, 6 × s, CH_3_CO). ^13^C NMR (100 MHz, CDCl_3_) *δ* (ppm): 170.8, 170.8, 170.5, 170.3, 170.3, 170.2, 169.6, 169.6 (C=O_OAc_), 159.3, 159.0 (C=O_CONH_), 143.0, 140.0, 139.5, 134.1, 133.9, 133.9, 131.3, 131.2, 130.7, 130.4, 129.7, 128.6, 128.6, 127.7, 127.2, 127.1, 127.0, 126.7, 126.6, 126.0, 125.9, 125.5, 125.5, 125.3, 118.6 (aromatics), 78.1 and 78.0 (C-1), 73.7 and 73.5 (C-5), 73.2 and 73.0, 70.5 and 70.3, 68.2 and 68.1 (C-2–C-4), 61.7 and 61.5 (C-6), 2 × 20.9, 6 × 20.8 (CH_3_CO). HRMS positive mode *m*/*z*: calculated C_28_H_30_N_3_O_10_^+^ [M+H]^+^ 568.1926, found 568.1924.

#### 5.2.18. *N*-(2,3,4,6-Tetra-*O*-acetyl-β-d-glucopyranosyl)-4(5)-(2-naphthyl)-1*H*-imidazole-2-carboxamide (**11c**)

Prepared from 4(5)-(2-naphthyl)-1*H*-imidazole-2-carboxylic acid (**8c**) (70 mg, 0.294 mmol, 1 eq.), a 50% solution of T3P in DMF (639 μL, 0.882 mmol, 3 eq.), dry TEA (123 μL, 0.882 mmol, 3 eq.) and 2,3,4,6-tetra-*O*-acetyl-β-d-glucopyranosylamine (**9**) (102 mg, 0.294 mmol, 1 eq.) according to general procedure E. Purified using column chromatography (eluent: hexane:EtOAc = 3:1 to 0:1 gradient) to give 29 mg (17%) of **11c** as a white solid (R_f_ = 0.48, hexane:EtOAc = 1:2); [α]_D_ = -33 (c 0.12, CHCl_3_). ^1^H NMR (400 MHz, CDCl_3_) *δ* (ppm): 11.62 and 10.84 (1H, brs, imidazole NH), 8.31 and 8.11 (1H, brs, aromatics), 8.04 (1H, d, *J* = 9.8 Hz, CONH), 7.92–7.44 (7 H, m, aromatics), 5.48 and 5.38 (1H, t, *J* = 9.6 Hz, H-1), 5.42–5.01 (3H, m, H-2 H-3, H-4), 4.28 and 4.11 (1H, dd, *J* = 12.5, 4.2 Hz, H-6), 4.08 and 3.92 (1H, dd, *J* = 12.5, 2.0 Hz, H-6′), 3.81 and 3.38 (1H, ddd, *J* = 10.1, 4.2, 2.0 Hz, H-5), 2.08, 2.06, 2.05, 2 × 2.04, 2 × 2.02, 2.01 (CH_3_CO). ^13^C NMR (100 MHz, CDCl_3_) *δ* (ppm): 170.8, 170.8, 170.5, 170.4, 170.3, 170.2, 169.6, 169.6 (C=O_OAc_), 159.3, 159.1 (C=O_CONH_), 143.7, 140.2, 139.8, 135.6, 133.8, 133.5, 133.2, 133.1, 130.6, 129.1, 128.5, 128.3, 128.1, 128.1, 128.0, 127.9, 127.4, 127.0, 126.5, 126.3, 126.0, 124.4, 123.9, 123.7, 123.6, 115.9 (aromatics), 78.1 (C-1), 73.8 + 73.5 (C-5), 73.2 + 73.0, 70.5, 68.2 and 68.0 (C-2–C-4), 61.8 + 61.6 (C-6), 2 × 20.9, 5 × 20.8, 20.7 (CH_3_CO). HRMS positive mode *m*/*z*: calculated C_28_H_30_N_3_O_10_^+^ [M+H]^+^ 568.1926, found 568.1926.

#### 5.2.19. *N*-(β-d-Glucopyranosyl)-2-phenyl-1*H*-imidazole-4(5)-carboxamide (**1a**)

Prepared from *N*-(2,3,4,6-tetra-*O*-acetyl-β-d-glucopyranosyl)-2-phenyl-*1H*-imidazole-4(5)-carboxamide (**10a**) (45 mg, 0.087 mmol) according to general procedure F. Purified using column chromatography (eluent: DCM:MeOH = 5:1) to give 25 mg (83%) of **1a** as a white solid (R_f_ = 0.26, DCM:MeOH = 5:1); [α]_D_ = +1 (c 0.16, MeOH). ^1^H NMR (400 MHz, DMSO-*d*_6_) *δ* (ppm): 13.05 (1H, brs, imidazole NH), 8.06–8.02 (3H, m, CONH + aromatics), 7.85 (1H, s, imidazole CH), 7.48 (2H, pt, *J* = 7.5 Hz, aromatics), 7.40 (1H, t, *J* = 7.5 Hz, aromatics), 5.04–5.02 (2H, m, 2 × OH), 4.95–4.91 (2H, m, H-1, OH), 4.51 (1H, t, *J* = 5.7 Hz, OH), 3.66 (1H, ddd, *J* = 11.8, 5.6, 1.6 Hz, H-6), 3.46–3.41 (1H, m, H-6′), 3.31–3.09 (4H, m, H-2–H-5). ^13^C NMR (100 MHz, DMSO-*d*_6_) *δ* (ppm): 162.3 (C=O_CONH_), 145.6, 136.5, 129.9, 128.8, 2 × 125.3, 121.2 (aromatics), 79.3 (C-1), 78.6, 77.5, 72.4, 70.0 (C-2–C-5), 61.0 (C-6). HRMS positive mode *m*/*z*: calculated C_16_H_20_N_3_O_6_^+^ [M+H]^+^ 350.1347, found 350.1347.

#### 5.2.20. *N*-(β-d-Glucopyranosyl)-2-(1-naphthyl)-1*H*-imidazole-4(5)-carboxamide (**1b**)

Prepared from *N*-(2,3,4,6-tetra-*O*-acetyl-β-d-glucopyranosyl)-2-(1-naphthyl)-*1H*-imidazole-4(5)-carboxamide (**10b**) (35 mg, 0.062 mmol) according to general procedure F. Purified using column chromatography (eluent: DCM:MeOH = 5:1) to give 21 mg (84%) of **1b** as a white solid (R_f_ = 0.20 DCM:MeOH = 5:1); [α]_D_ = -14 (c 0.13, MeOH). ^1^H NMR (400 MHz, DMSO-*d*_6_) *δ* (ppm): 12.93 (1H, s, imidazole NH), 8.05–7.99 (3H, m, CONH and aromatics), 7.92 (1H, s, imidazole CH), 7.59–7.45 (5H, m, aromatics), 5.03–5.00 (2H, m, 2 × OH), 4.87 (1H, d, *J* = 4.8 Hz, OH), 4.69 (1H, t, *J* = 9.1 Hz, H-1), 4.46 (1H, t, *J* = 5.9 Hz, OH), 3.58 (1H, ddd, *J* = 12.0, 5.7, 1.8 Hz, H-6), 3.41–3.36 (1H, m, H-6′), 3.23–3.00 (4H, m, H-2, H-3, H-4, H-5). ^13^C NMR (100 MHz, DMSO-*d*_6_) *δ* (ppm): 162.2 (C=O_CONH_), 135.1, 133.0, 131.9, 128.8, 128.7, 128.4, 128.1, 126.4, 125.9, 125.6, 125.0 (aromatics), 79.2 (C-1), 78.5, 77.5, 72.3, 69.9 (C-2–C-5), 60.8 (C-6). HRMS positive mode *m*/*z*: calculated C_20_H_22_N_3_O_6_^+^ [M+H]^+^ 400.1503, found 400.1505.

#### 5.2.21. *N*-(β-d-Glucopyranosyl)-2-(2-naphthyl)-1*H*-imidazole-4(5)-carboxamide (**1c**)

Prepared from *N*-(2,3,4,6-tetra-*O*-acetyl-β-d-glucopyranosyl)-2-(1-naphthyl)-*1H*-imidazole-4(5)-carboxamide (**10c**) (40 mg, 0.070 mmol) according to general procedure F. Purified using column chromatography (eluent: DCM:MeOH = 5:1) to give 24 mg (86%) of **1c** as a white solid (R_f_ = 0.22 DCM:MeOH = 5:1); [α]_D_ = -34 (c 0.16, MeOH). ^1^H NMR (400 MHz, DMSO-*d*_6_) *δ* (ppm): 13.01 (1H, brs, imidazole NH), 8.41 (1H, brs, aromatics), 8.16 (1H, d, *J* = 9.2 Hz, CONH), 8.00–7.92 (4H, m, aromatics), 7.90 (1H, s, imidazole CH), 7.57–7.53 (2H, m, aromatics), 5.06–5.02 (2H, m, 2 × OH), 4.92 (1H, d, *J* = 5.0 Hz, OH), 4.88 (1H, t, *J* = 9.1 Hz, H-1), 4.50 (1H, t, *J* = 5.9 Hz, OH), 3.64 (1H, ddd, *J* = 11.7, 5.4, 1.5 Hz, H-6), 3.46–3.40 (1H, m, H-6′), 3.26–3.08 (4H, m, H-2–H-5). ^13^C NMR (100 MHz, DMSO-*d*_6_) *δ* (ppm): 162.8 (C=O_CONH_), 135.4, 2 × 132.5, 128.2, 127.8, 127.5, 127.1, 127.0, 126.5, 126.4 (aromatics), 79.4 (C-1), 78.6, 77.5, 72.5, 70.0 (C-2–C-5), 61.0 (C-6). HRMS positive mode *m*/*z*: calculated C_20_H_22_N_3_O_6_^+^ [M+H]^+^ 400.1503, found 400.1507.

#### 5.2.22. *N*-(β-d-Glucopyranosyl)-4(5)-phenyl-1*H*-imidazole-2-carboxamide (**2a**)

Prepared from *N*-(2,3,4,6-tetra-*O*-acetyl-β-d-glucopyranosyl)-4(5)-phenyl-*1H*-imidazole-2-carboxamide (**11a**) (50 mg, 0.097 mmol) according to general procedure F. Purified using column chromatography (eluent: DCM:MeOH = 5:1) to give 30 mg (88%) of **2a** as a white solid (R_f_ = 0.62, DCM:MeOH = 7:3); [α]_D_ = +3 (c 0.11, MeOH). ^1^H NMR (400 MHz, CDCl_3_) *δ* (ppm): 13.50 and 13.26 (1H, brs, imidazole NH), 8.54 (1H, d, *J* = 9.5 Hz, CONH), 7.89 and 7.86 (2H, d, *J* = 7.7 Hz, aromatics), 7.84 and 7.56 (1H, s, imidazole CH), 7.41 and 7.38 (2H, t, *J* = 7.7 Hz, aromatics), 7.29 and 7.24 (1H, t, *J* = 7.7 Hz, aromatics), 5.05–5.00 (2H, m, 2 × OH), 4.95–4.89 (2H, m, H-1 + OH), 4.53 (1H, t, *J* = 5.8 Hz, OH), 3.67 (1H, dd, *J* = 11.5, 5.7 Hz, H-6), 3.46–3.36 (2H, m, H-2 + H-6′), 3.27–3.18 + 3.14–3.06 (3H, m + m, H-3–H-5). ^13^C NMR (100 MHz, CDCl_3_) *δ* (ppm): 158.6 + 158.6 (C=O_CONH_), 141.6, 141.1, 140.6, 134.4, 133.9, 129.3, 128.9, 128.5, 127.6, 126.8, 126.6, 125.1, 124.7, 116.7 (aromatics), 79.6 (C-1), 78.8, 77.4, 72.0, 70.0 (C-2–C-5), 61.1 (C-6). HRMS positive mode *m*/*z*: calculated C_16_H_20_N_3_O_6_^+^ [M+H]^+^ 350.1347, found 350.1346.

#### 5.2.23. *N*-(β-d-Glucopyranosyl)-4(5)-(1-naphthyl)-1*H*-imidazole-2-carboxamide (**2b**)

Prepared from *N*-(2,3,4,6-tetra-*O*-acetyl-β-d-glucopyranosyl)-4(5)-(1-naphthyl)-*1H*-imidazole-2-carboxamide (**11b**) (35 mg, 0.062 mmol) according to general procedure F. Purified using column chromatography (eluent: DCM:MeOH = 5:1) to give 23 mg (92%) of **2b** as a white solid (R_f_ = 0.65, DCM:MeOH = 7:3); [α]_D_ = −6 (c 0.12, MeOH). ^1^H NMR (400 MHz, CDCl_3_) *δ* (ppm): 13.45 (1H, brs, imidazole NH), 8.70 (1H, brs, aromatics), 8.64 (1H, d, *J* = 9.3 Hz, CONH), 7.96 (1H, d, *J* = 7.1 Hz, aromatics), 7.90 (1H, d, *J* = 7.7 Hz, aromatics), 7.77–7.73 (1H, m, aromatics), 7.72 (1H, s, imidazole CH), 7.58–7.52 (3H, m, aromatics), 5.04–5.03 (2H, m, 2 × OH), 4.98–4.93 (2H, m, H-1 and OH), 4.55 (1H, t, *J* = 5.8 Hz, OH), 3.68 (1H, dd, *J* = 12.1, 5.6 Hz, H-6), 3.47–3.37 (2H, m, H-2 and H-6′), 3.28–3.19 and 3.14–3.08 (3H, m, H-3–H-5). ^13^C NMR (100 MHz, CDCl_3_) *δ* (ppm): 158.7 (C=O_CONH_), 140.8, 140.6, 133.6, 132.5, 131.4, 130.6, 128.2, 127.6, 126.5, 126.3, 125.8, 125.5, 119.4 (aromatics), 79.7 (C-1), 78.8, 77.4, 71.9, 70.0 (C-2–C-5), 61.1 (C-6). HRMS positive mode *m*/*z*: calculated C_20_H_22_N_3_O_6_^+^ [M+H]^+^ 400.1503, found 400.1500.

#### 5.2.24. *N*-(β-d-Glucopyranosyl)-4(5)-(2-naphthyl)-1*H*-imidazole-2-carboxamide (**2c**)

Prepared from *N*-(2,3,4,6-tetra-*O*-acetyl-β-d-glucopyranosyl)-4(5)-(2-naphthyl)-*1H*-imidazole-2-carboxamide (**11c**) (23 mg, 0.041 mmol) according to general procedure F. Purified using column chromatography (eluent: DCM:MeOH = 5:1) to give 15 mg (94%) of **2c** as a white solid (R_f_ = 0.60, DCM:MeOH = 7:3); [α]_D_ = +4 (c 0.11, MeOH). ^1^H NMR (400 MHz, CDCl_3_) *δ* (ppm): 13.34 (1H, brs, imidazole NH), 8.60 (1H, d, *J* = 9.5 Hz, CONH), 8.44 (1H, brs, aromatics), 8.06 (1H, d, *J* = 8.4 Hz, aromatics), 7.98 (1H, brs, imidazole CH), 7.94–7.88 (3H, m, aromatics), 7.53–7.45 (2H, m, aromatics), 5.08–5.05 (2H, m, 2 × OH), 4.97–4.92 (2H, m, H-1 and OH), 4.54 (1H, t, *J* = 5.9 Hz, OH), 3.68 (1H, dd, *J* = 12.0, 5.8 Hz, H-6), 3.48–3.39 (2H, m, H-2 and H-6′), 3.29–3.19 and 3.15–3.10 (3H, m and m, H-3–H-5). ^13^C NMR (100 MHz, CDCl_3_) *δ* (ppm): 158.6 (C=O_CONH_), 141.0, 140.9, 133.3, 132.2, 131.5, 128.0, 127.7, 127.7, 126.4, 125.5, 123.8, 122.5, 117.4 (aromatics), 79.6 (C-1), 78.8, 77.4, 72.1, 70.0 (C-2–C-5), 61.1 (C-6). HRMS positive mode *m*/*z*: calculated C_20_H_22_N_3_O_6_^+^ [M+H]^+^ 400.1503, found 400.1503.

## Figures and Tables

**Table 2:** Bound state calculation Glide SP docking results with Glidescores and docking scores (in parentheses) for each ligand tautomer of the target ligands **1a**–**c** and **2a**–**c**. Corresponding results for the previously studied **IX** scaffold [24] are given for comparison.

Ligand	Tautomer ^a^	Phenyl (**a**)	1-Naphthyl (**b**)	2-Naphthyl (**c**)
**IX**	**t1**	−9.52 (−9.42)	−9.77 (−9.69)	−10.80 (−10.71)
	**t2**	−9.49 (−7.99)	−9.92 (−8.42)	−10.97 (−9.47)
	**t3**	−9.33 (−7.51)	−9.79 (−7.97)	−10.67 (−8.85)
				
**1**	**t1**	−9.81 (−9.17)	−10.27 (−9.63)	−10.97 (−10.33)
	**t2**	−9.55 (−9.30)	−9.89 (−9.64)	−10.97 (−10.72)
				
**2**	**t1**	−9.70 (−9.29)	−9.93 (−9.53)	−11.00 (−10.59)
	**t2**	−9.61 (−9.18)	−9.99 (−9.56)	−10.99 (−10.55)

^a^ *c.f.* Figure 1 for tautomeric state chemical structures.

**Table 3:** Synthesis of 2-arylimidazole-4(5)-carboxylic acids.

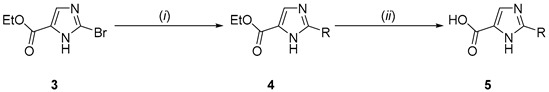 (*i*) R-B(OH)_2_/[Pd]/dry TEA/dry 1,4-dioxane/reflux/Ar; (*ii*) LiOH/EtOH-H_2_O/50 °C				
			Yields (%)	
Entry		R	**4**	**5**
1	**a**	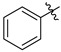	31	68
2	**b**	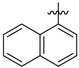	25	63
3	**c**	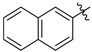	19	61

**Table 4:** Synthesis of 4(5)-arylimidazole-2-carboxylic acids.

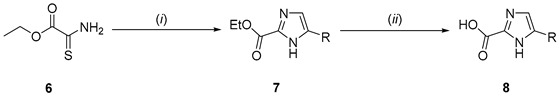 (*i*) Et_3_OBF_4_/dry DCM, then 2-amino-1-arylethan-1-one/NaOAc/AcOH/70 °C; (*ii*) LiOH/EtOH-H_2_O/50 °C				
			Yields (%)	
Entry		R	**7**	**8**
1	**a**	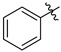	57	71
2	**b**	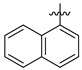	29	75
3	**c**	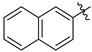	75	82

**Table 5:** Inhibition constants (*K*_i_s in µM) for the synthesised *N*-(β-d-glucopyranosyl) imidazolecarboxamides ^a^.

Compound	Ar		
	** 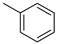 **	** 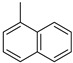 **	** 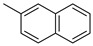 **
	**a**	**b**	**c**
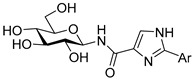	10.4 ± 6.2	16.2 ± 8.1	12.8 ± 3.0
**1**			
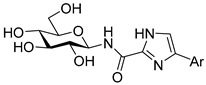	67.4 ± 9.7	4.1 ± 0.8	3.3 ± 0.1
**2**			

^a^ All *K*_i_ calculations were carried out using the GraphPad Prism5 (GraphPad Software, La Jolla, CA, USA) software. The results of kinetic experiments were calculated from at least three independent experiments. The standard deviations were calculated based on the average values for the independent experiments.

## Data Availability

Not applicable.

## Supplementary Materials

The following supporting information can be downloaded at: https://www.mdpi.com/article/10.3390/ijms25094591/s1.
