# Supplementary material for: Synthesis, In Silico and Kinetics Evaluation of N-(β-d-glucopyranosyl)-2-arylimidazole-4(5)-carboxamides and N-(β-d-glucopyranosyl)-4(5)-arylimidazole-2-carboxamides as Glycogen Phosphorylase Inhibitors
Source: Int J Mol Sci. 2024 Apr 23;25(9):4591. doi: 10.3390/ijms25094591 (PMC11083775; doi:10.3390/ijms25094591)
Supplement: Supplementary file 1 [file ijms-25-04591-s001.zip › ijms-2935269-supplementary.pdf]

Synthesis, *in silico* and kinetics evaluation of *N*-(β-D-glucopyranosyl)-2-arylimidazole-4(5)-carboxamides and *N*-(β-D-glucopyranosyl)-4(5)-arylimidazole-2-carboxamides as glycogen phosphorylase inhibitors

Levente Homolya,[a] Rachel T. Mathomes,[b] Luca Varga,[c] Tibor Docsa,[c] László Juhász,*[a] Joseph M. Hayes,*[b] and László Somsák*[a]

[a] *Department of Organic Chemistry, University of Debrecen, POB 400, H-4002 Debrecen, Hungary*
[b] *School of Pharmacy & Biomedical Sciences, University of Central Lancashire, Preston PR1 2HE, United Kingdom*
[c] *Department of Medical Chemistry, University of Debrecen, Faculty of Medicine, University of Debrecen, 4032 Debrecen, Hungary*

Table of Contents

Ethyl 2-phenyl-1*H*-imidazole-4(5)-carboxylate (4a) 3
Ethyl 2-(1-naphthyl)-1*H*-imidazole-4(5)-carboxylate (4b) 5
Ethyl 2-(2-naphthyl)-1*H*-imidazole-4(5)-carboxylate (4c) 7
2-Phenyl-1*H*-imidazole-4(5)-carboxylic acid (5a) 9
2-(1-Naphthyl)-1*H*-imidazole-4(5)-carboxylic acid (5b) 11
2-(2-Naphthyl)-1*H*-imidazole-4(5)-carboxylic acid (5c) 13
Ethyl 4(5)-phenyl-1*H*-imidazole-2-carboxylate (7a) 15
Ethyl 4(5)-(1-naphthyl)-1*H*-imidazole-2-carboxylate (7b) 16
Ethyl 4(5)-(2-naphthyl)-1*H*-imidazole-2-carboxylate (7c) 18
4(5)-Phenyl-1*H*-imidazole-2-carboxylic acid (8a) 20
4(5)-(1-Naphthyl)-1*H*-imidazole-2-carboxylic acid (8b) 21
4(5)-(2-Naphthyl)-1*H*-imidazole-2-carboxylic acid (8c) 23
***N*-(2,3,4,6-Tetra-*O*-acetyl-β-D-glucopyranosyl)-2-phenyl-1*H*-imidazole-4(5)-carboxamide (10a)** 25
***N*-(2,3,4,6-Tetra-*O*-acetyl-β-D-glucopyranosyl)-2-(1-naphthyl)-1*H*-imidazole-4(5)-carboxamide (10b)** 28
***N*-(2,3,4,6-Tetra-*O*-acetyl-β-D-glucopyranosyl)-2-(2-naphthyl)-1*H*-imidazole-4(5)-carboxamide (10c)** 31

***N*-(2,3,4,6-Tetra-*O*-acetyl-β-D-glucopyranosyl)-4(5)-phenyl-1*H*-imidazole-2-carboxamide (11a)** 34

***N*-(2,3,4,6-Tetra-*O*-acetyl-β-D-glucopyranosyl)-4(5)-(1-naphthyl)-1*H*-imidazole-2-carboxamide (11b)** 37

***N*-(2,3,4,6-Tetra-*O*-acetyl-β-D-glucopyranosyl)-4(5)-(2-naphthyl)-1*H*-imidazole-2-carboxamide (11c)** 40

***N*-(β-D-Glucopyranosyl)-2-phenyl-1*H*-imidazole-4(5)-carboxamide (1a)** 43

***N*-(β-D-Glucopyranosyl)-2-(1-naphthyl)-1*H*-imidazole-4(5)-carboxamide (1b)** 46

***N*-(β-D-Glucopyranosyl)-2-(2-naphthyl)-1*H*-imidazole-4(5)-carboxamide (1c)** 49

***N*-(β-D-Glucopyranosyl)-4(5)-phenyl-1*H*-imidazole-2-carboxamide (2a)** 52

***N*-(β-D-Glucopyranosyl)-4(5)-(1-naphthyl)-1*H*-imidazole-2-carboxamide (2b)** 55

***N*-(β-D-Glucopyranosyl)-4(5)-(2-naphthyl)-1*H*-imidazole-2-carboxamide (2c)** 58

Ethyl 2-phenyl-1*H*-imidazole-4(5)-carboxylate (4a)

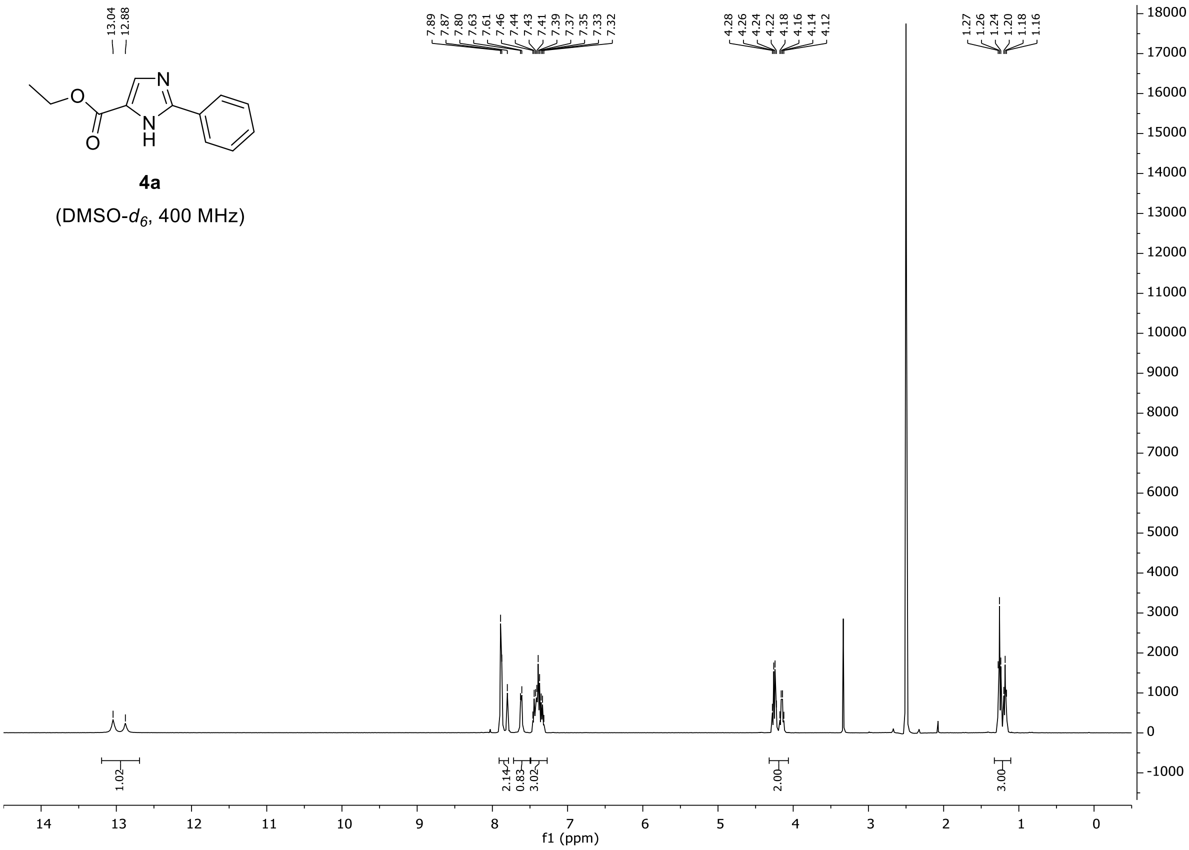

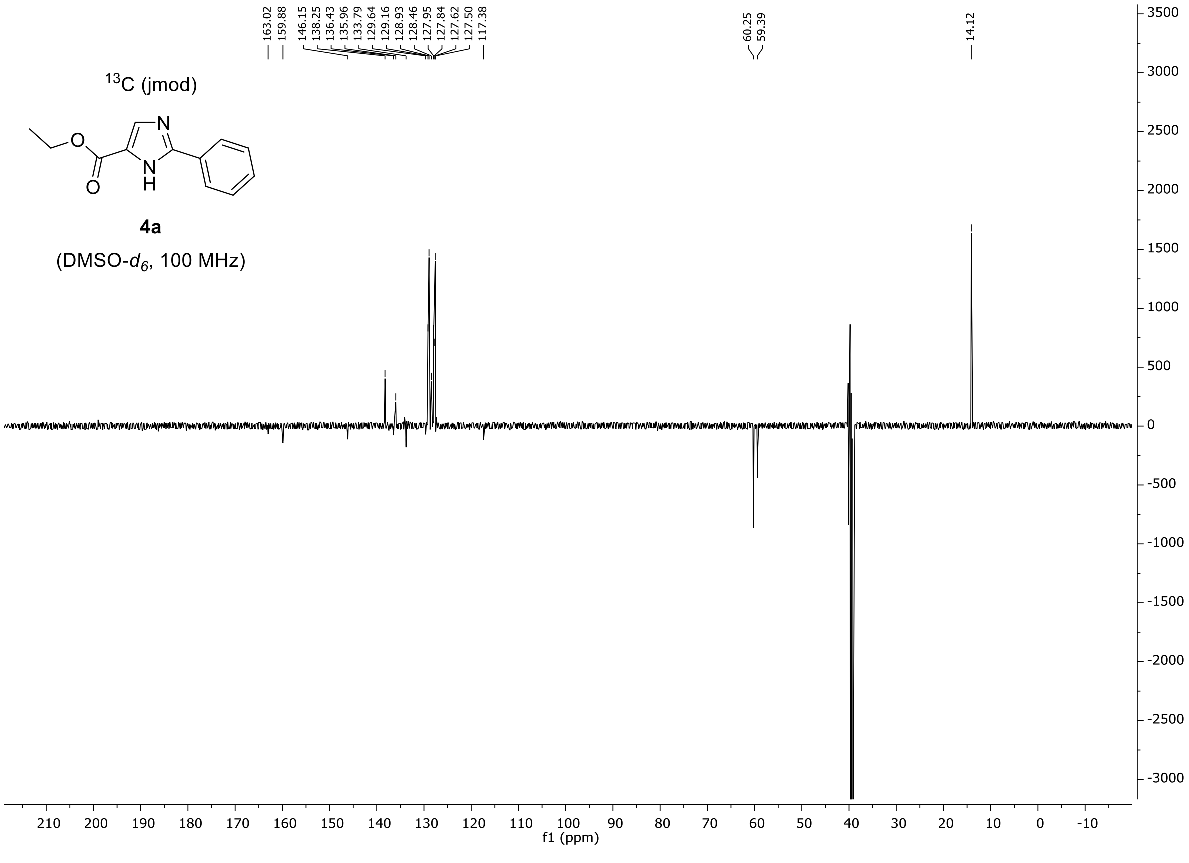

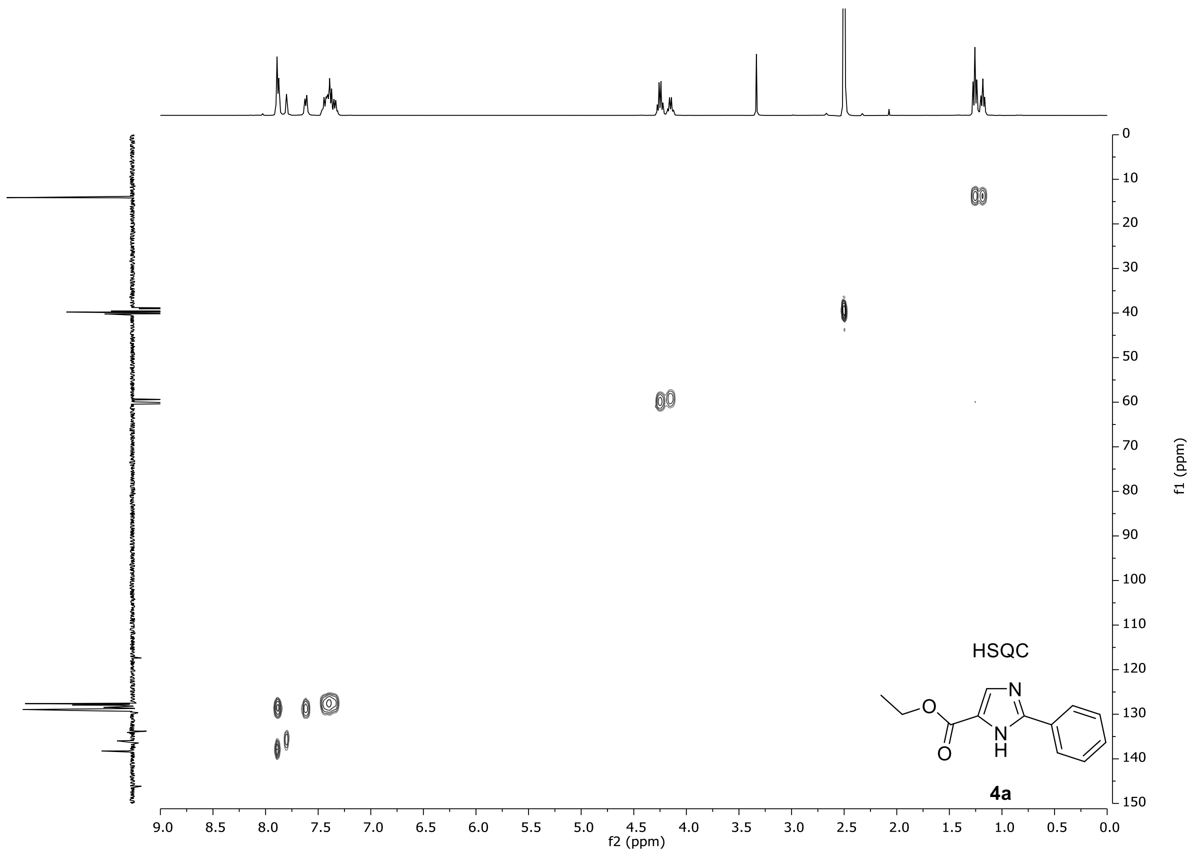
HSQC
4a
f2 (ppm)
f1 (ppm)

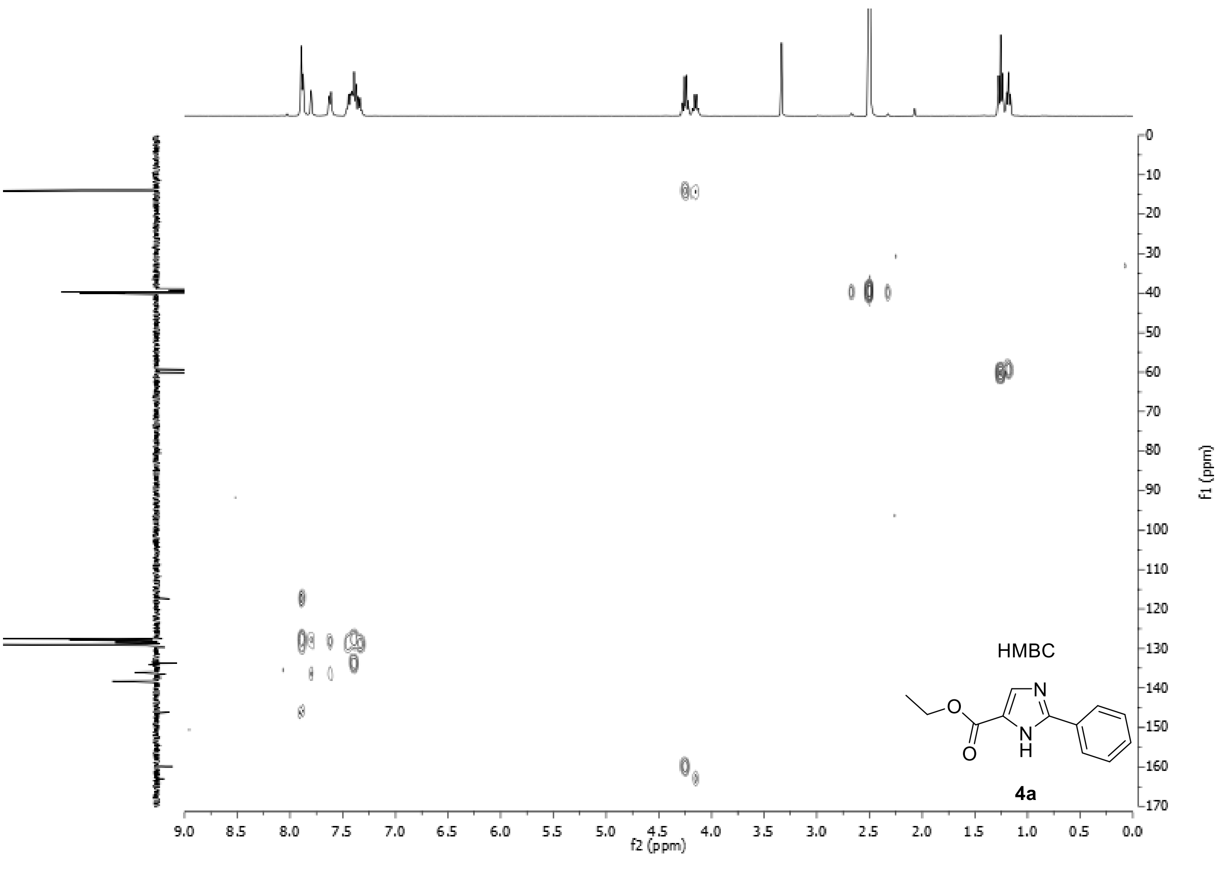
HMBC
4a
f2 (ppm)
f1 (ppm)

Ethyl 2-(1-naphthyl)-1*H*-imidazole-4(5)-carboxylate (4b)

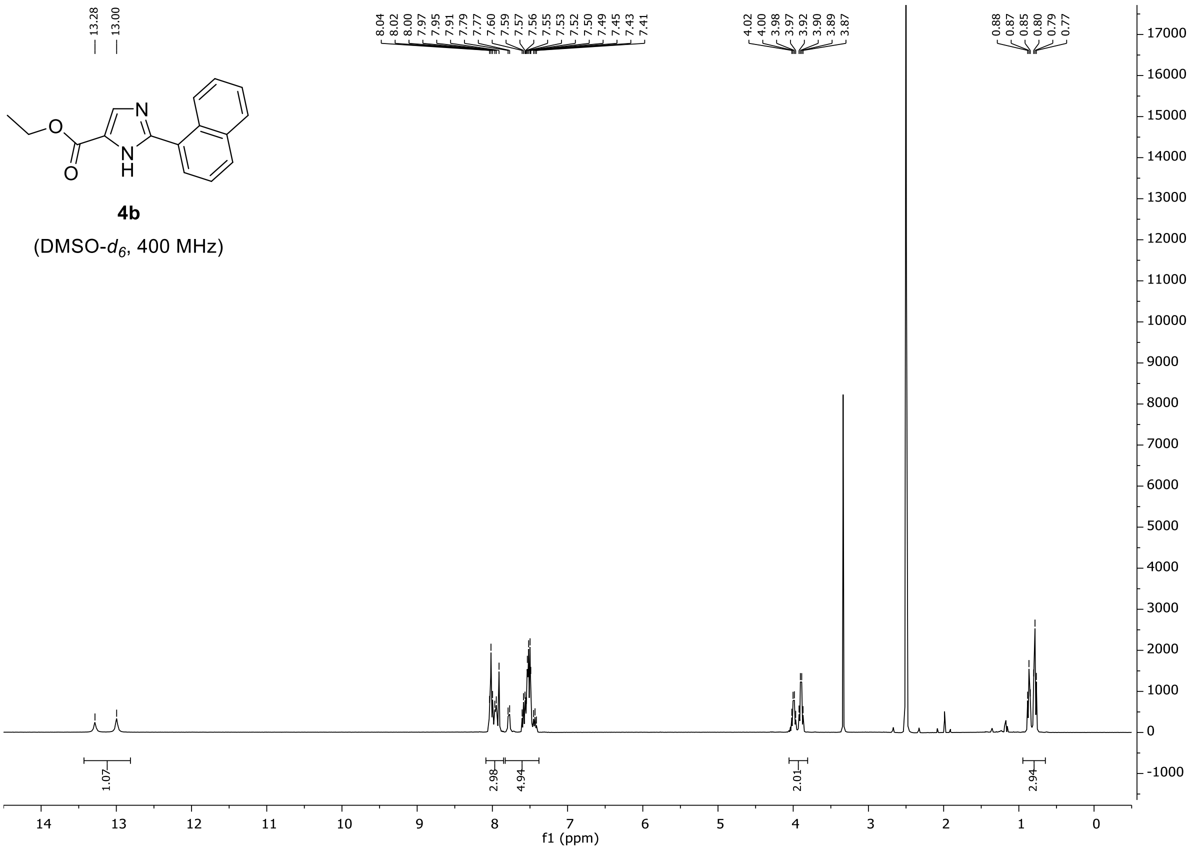

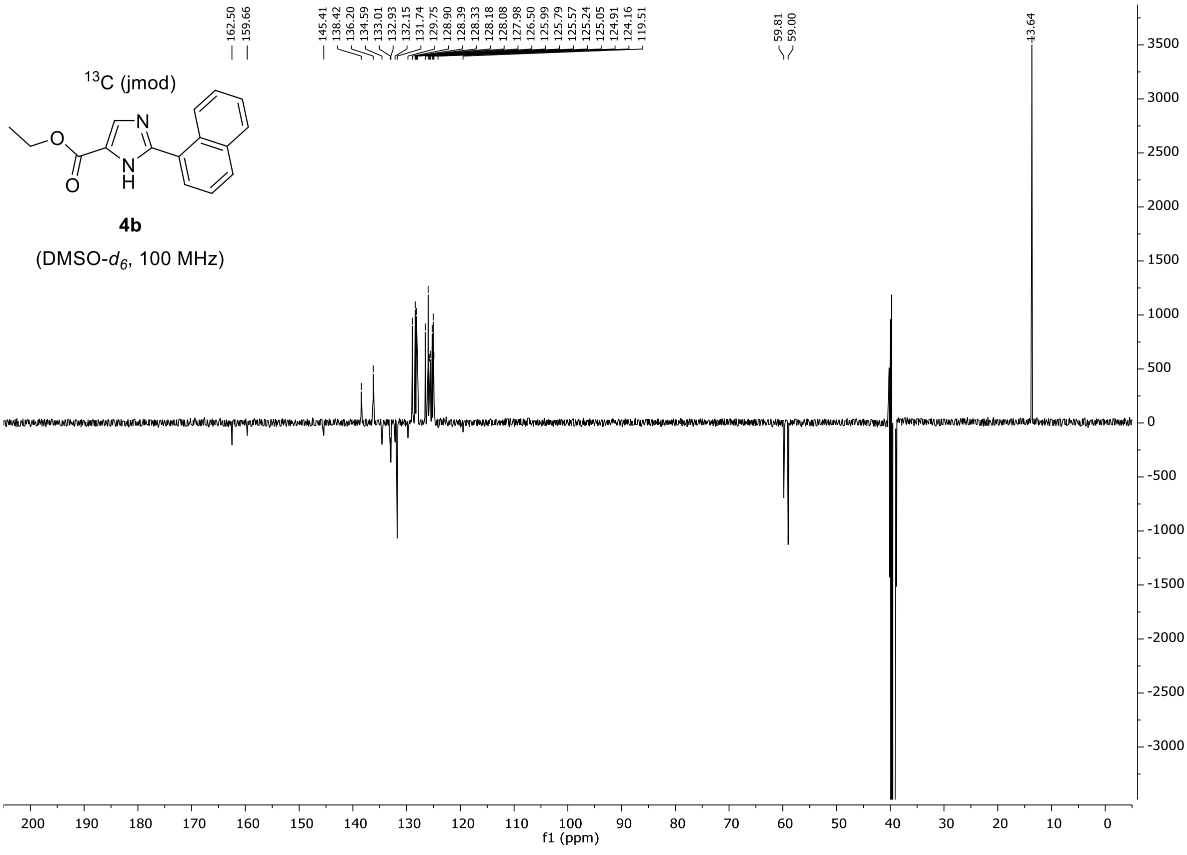

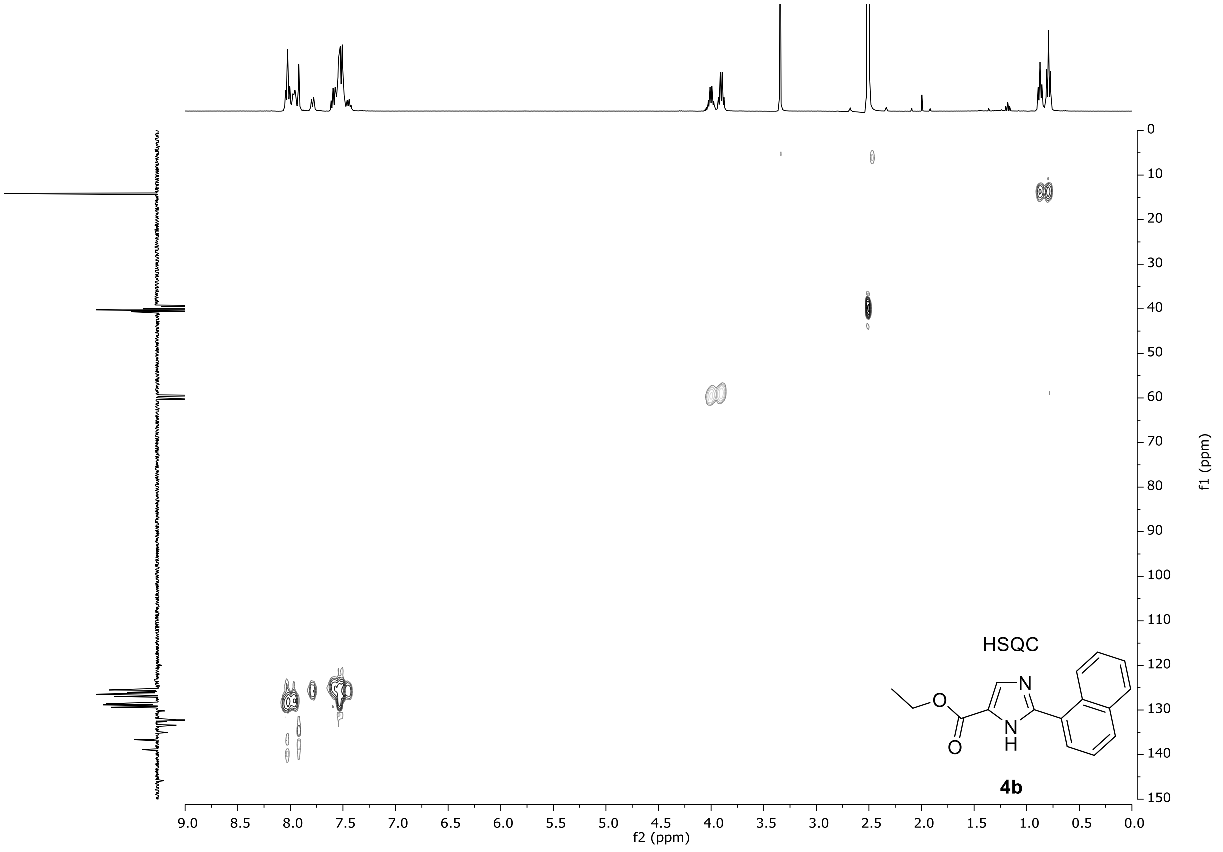
HSQC
4b
f2 (ppm)
f1 (ppm)

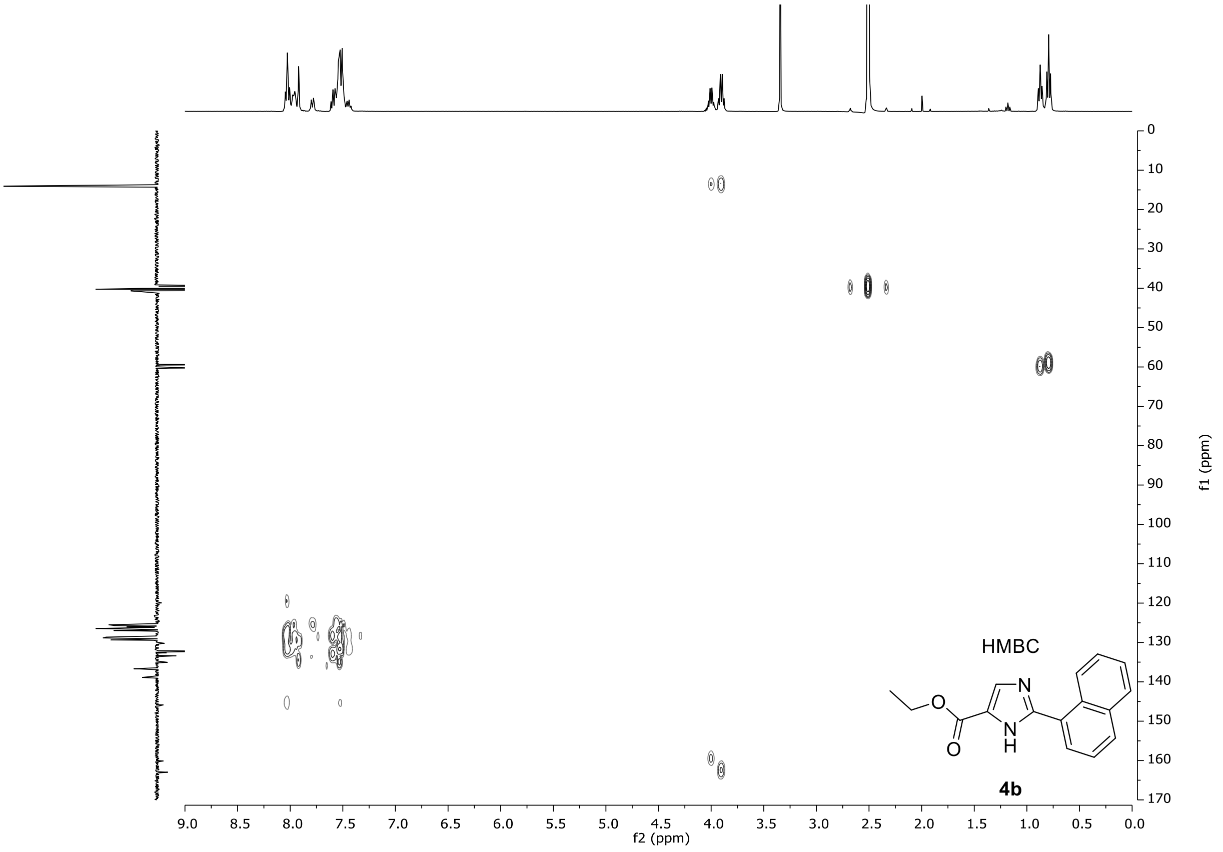
HMBC
4b
f2 (ppm)
f1 (ppm)

Ethyl 2-(2-naphthyl)-1*H*-imidazole-4(5)-carboxylate (4c)

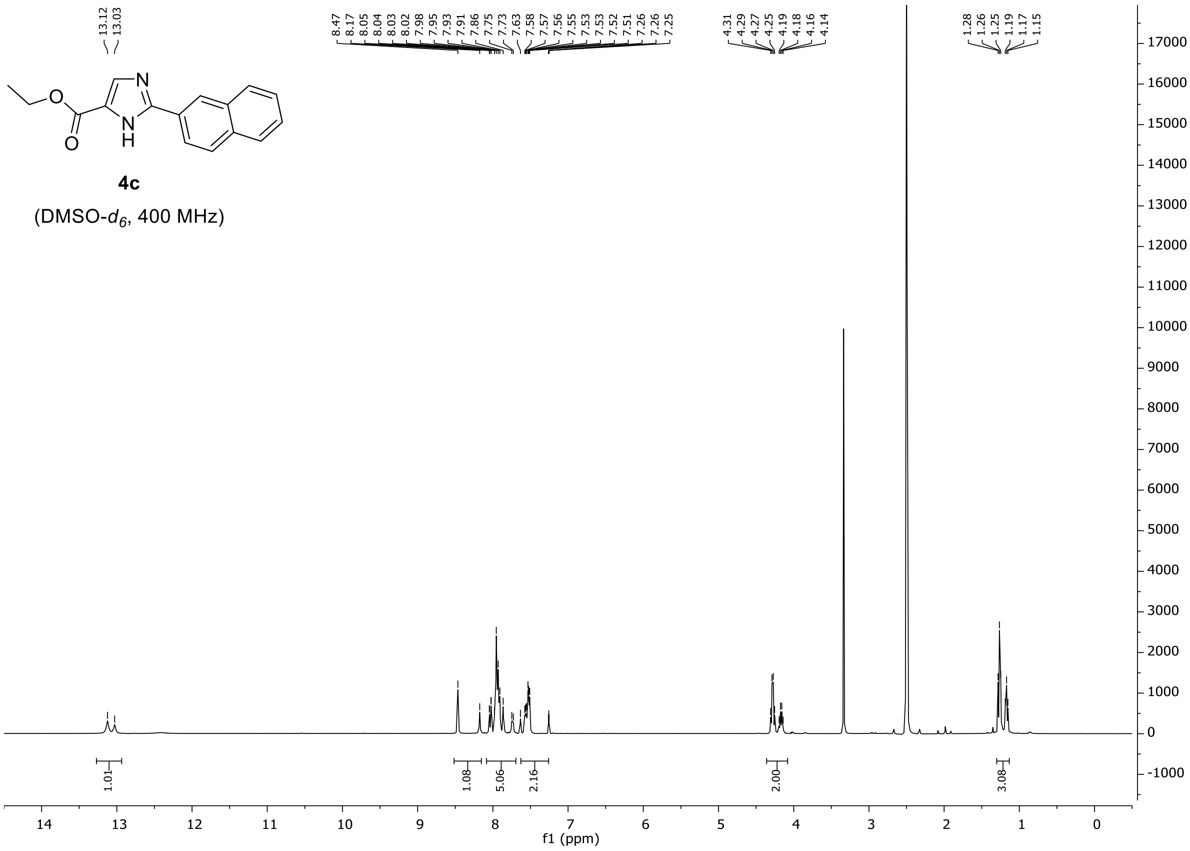

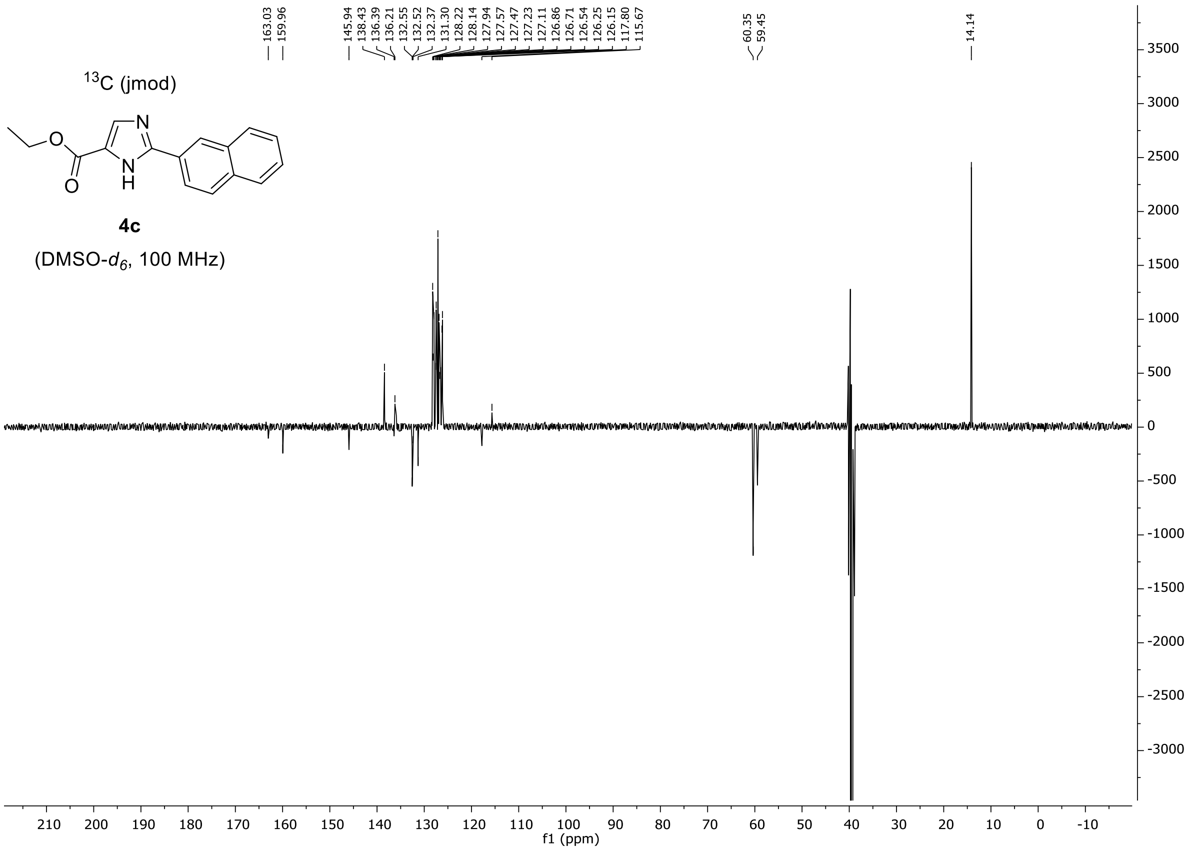

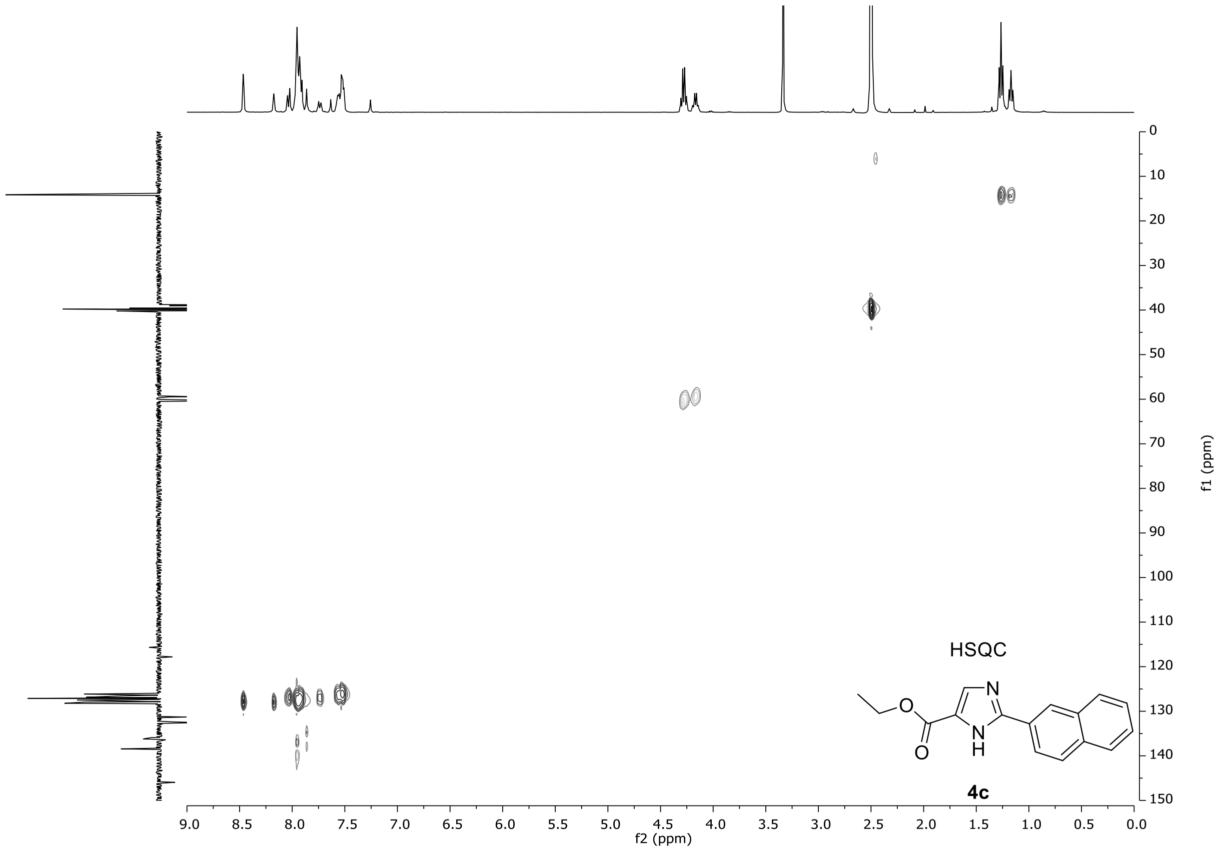
HSQC
4c
f1 (ppm)
f2 (ppm)

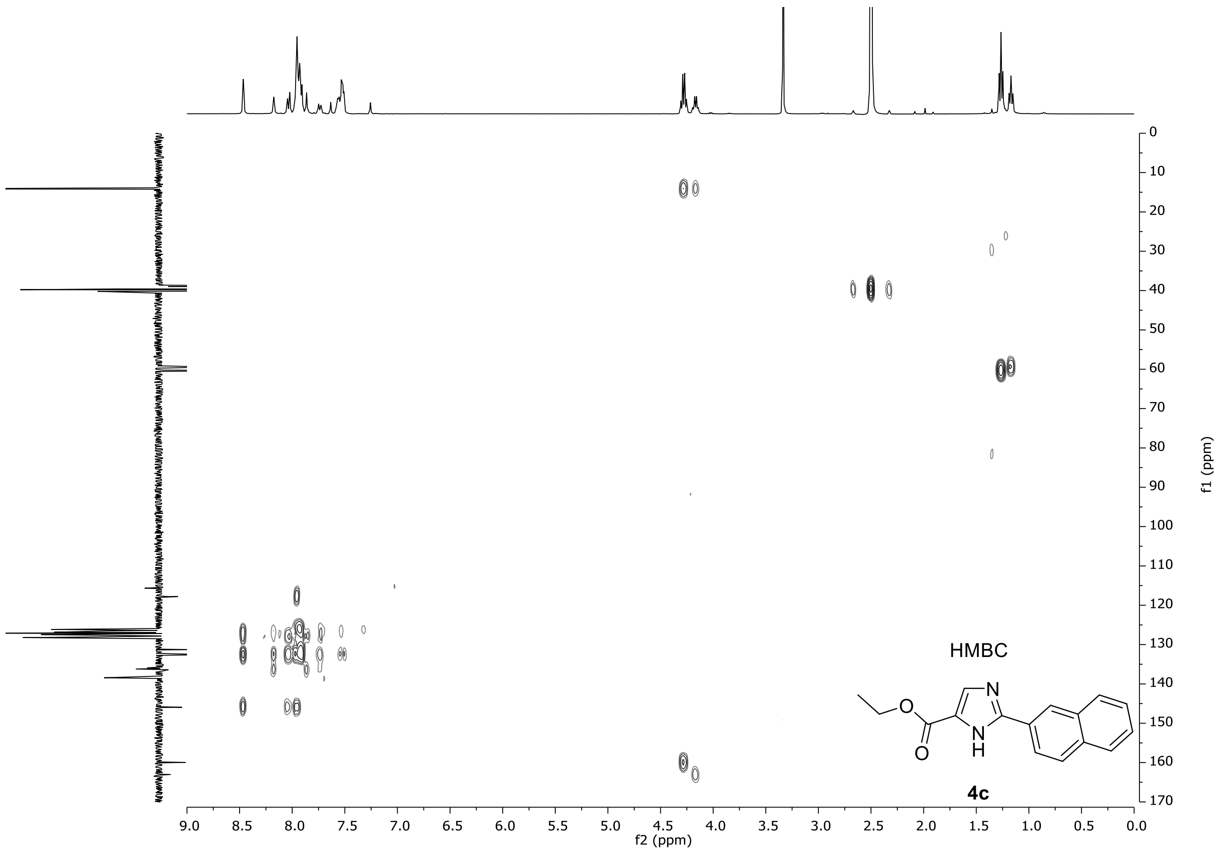
HMBC
4c
f1 (ppm)
f2 (ppm)

2-Phenyl-1*H*-imidazole-4(5)-carboxylic acid (5a)

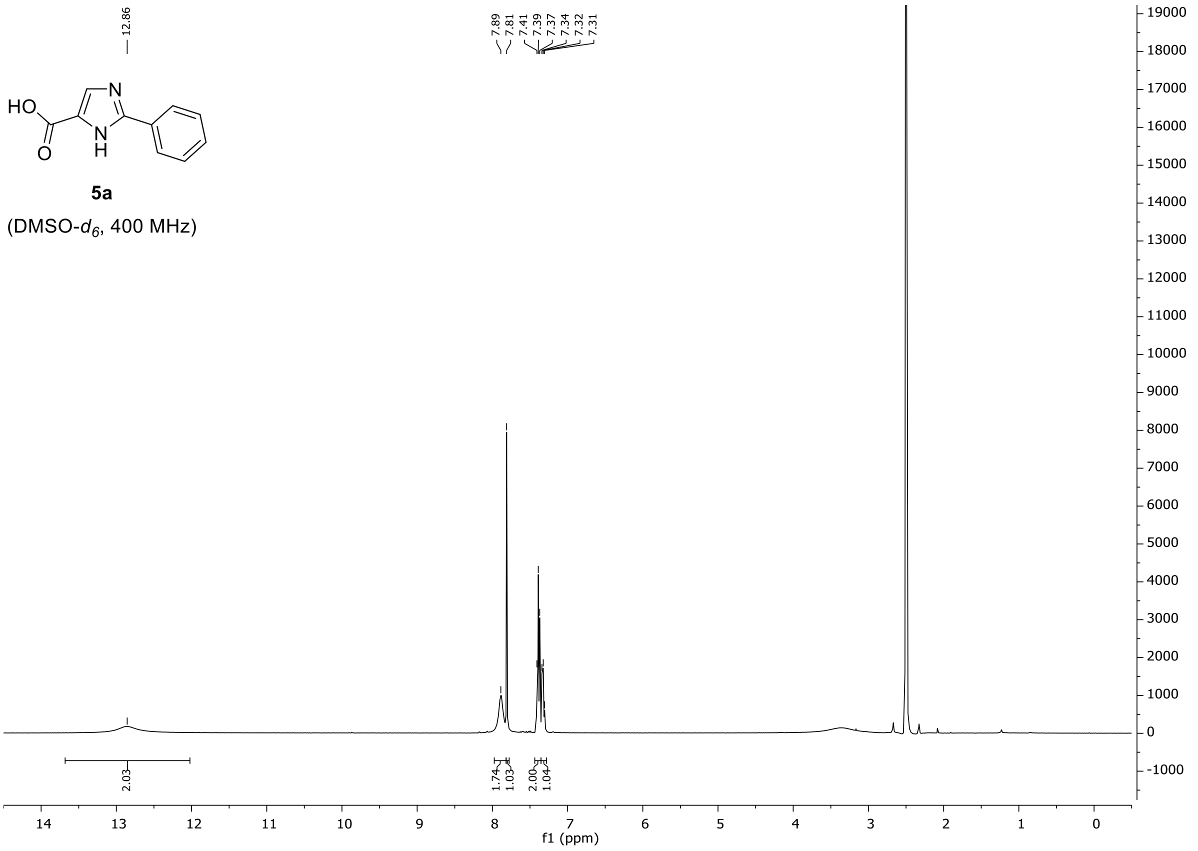

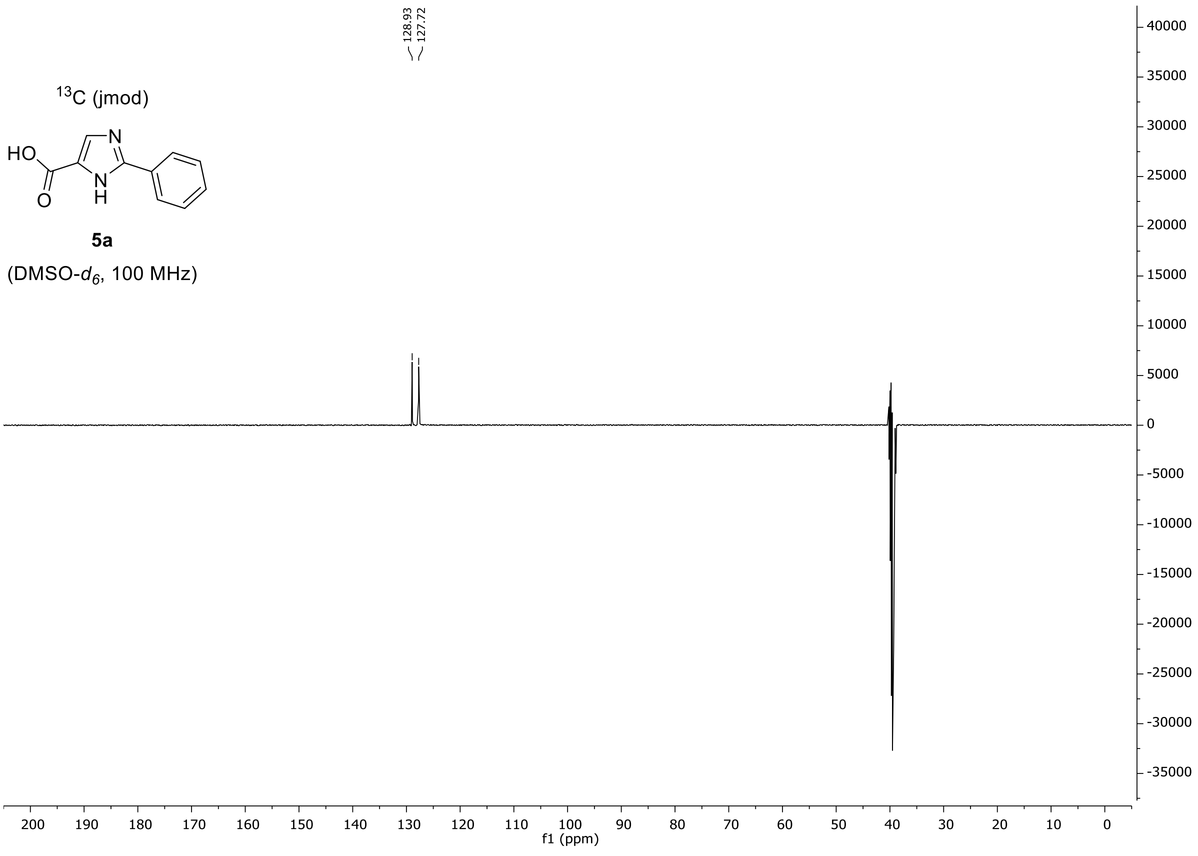

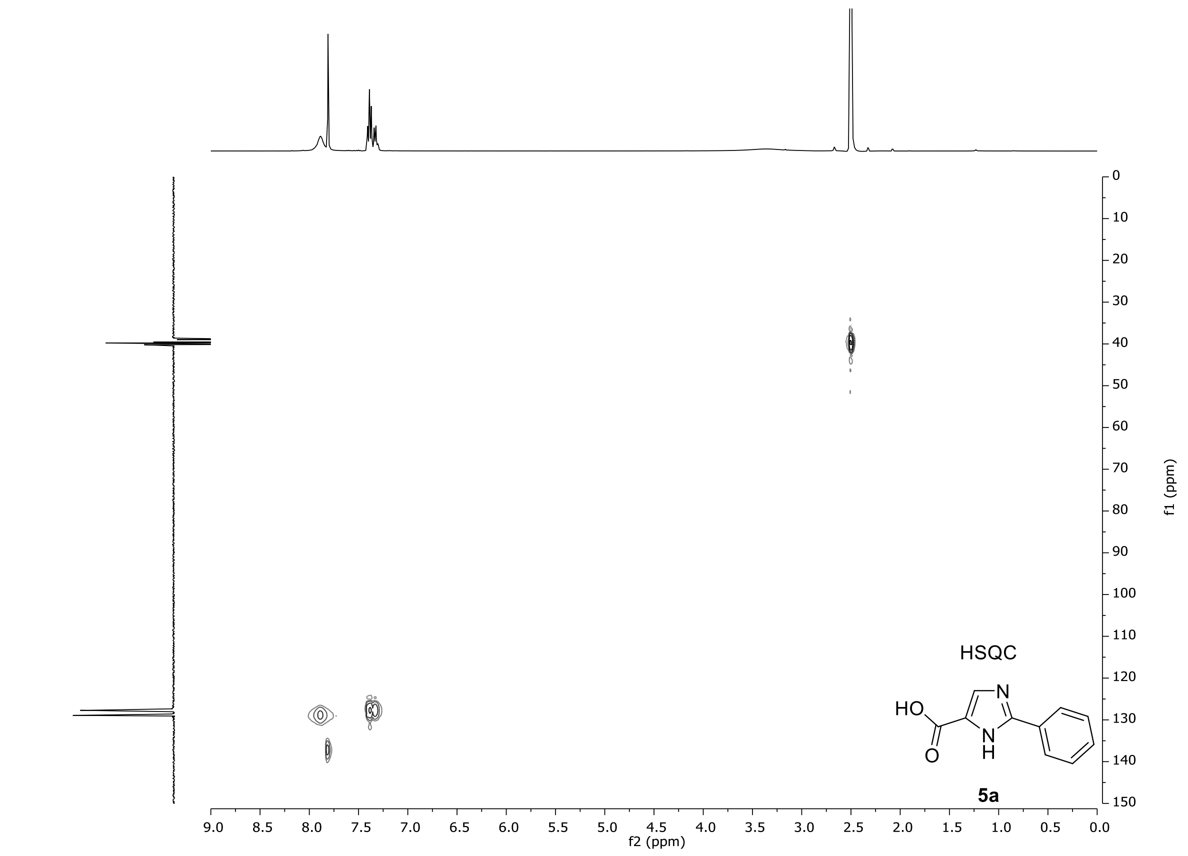
HSQC
HO
N
N
H
O
5a
f1 (ppm)
f2 (ppm)

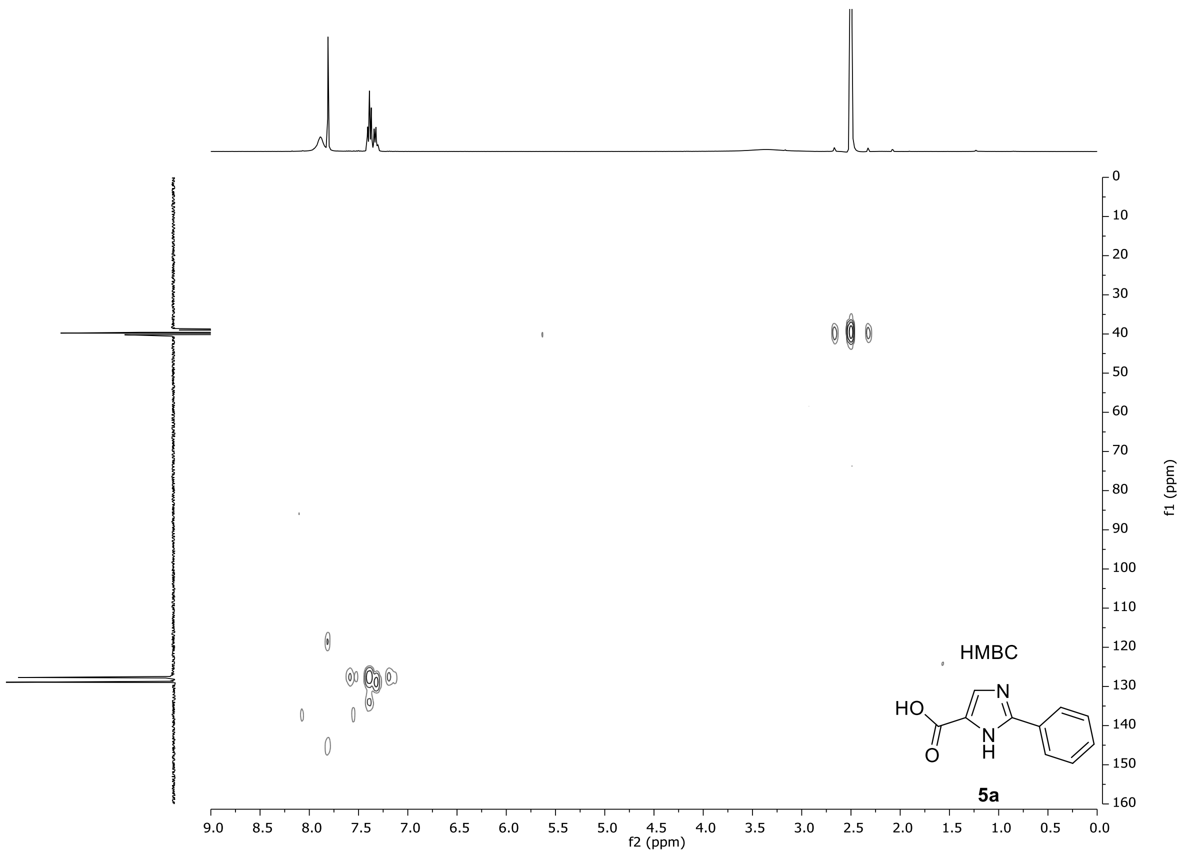
HMBC
HO
N
N
H
O
5a
f1 (ppm)
f2 (ppm)

2-(1-Naphthyl)-1*H*-imidazole-4(5)-carboxylic acid (5b)

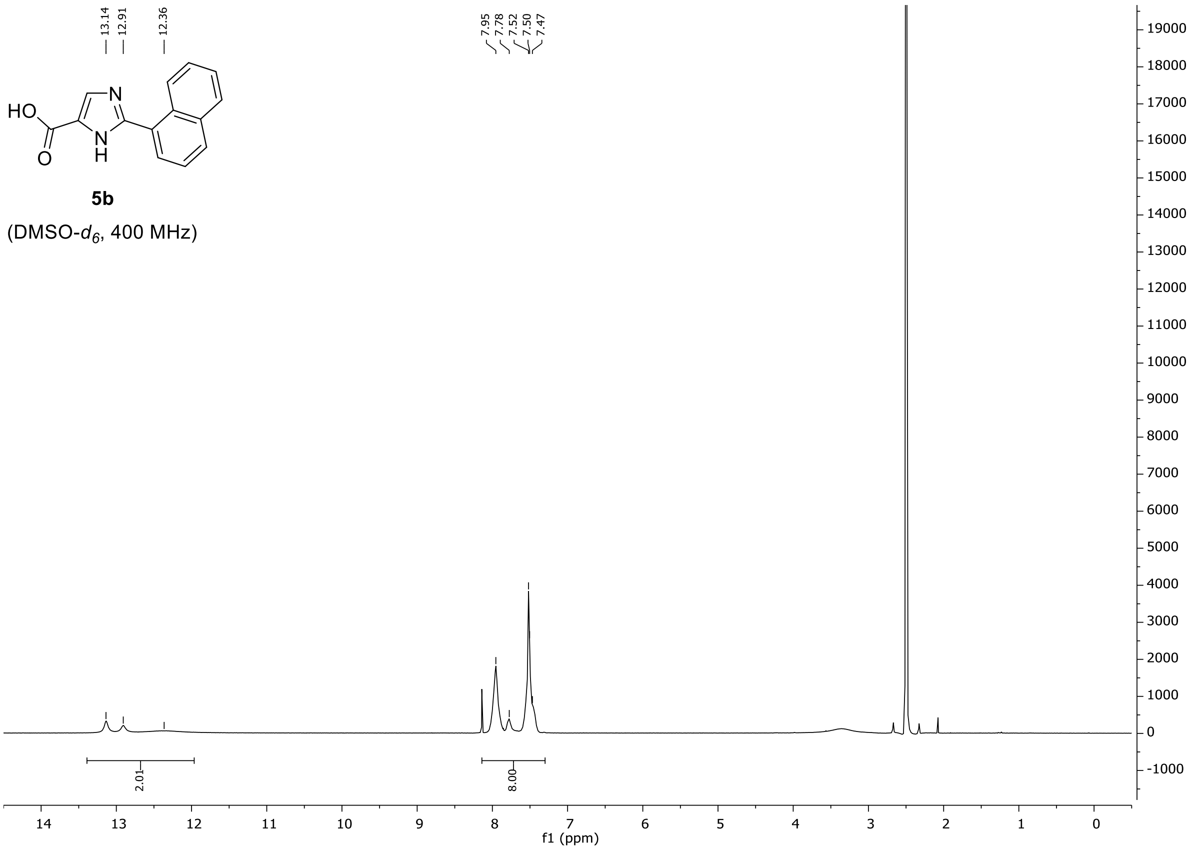

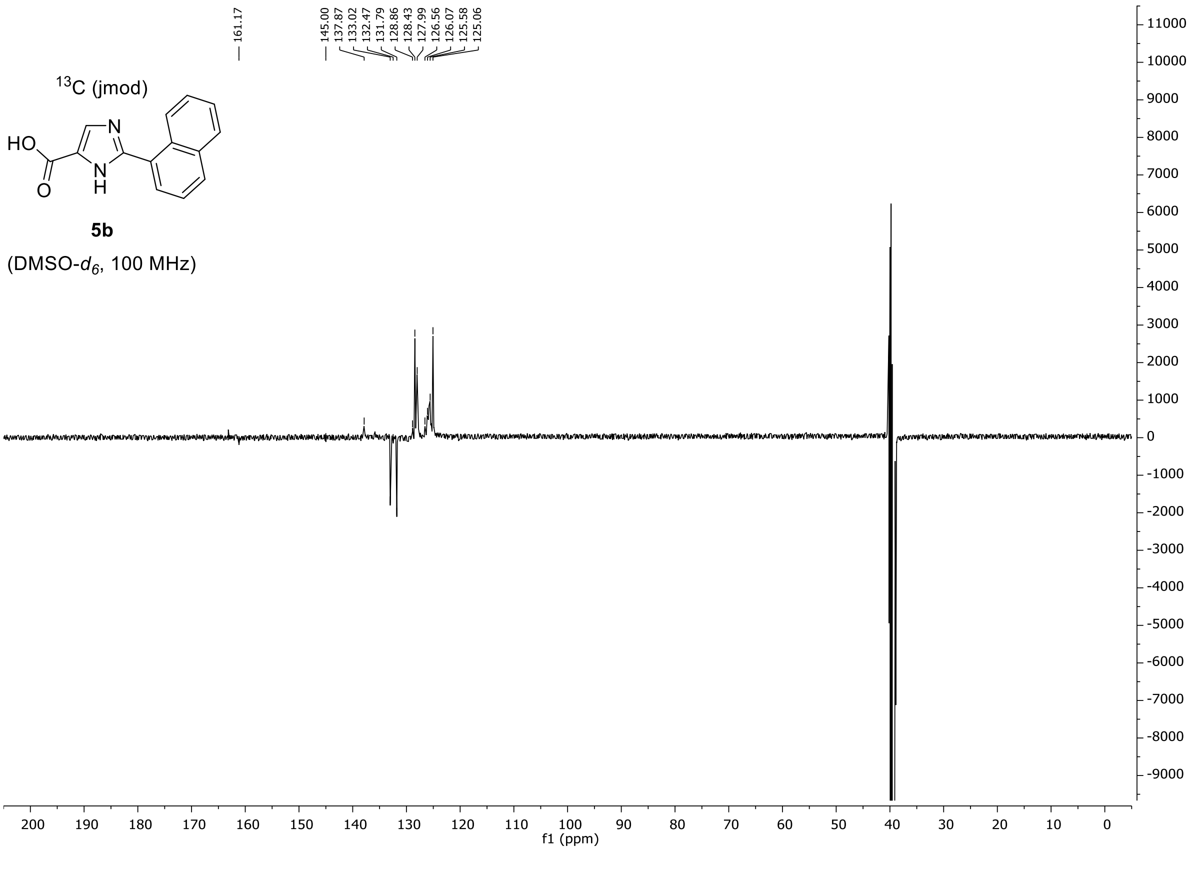

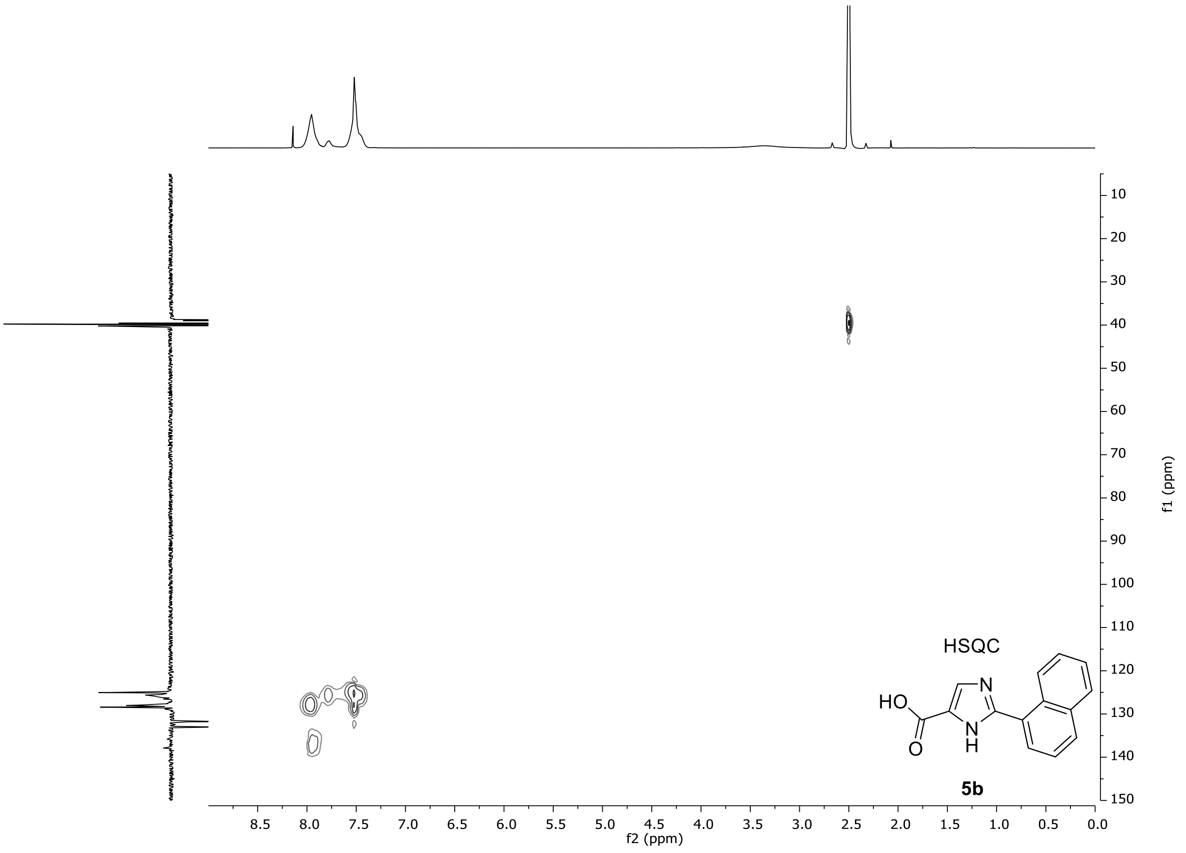
HSQC
HO
N
H
N
O
5b
f1 (ppm)
f2 (ppm)

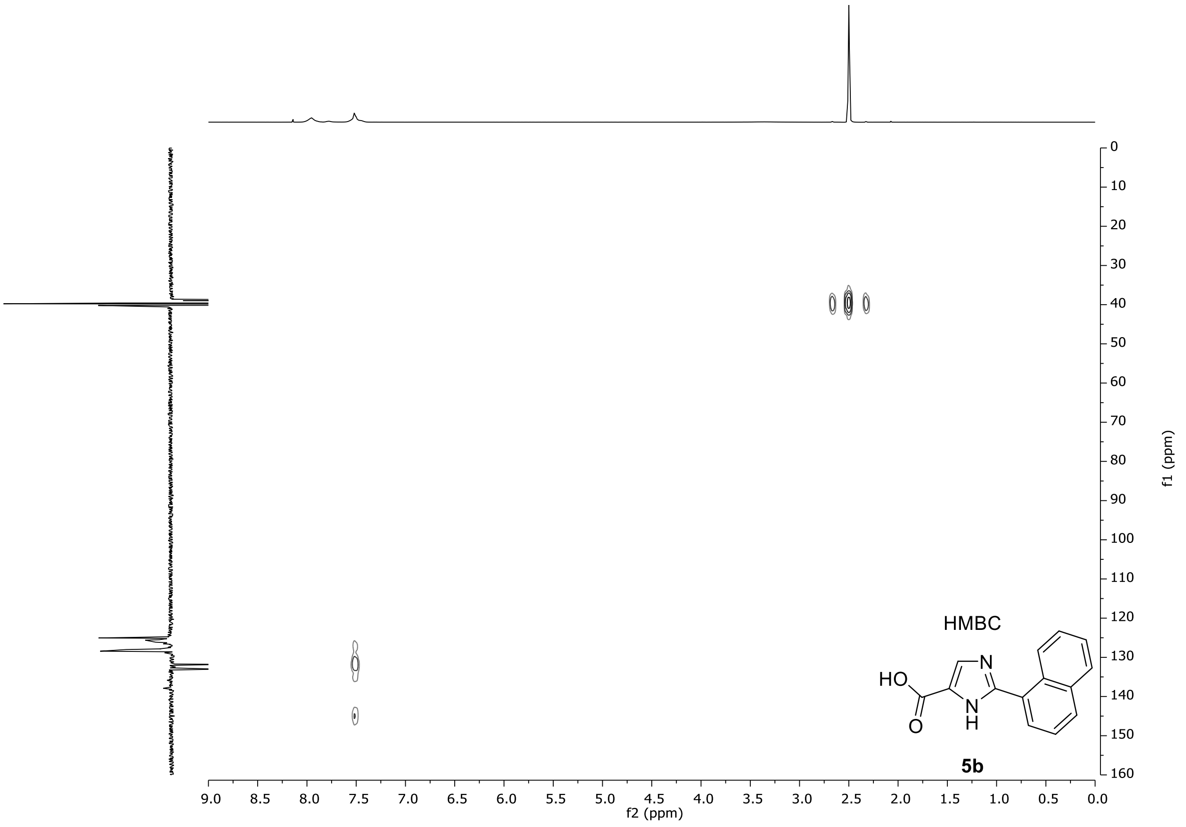
HMBC
HO
N
H
N
O
5b
f1 (ppm)
f2 (ppm)

2-(2-Naphthyl)-1*H*-imidazole-4(5)-carboxylic acid (5c)

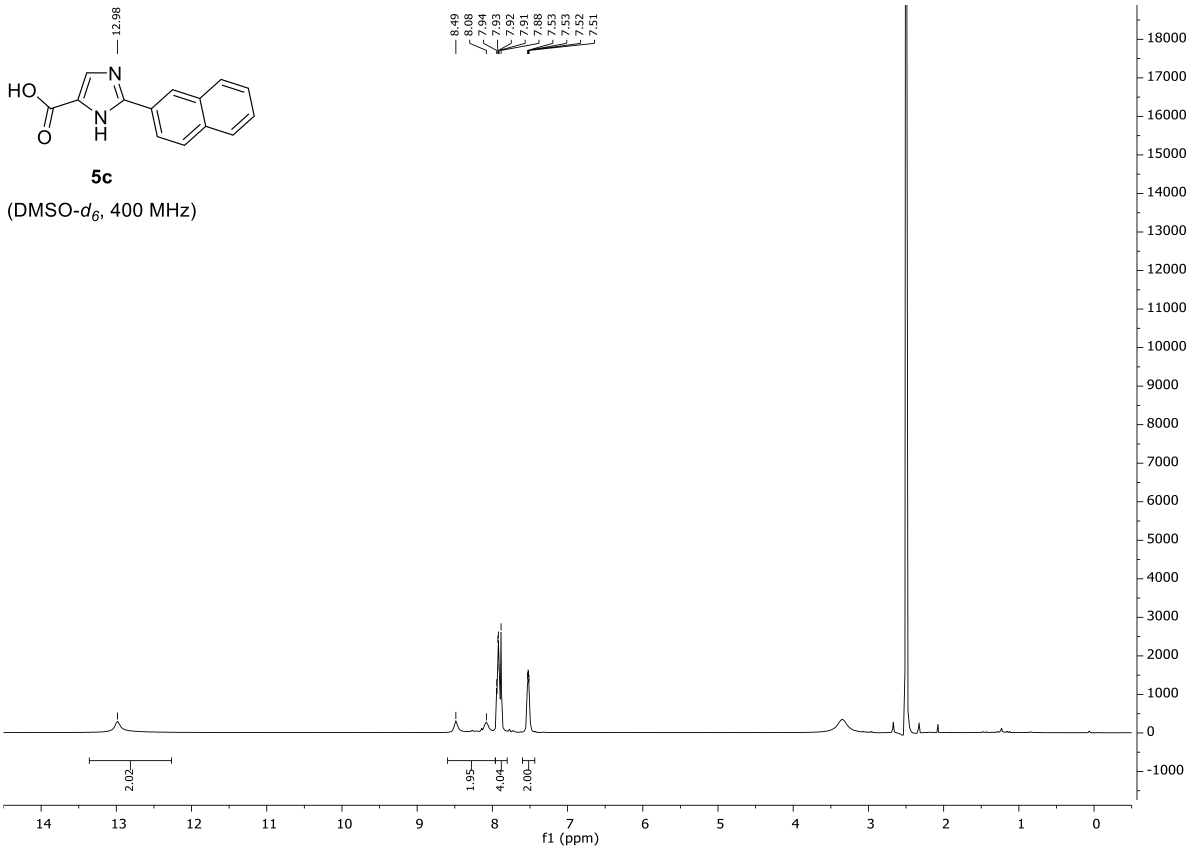

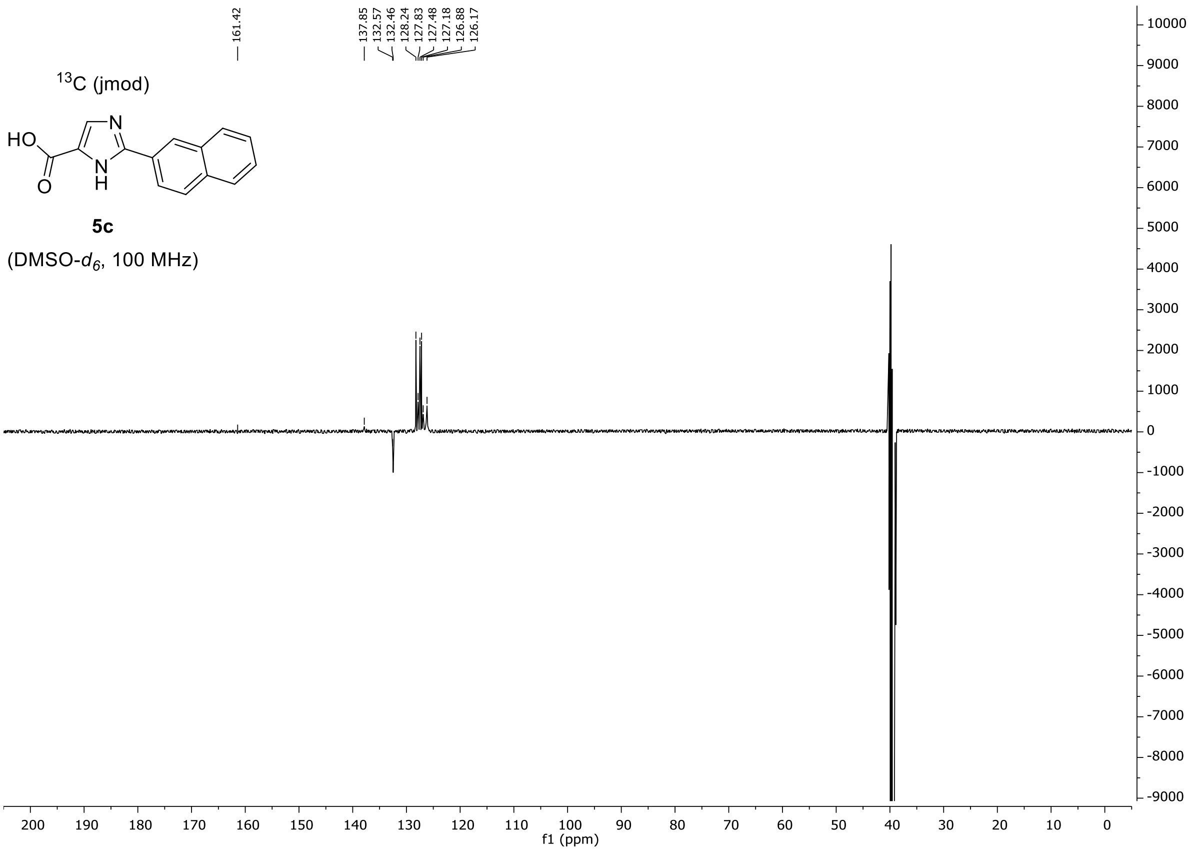

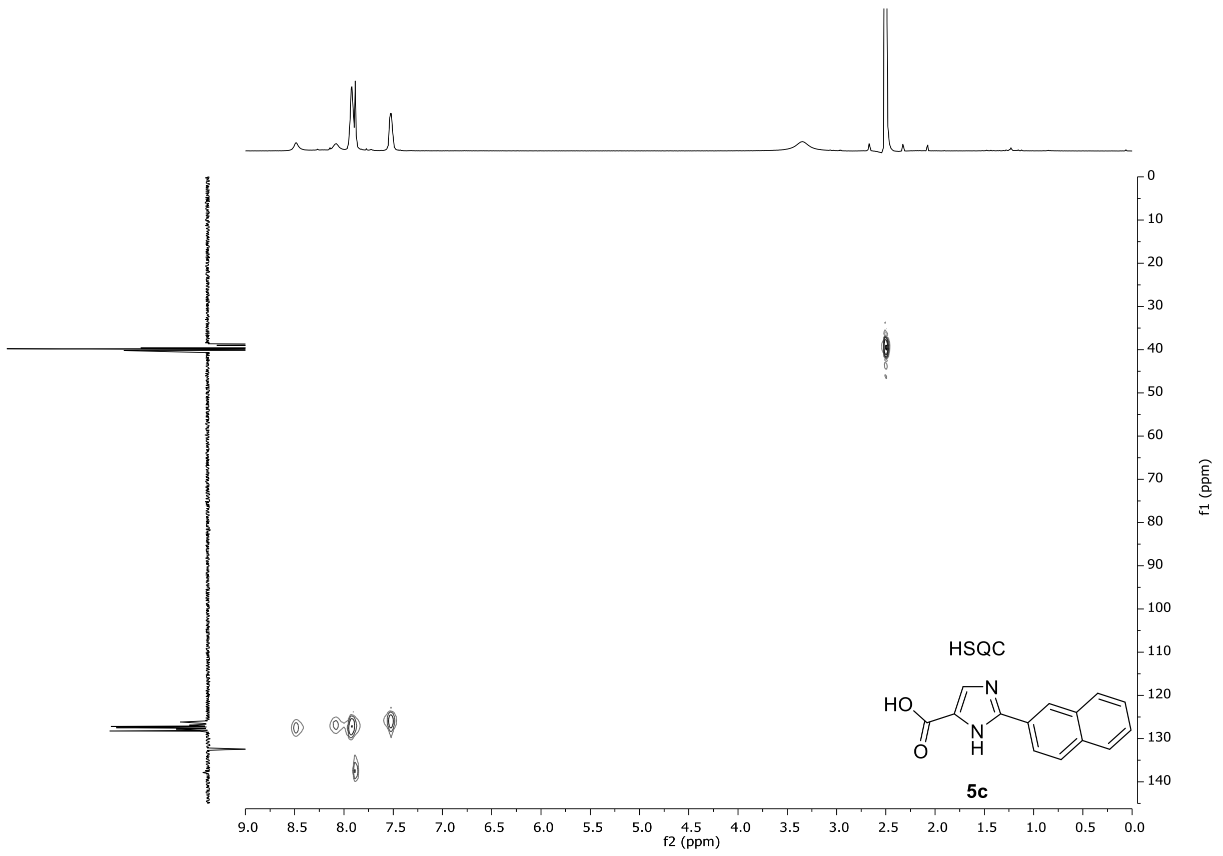
HSQC
HO
N
N
H
O
5c
f1 (ppm)
f2 (ppm)

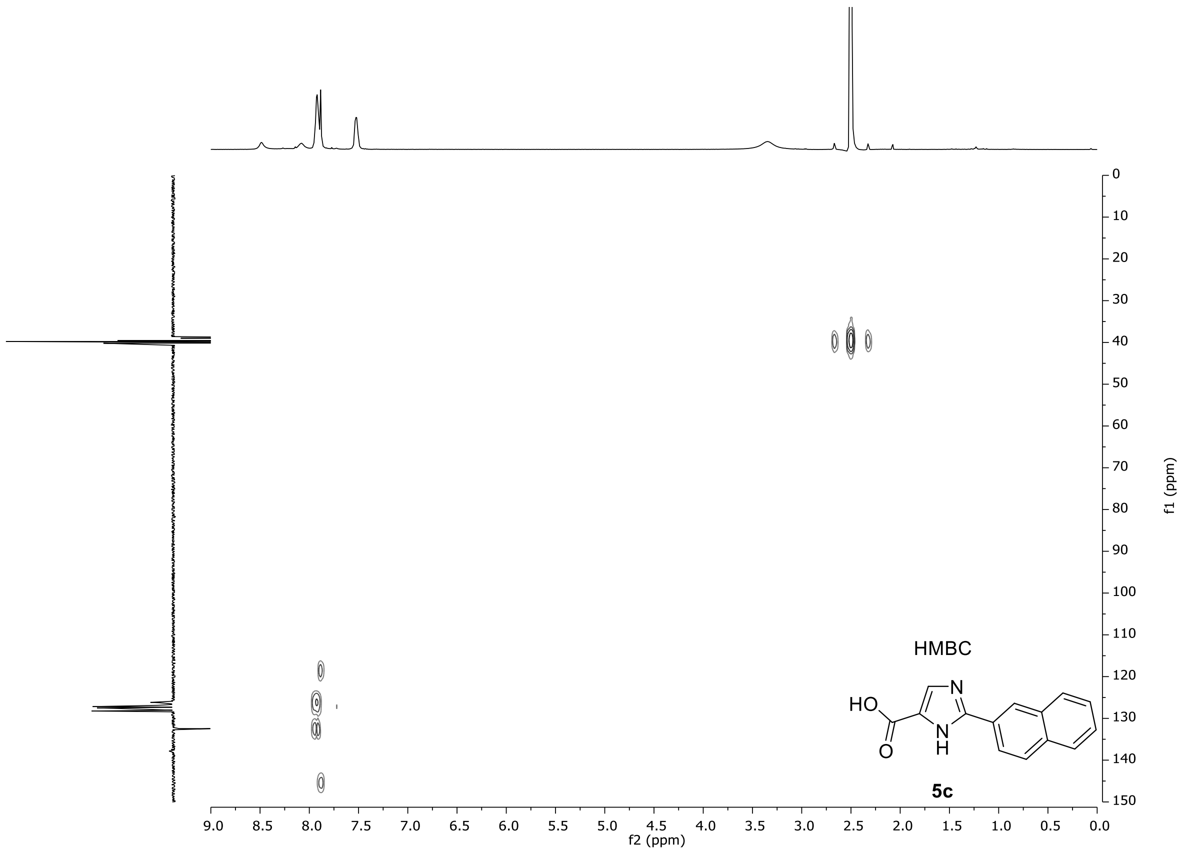
HMBC
HO
N
N
H
O
5c
f1 (ppm)
f2 (ppm)

Ethyl 4(5)-phenyl-1*H*-imidazole-2-carboxylate (7a)

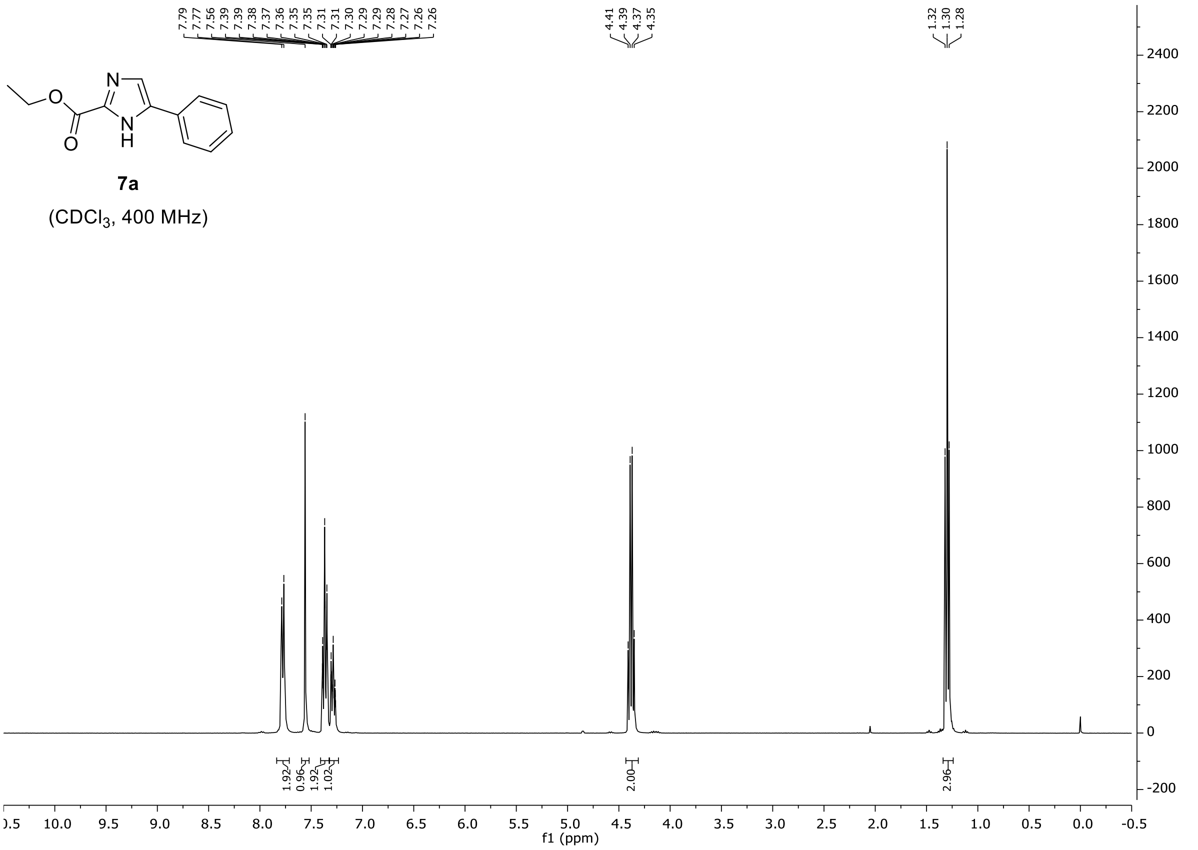

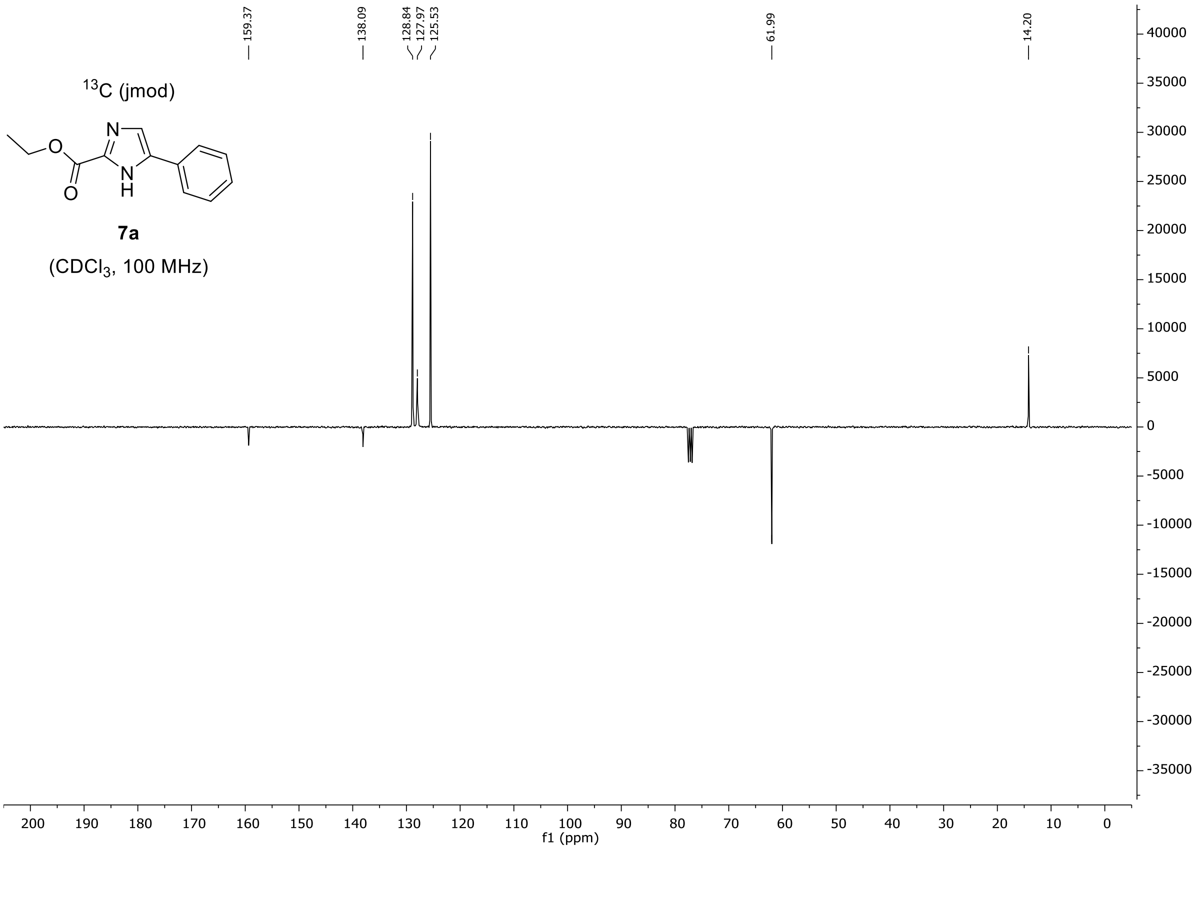

Ethyl 4(5)-(1-naphthyl)-1*H*-imidazole-2-carboxylate (7b)

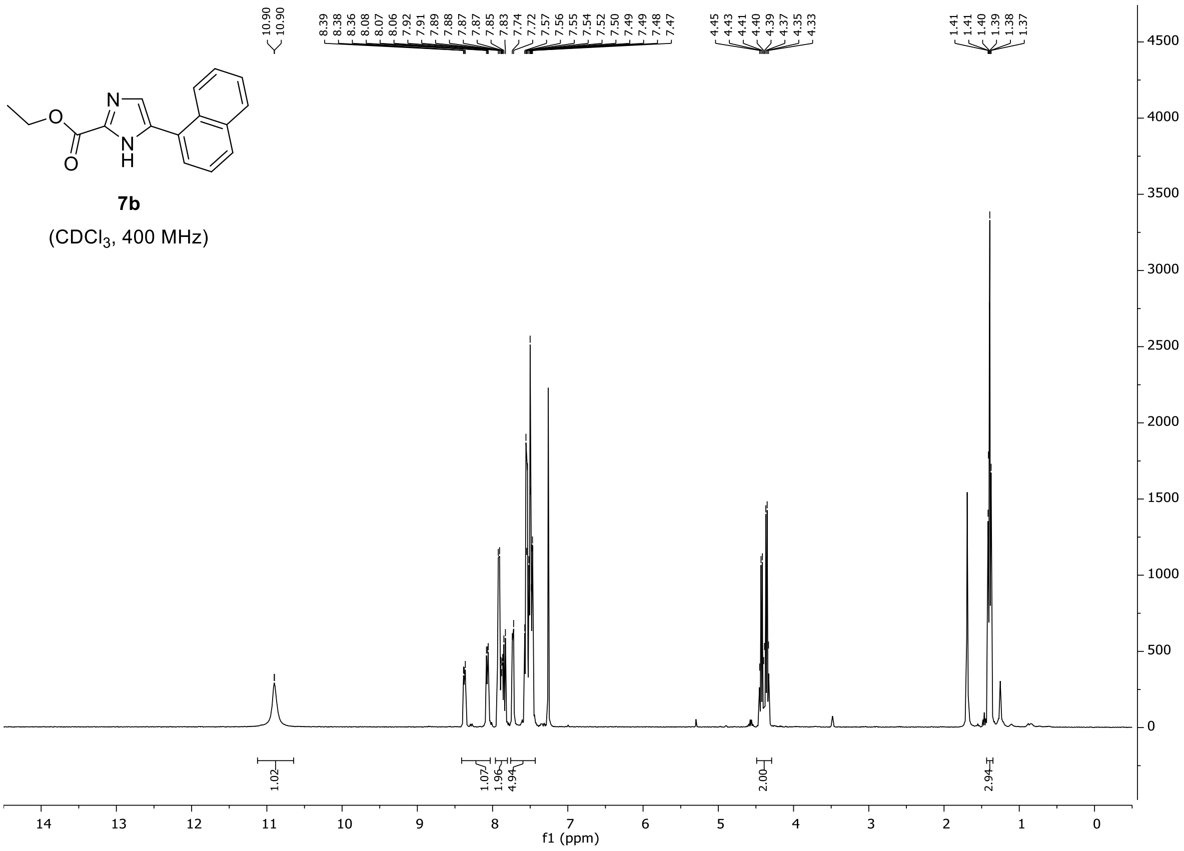

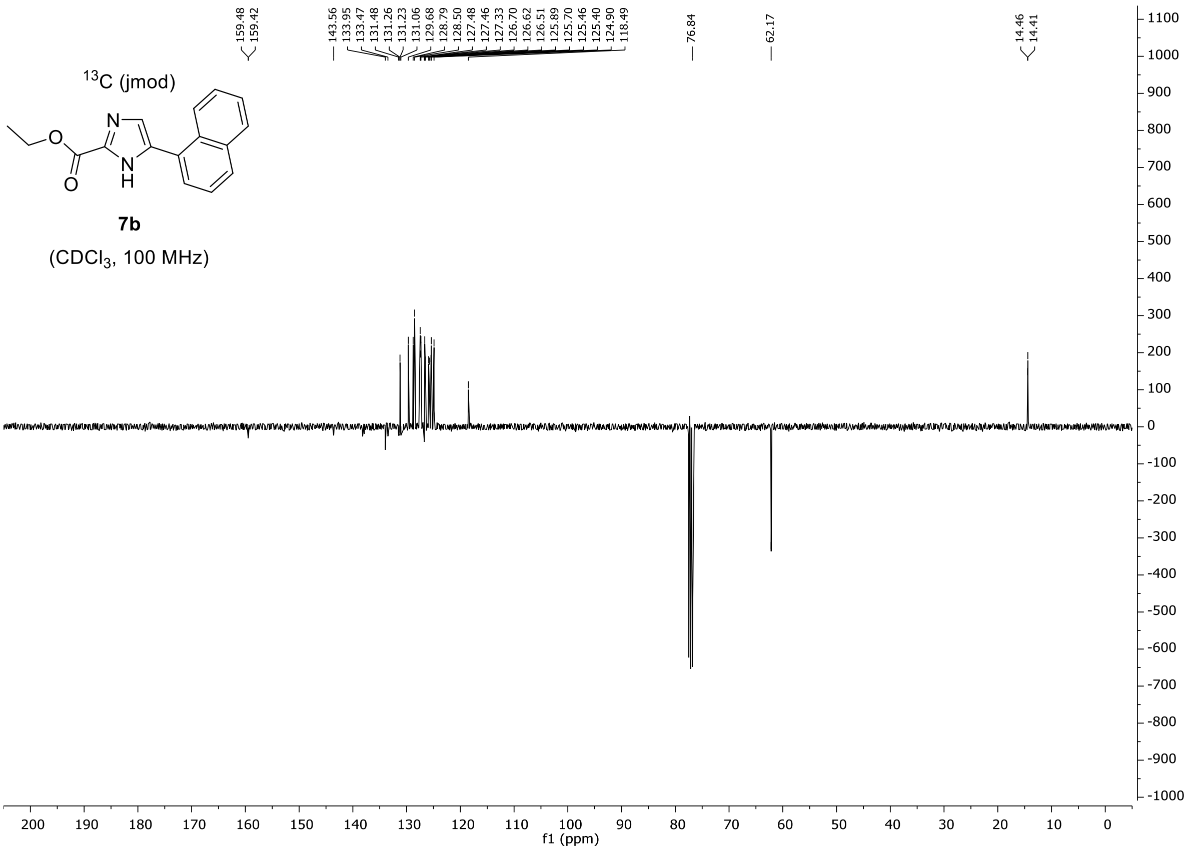

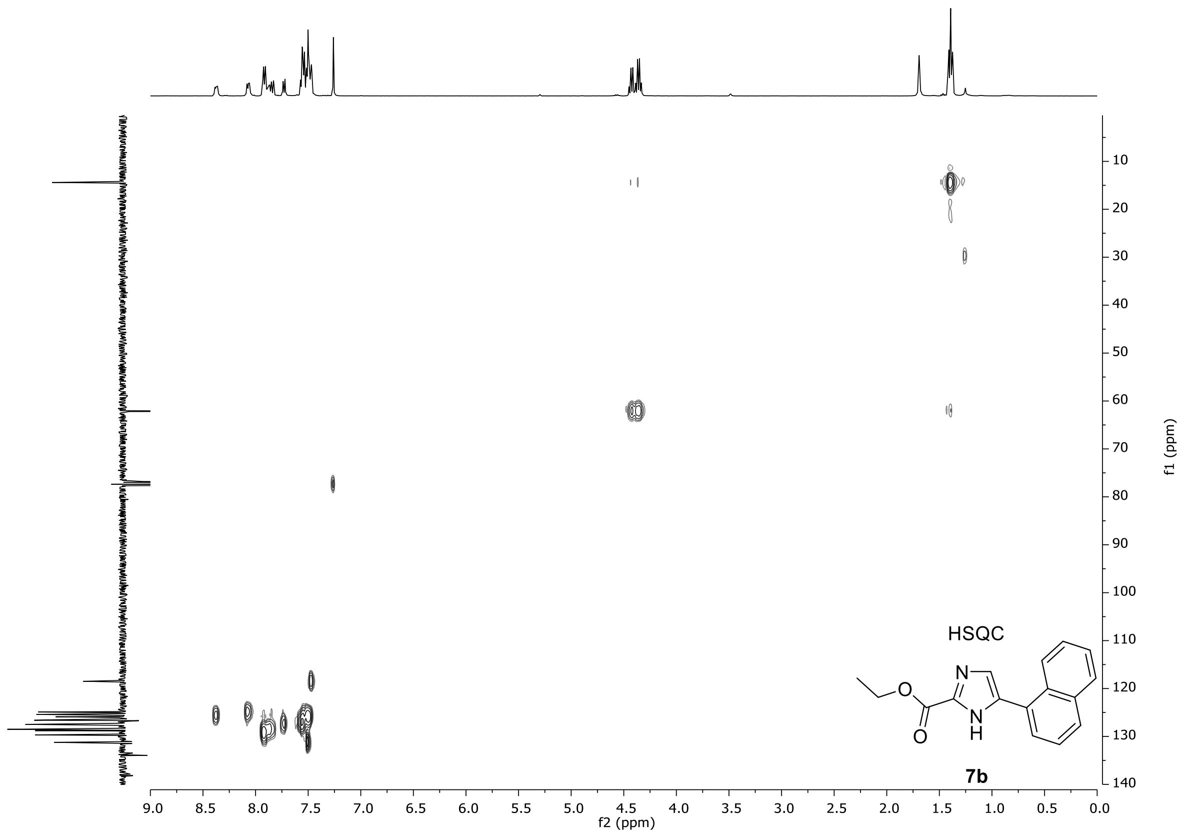
HSQC
N
N
H
O
O
7b
f2 (ppm)
f1 (ppm)

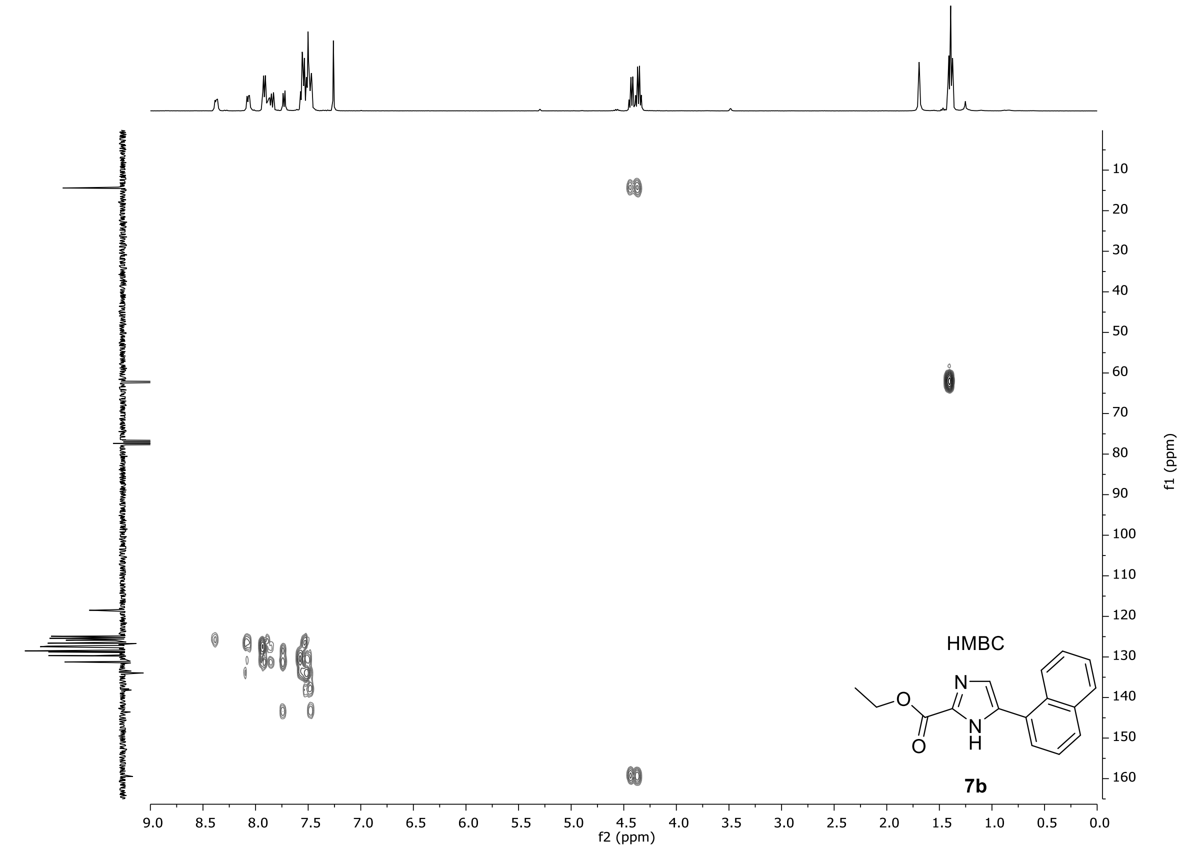
HMBC
N
N
H
O
O
7b
f2 (ppm)
f1 (ppm)

Ethyl 4(5)-(2-naphthyl)-1*H*-imidazole-2-carboxylate (7c)

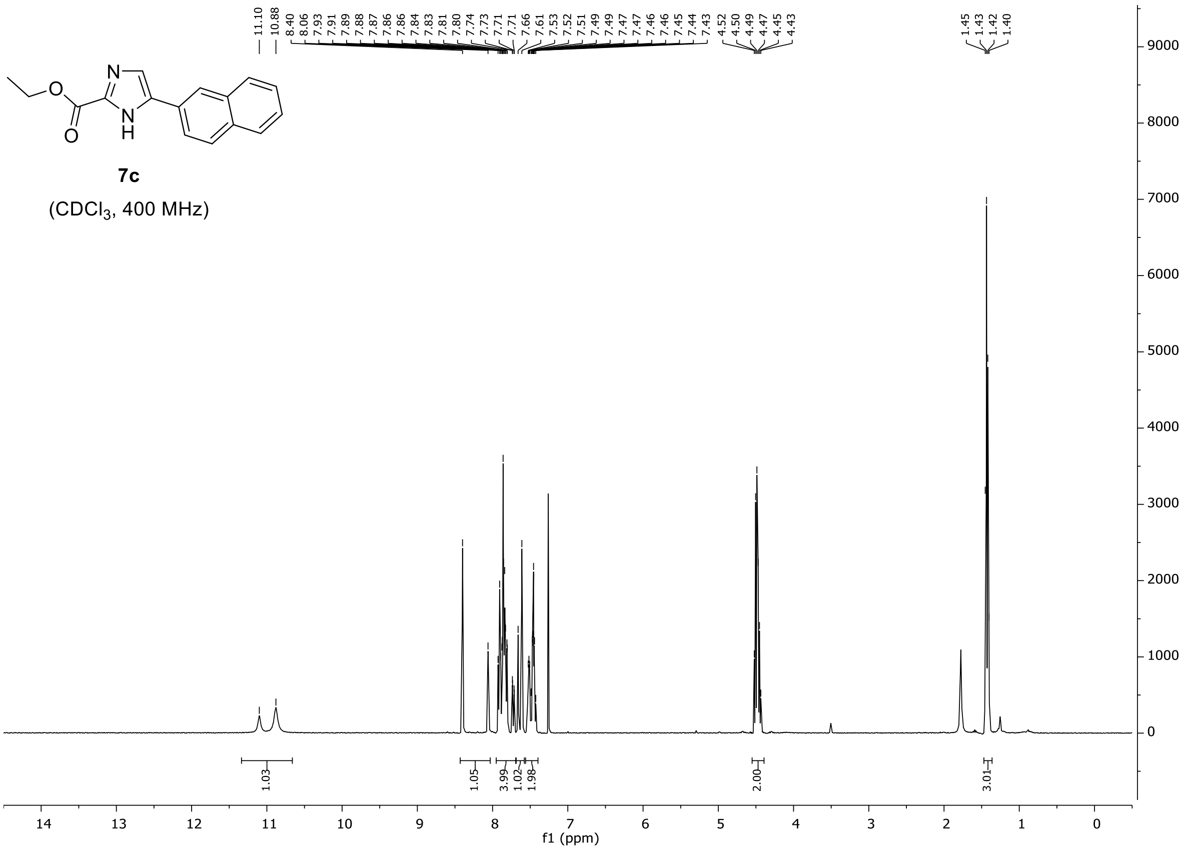

7c

($CDCl_3$, 400 MHz)

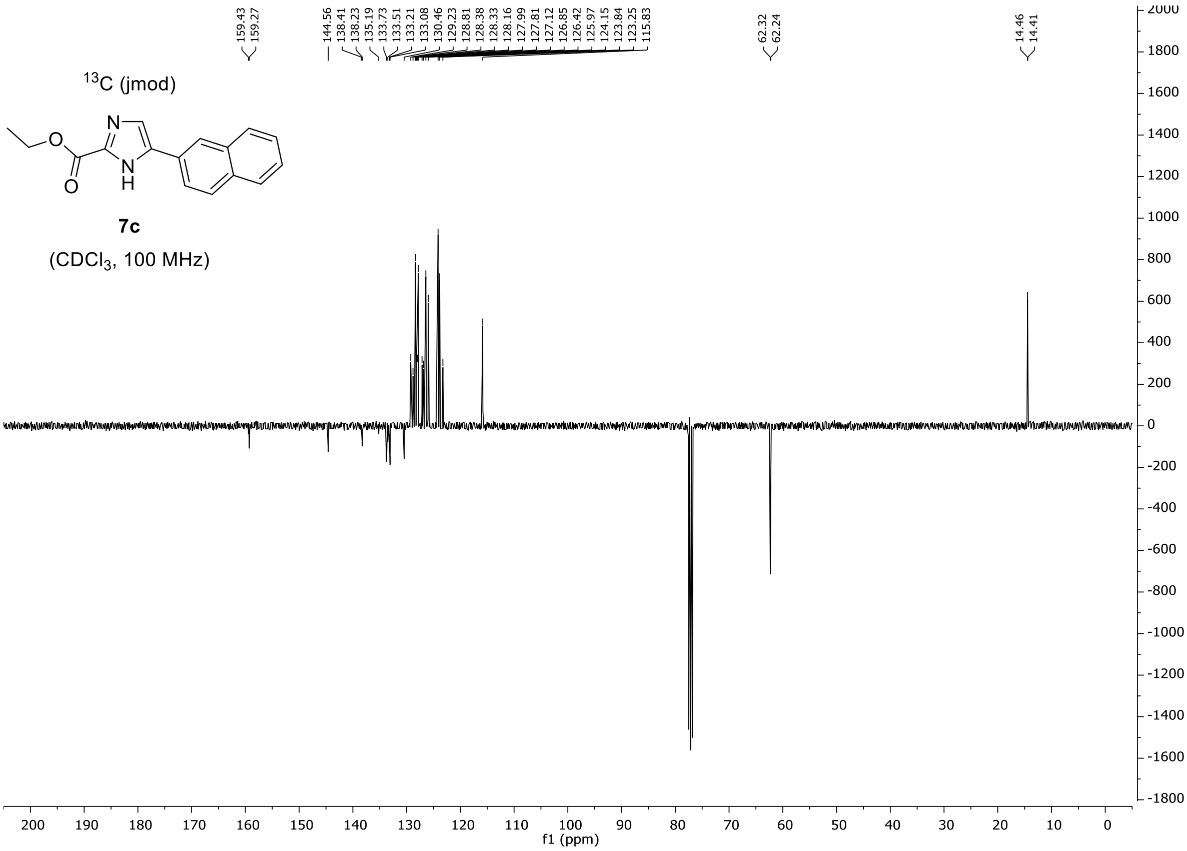

^{13}C (jmod)

7c

($CDCl_3$, 100 MHz)

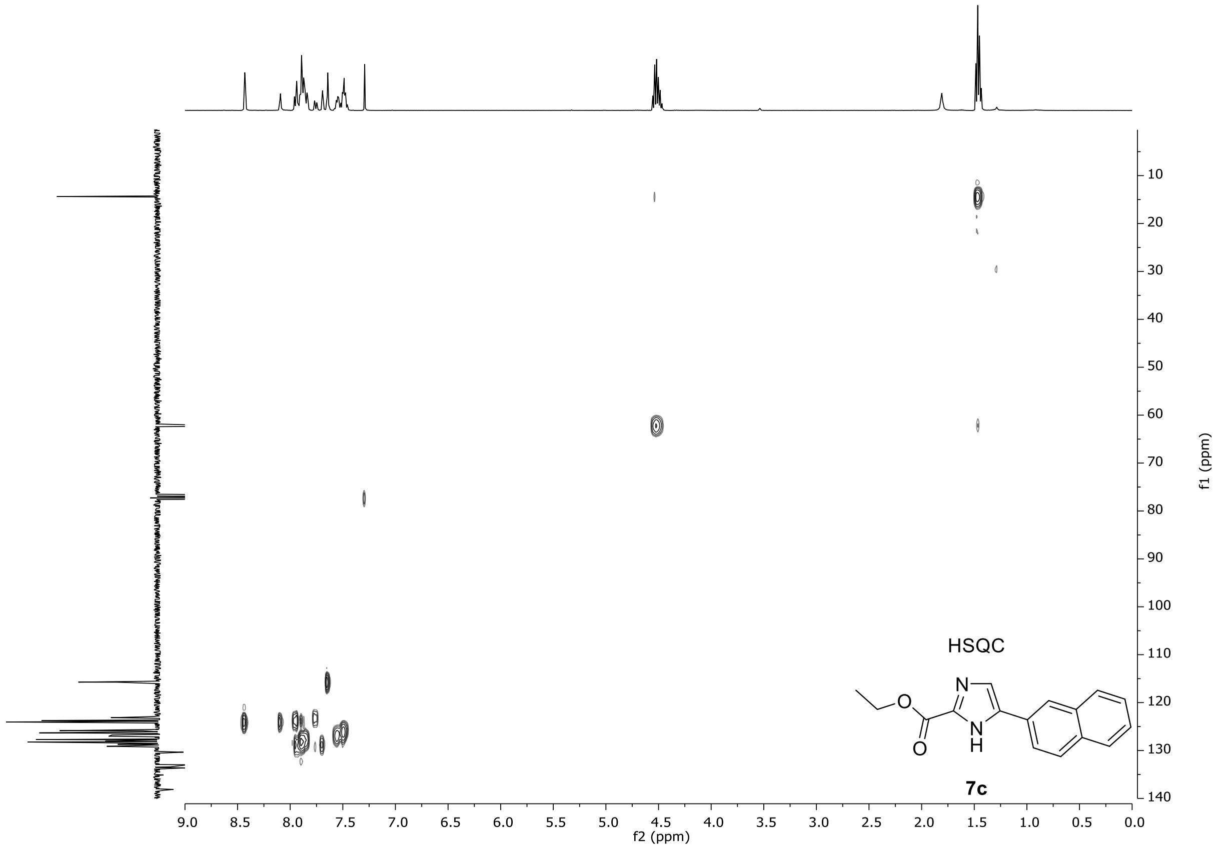
HSQC
N
O
O
N
H
7c
f1 (ppm)
f2 (ppm)

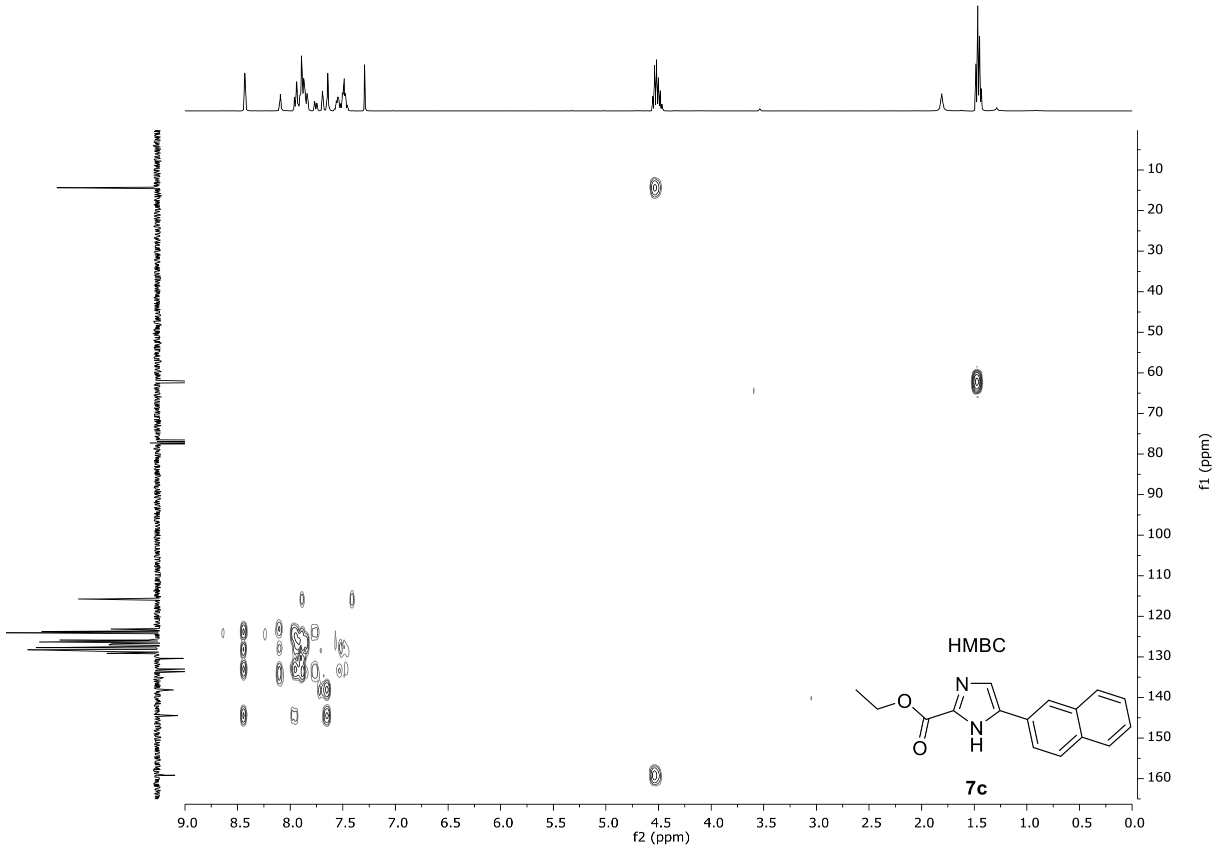
HMBC
N
O
O
N
H
7c
f1 (ppm)
f2 (ppm)

4(5)-Phenyl-1*H*-imidazole-2-carboxylic acid (8a)

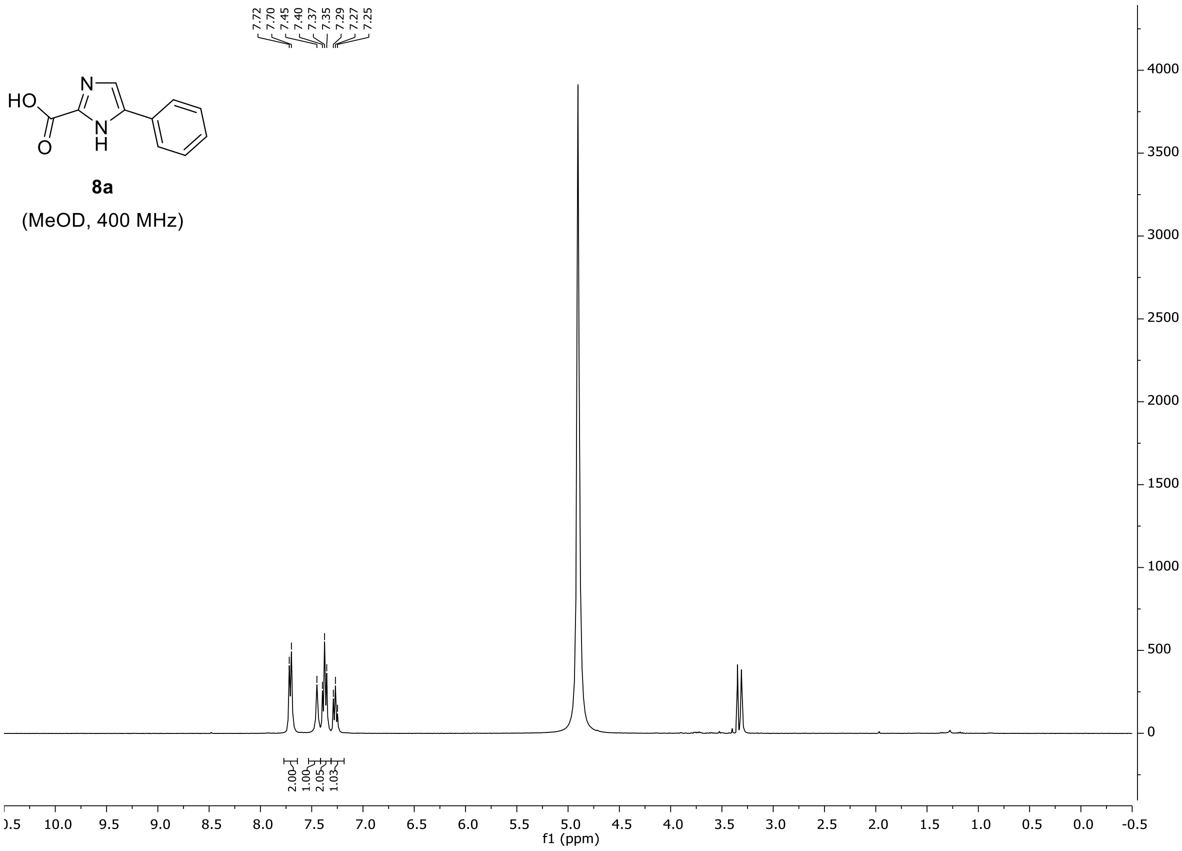

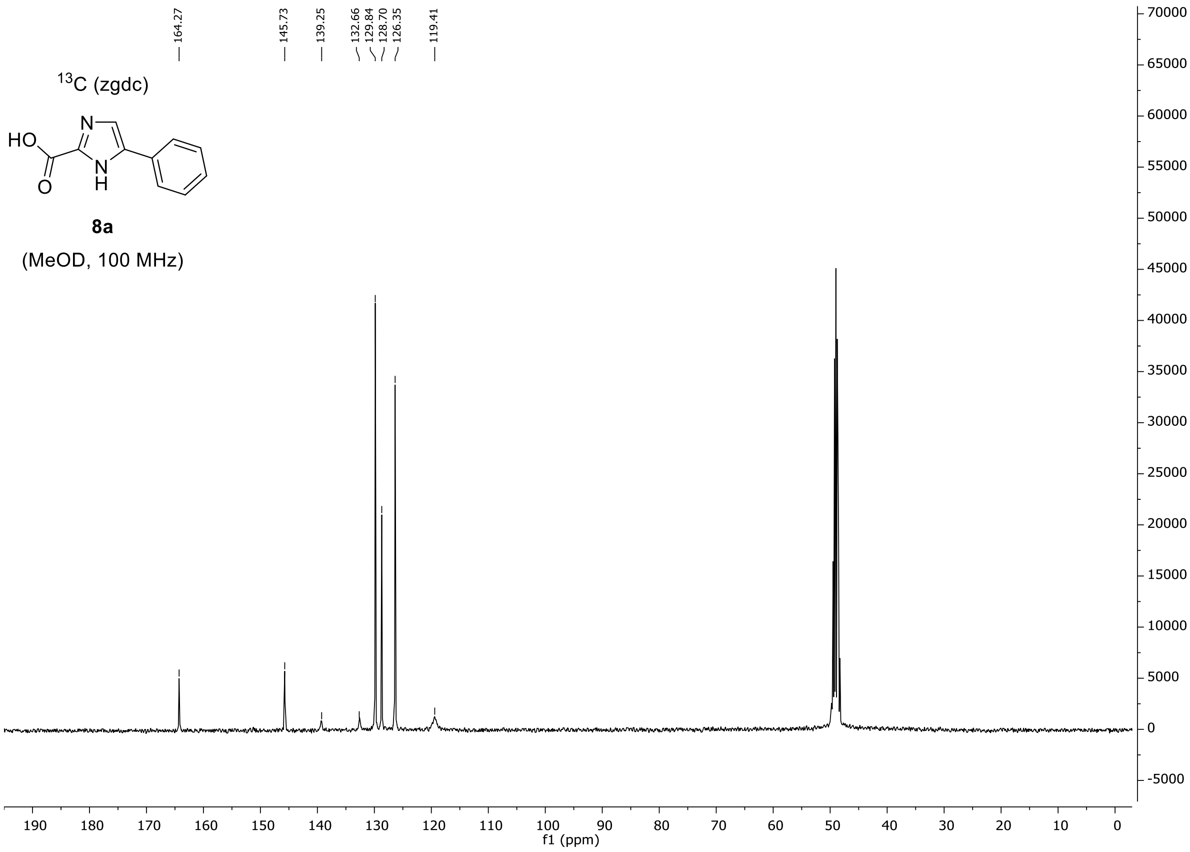

4(5)-(1-Naphthyl)-1*H*-imidazole-2-carboxylic acid (8b)

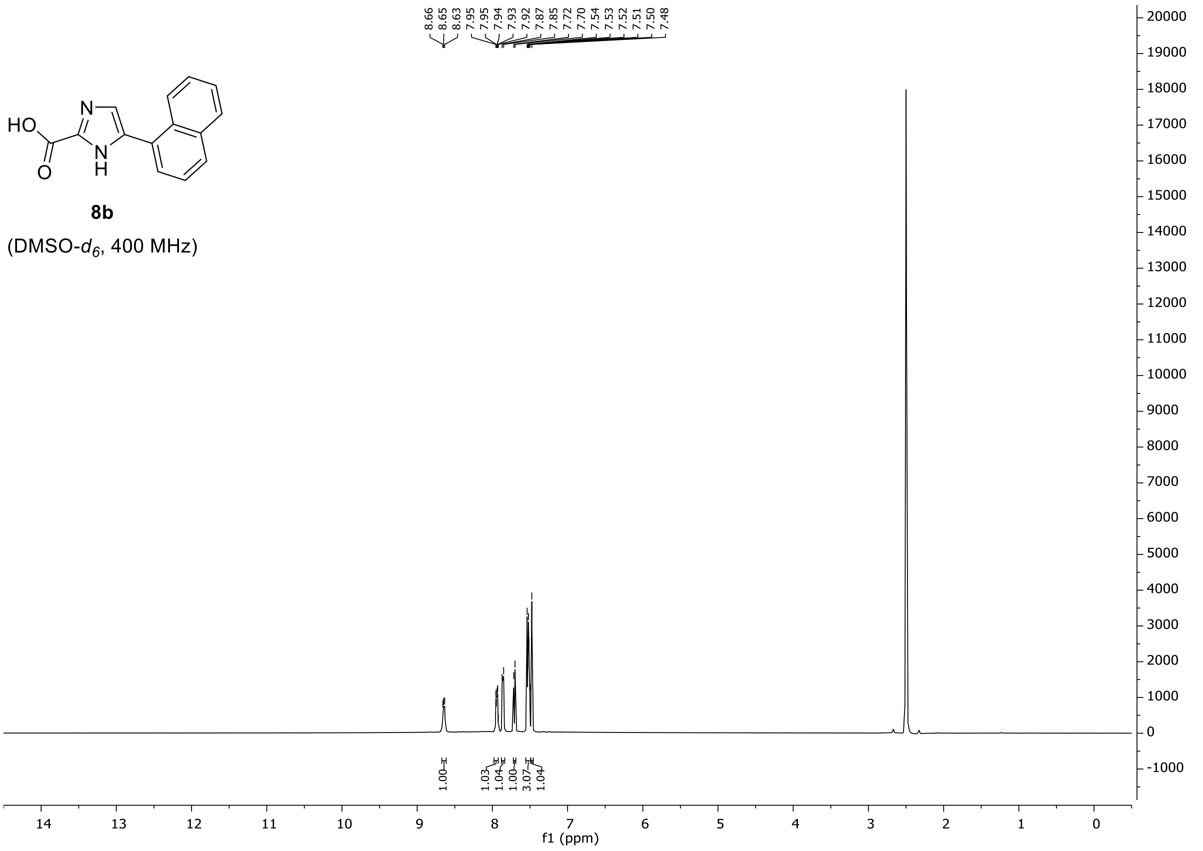

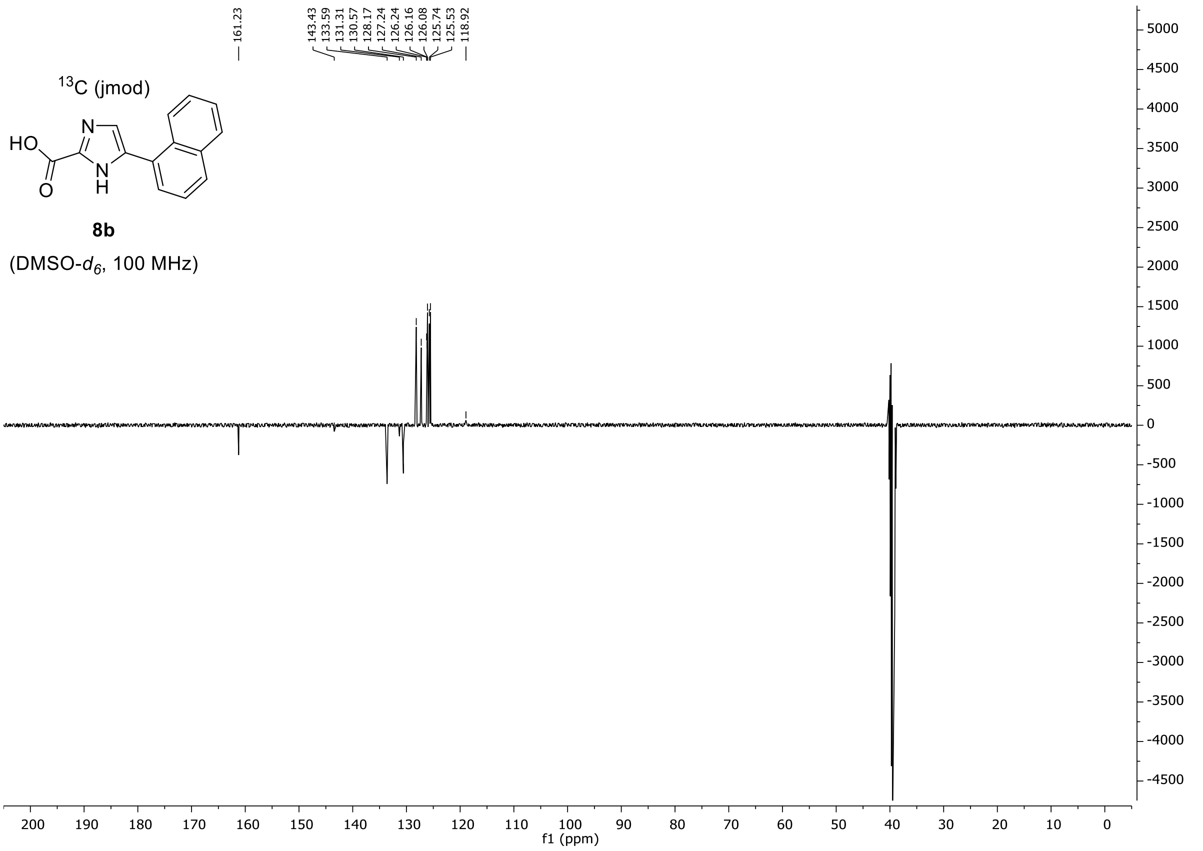

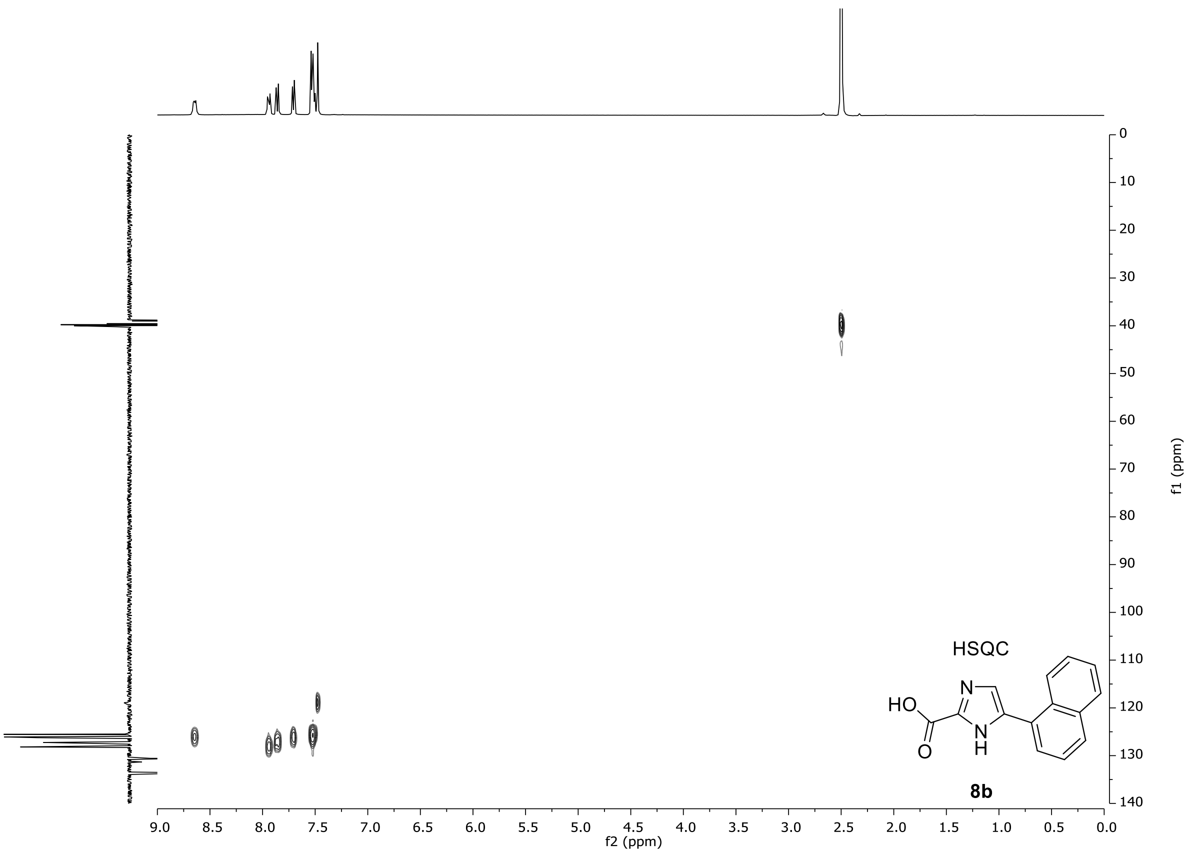
HSQC
HO
N
N
H
O
8b
f1 (ppm)
f2 (ppm)

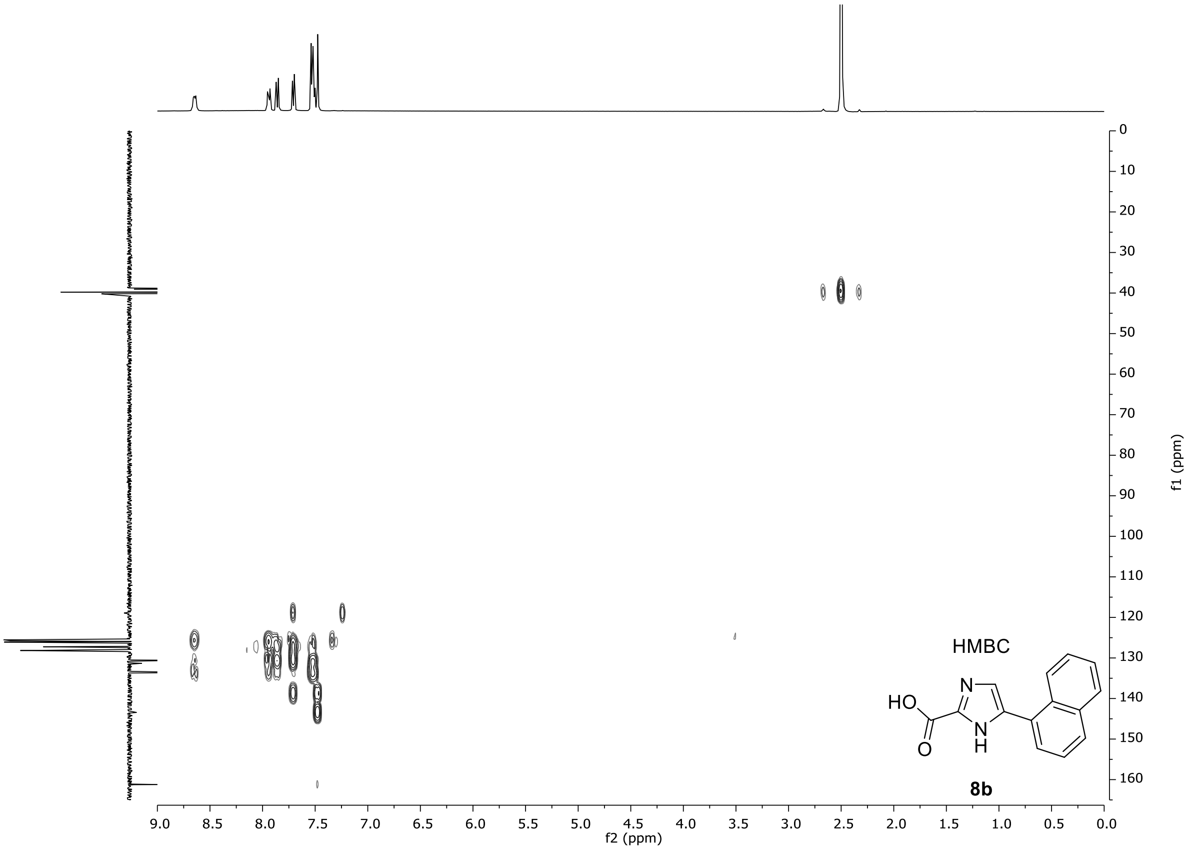
HMBC
HO
N
N
H
O
8b
f1 (ppm)
f2 (ppm)

4(5)-(2-Naphthyl)-1*H*-imidazole-2-carboxylic acid (8c)

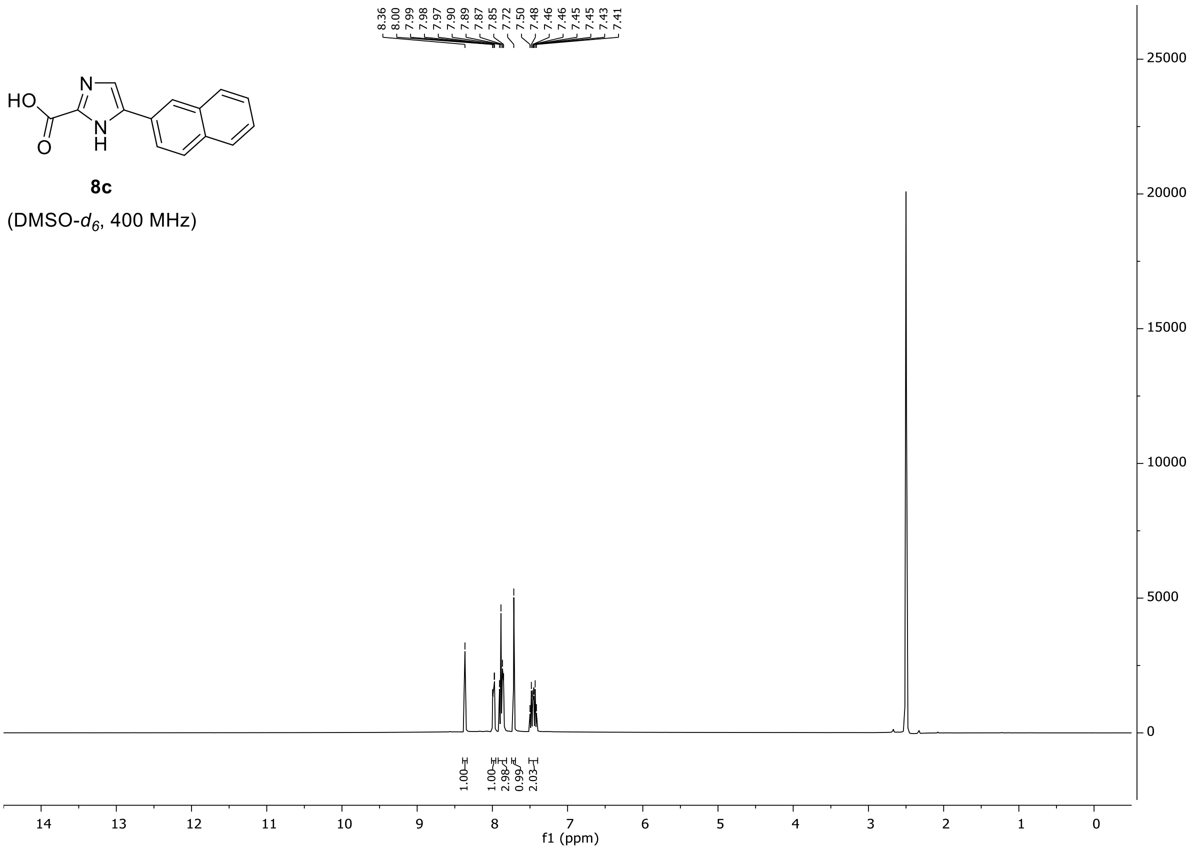

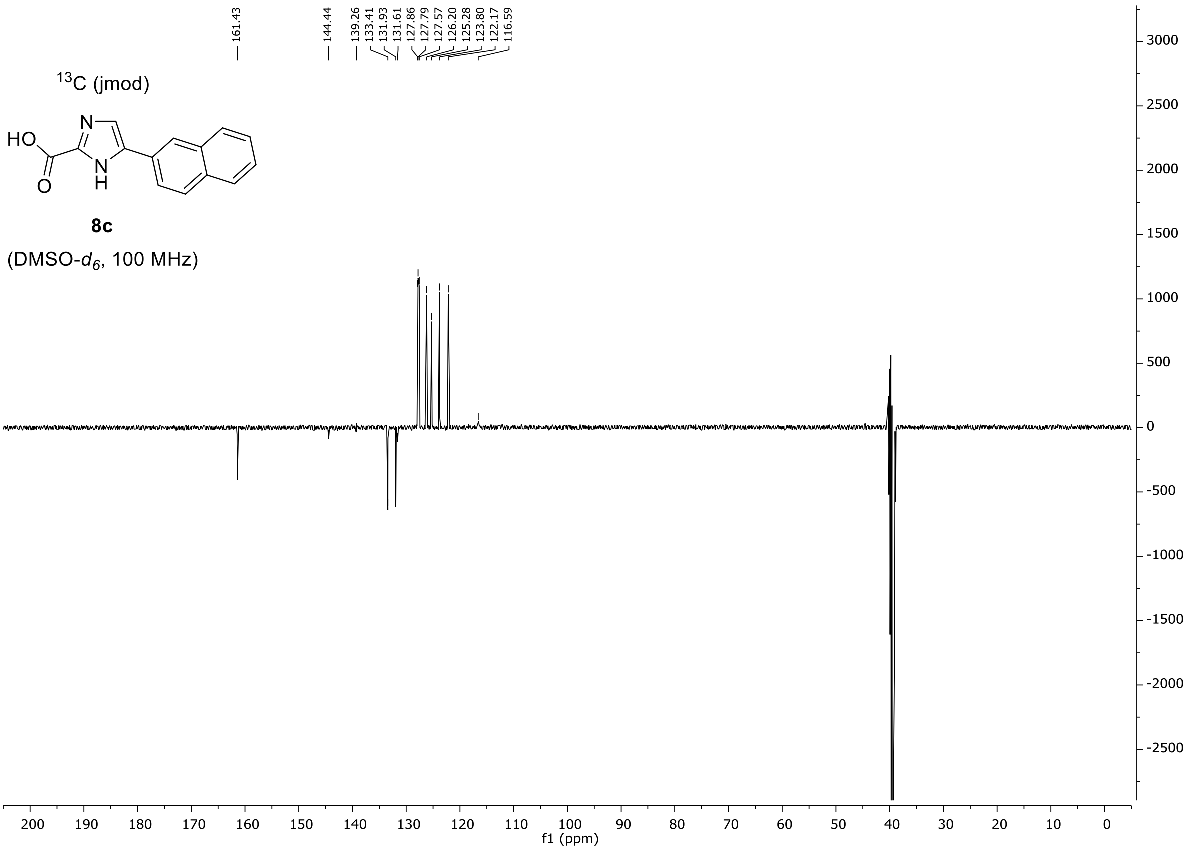

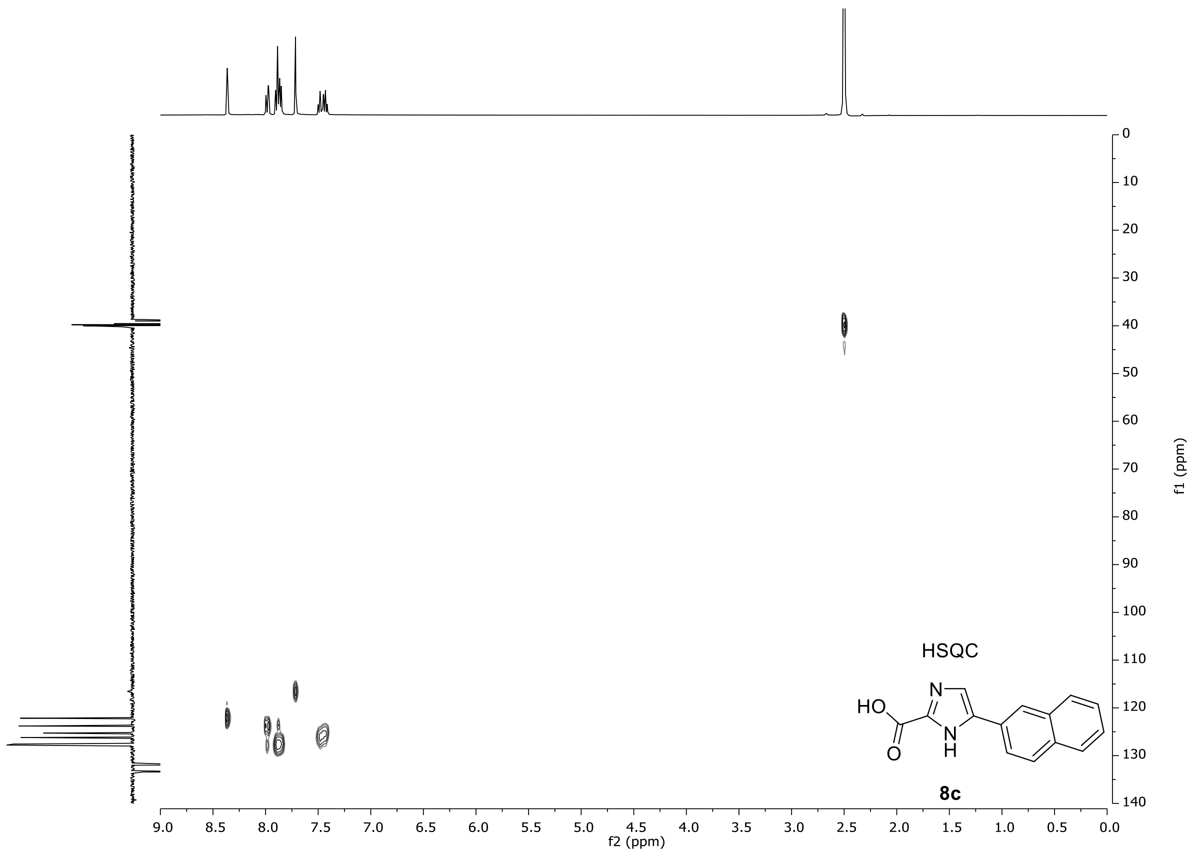
HSQC
HO
N
N
H
O
8c
f2 (ppm)
f1 (ppm)

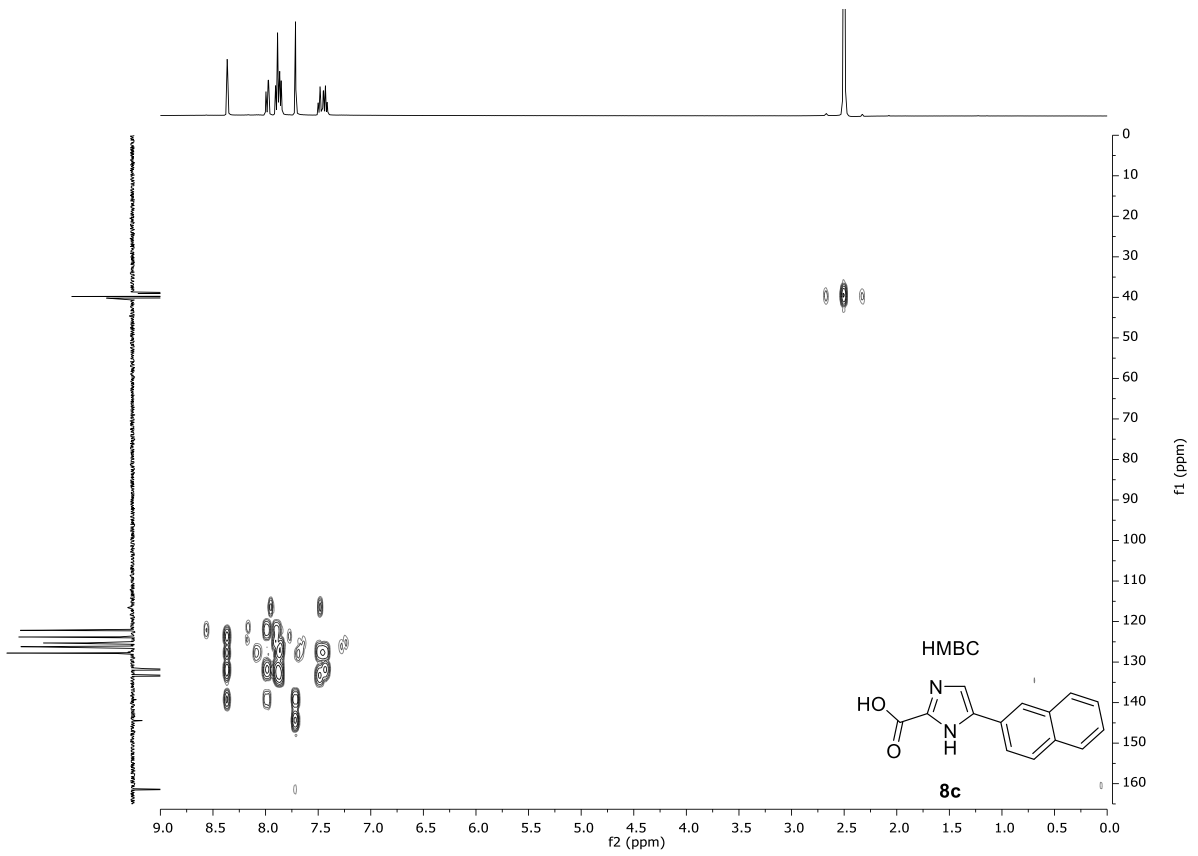
HMBC
HO
N
N
H
O
8c
f2 (ppm)
f1 (ppm)

N-(2,3,4,6-Tetra-*O*-acetyl-β-D-glucopyranosyl)-2-phenyl-1*H*-imidazole-4(5)-carboxamide (10a)

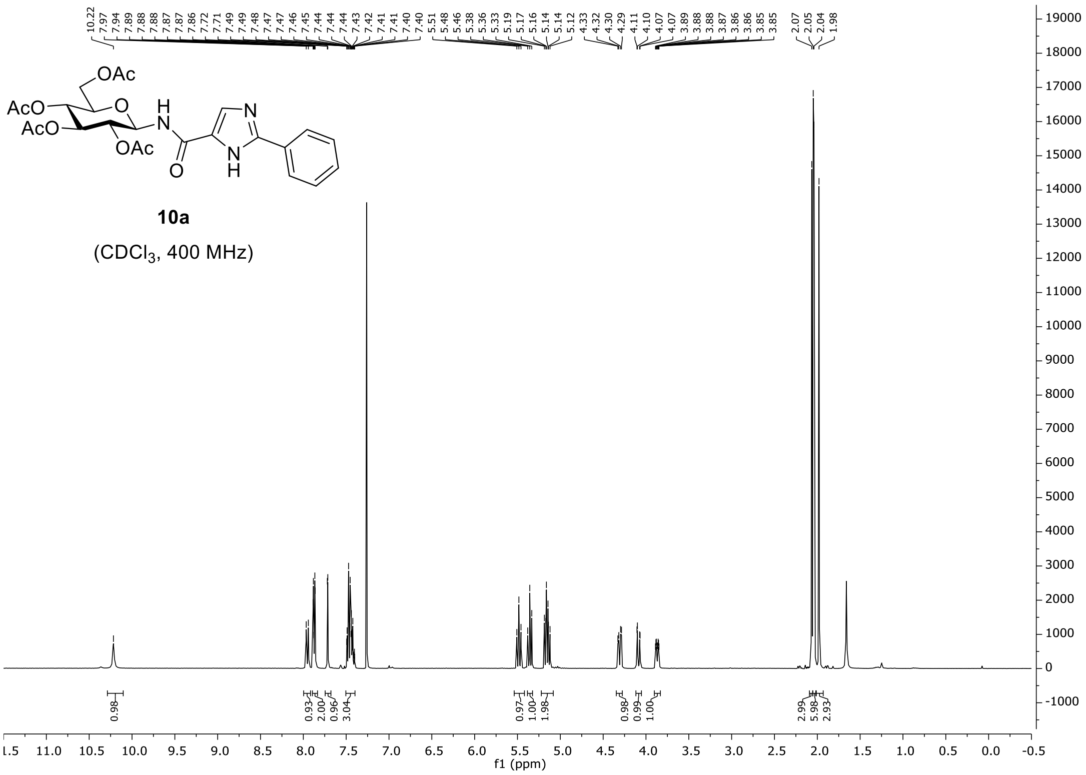

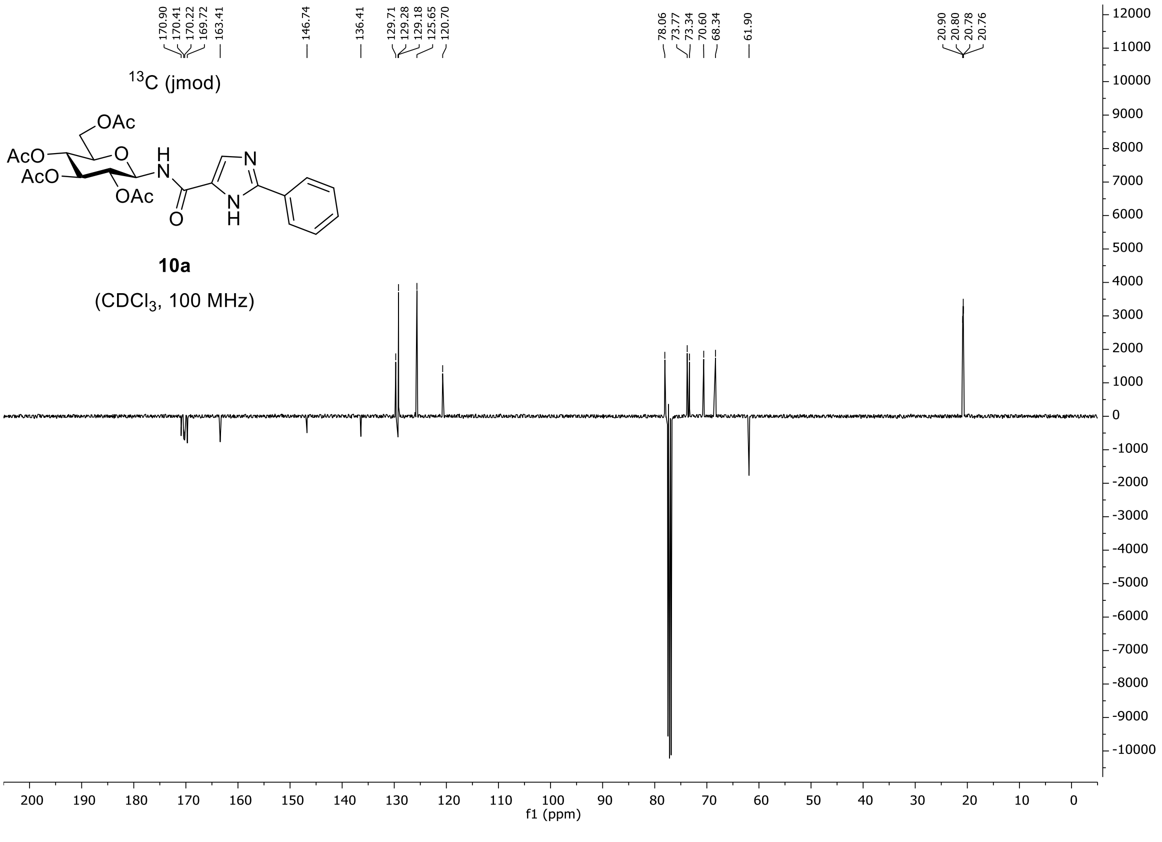

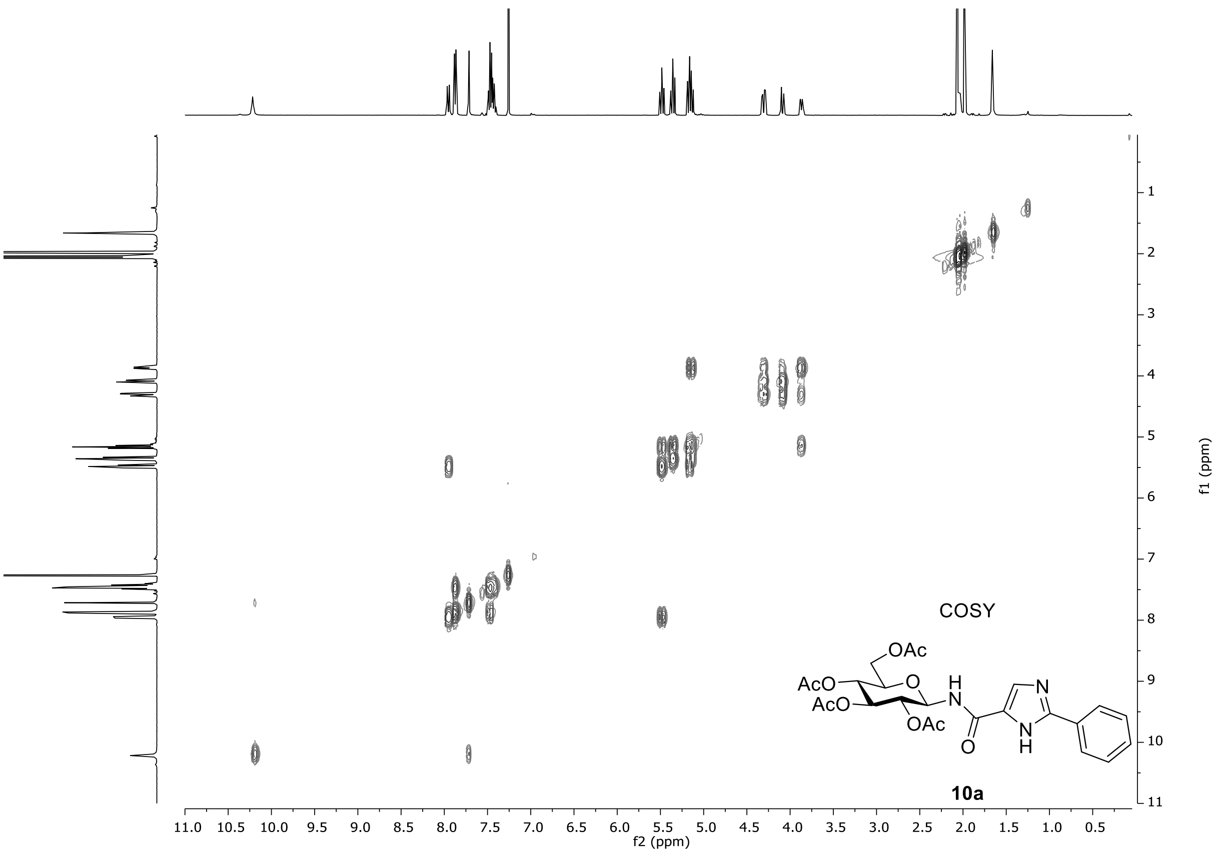
COSY
OAc
AcO
AcO
O
H
N
OAc
O
N
N
H
10a
f2 (ppm)
f1 (ppm)

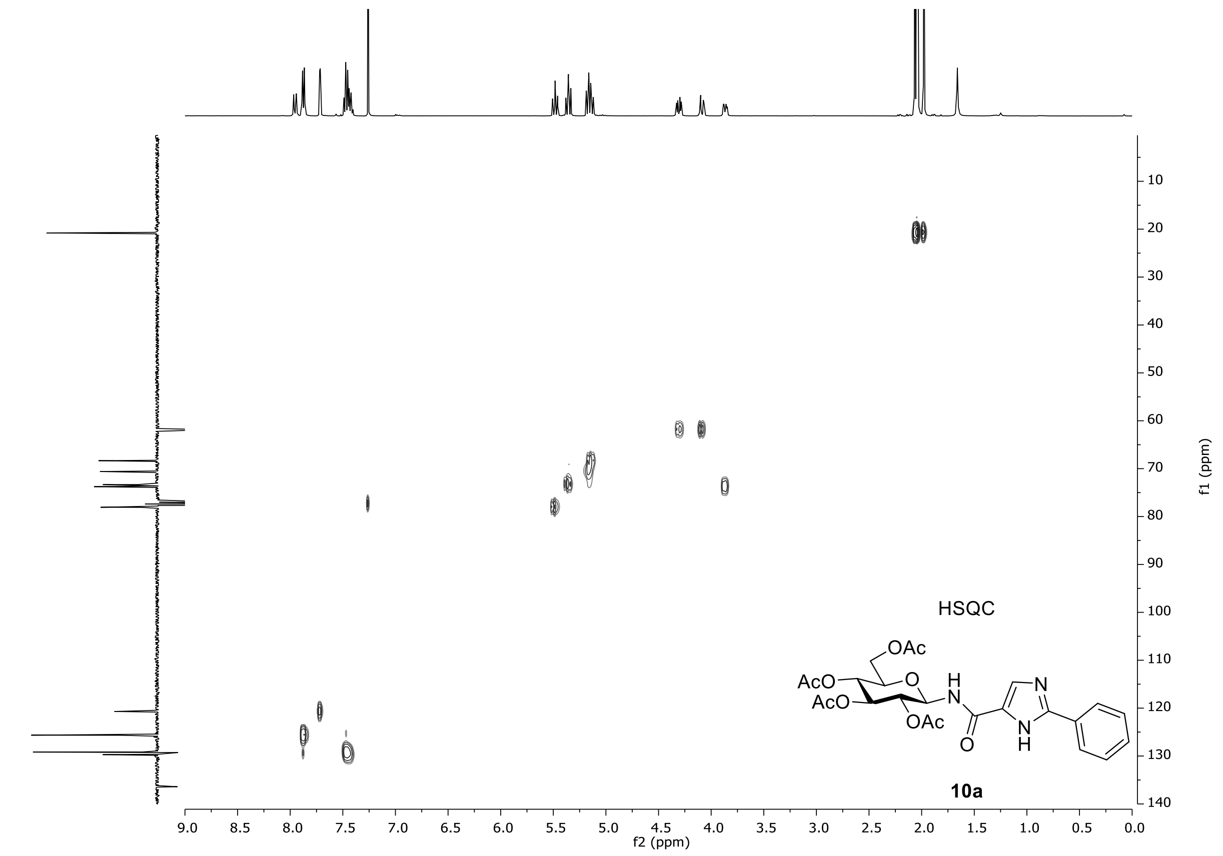
HSQC
OAc
AcO
AcO
O
H
N
OAc
O
N
N
H
10a
f2 (ppm)
f1 (ppm)

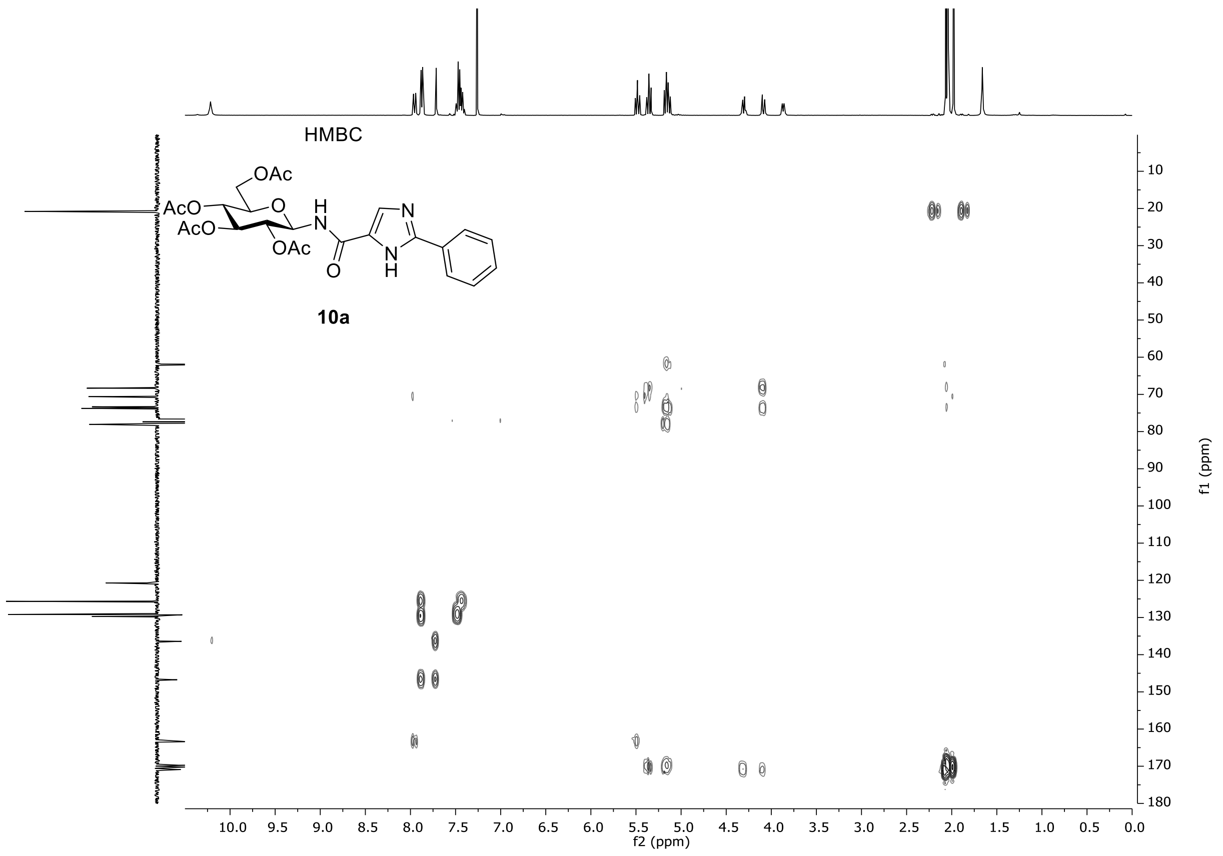
HMBC
OAc
AcO
AcO
O
H
N
OAc
O
N
H
N
10a
f1 (ppm)
f2 (ppm)

N-(2,3,4,6-Tetra-O-acetyl-β-D-glucopyranosyl)-2-(1-naphthyl)-1H-imidazole-4(5)-carboxamide (10b)

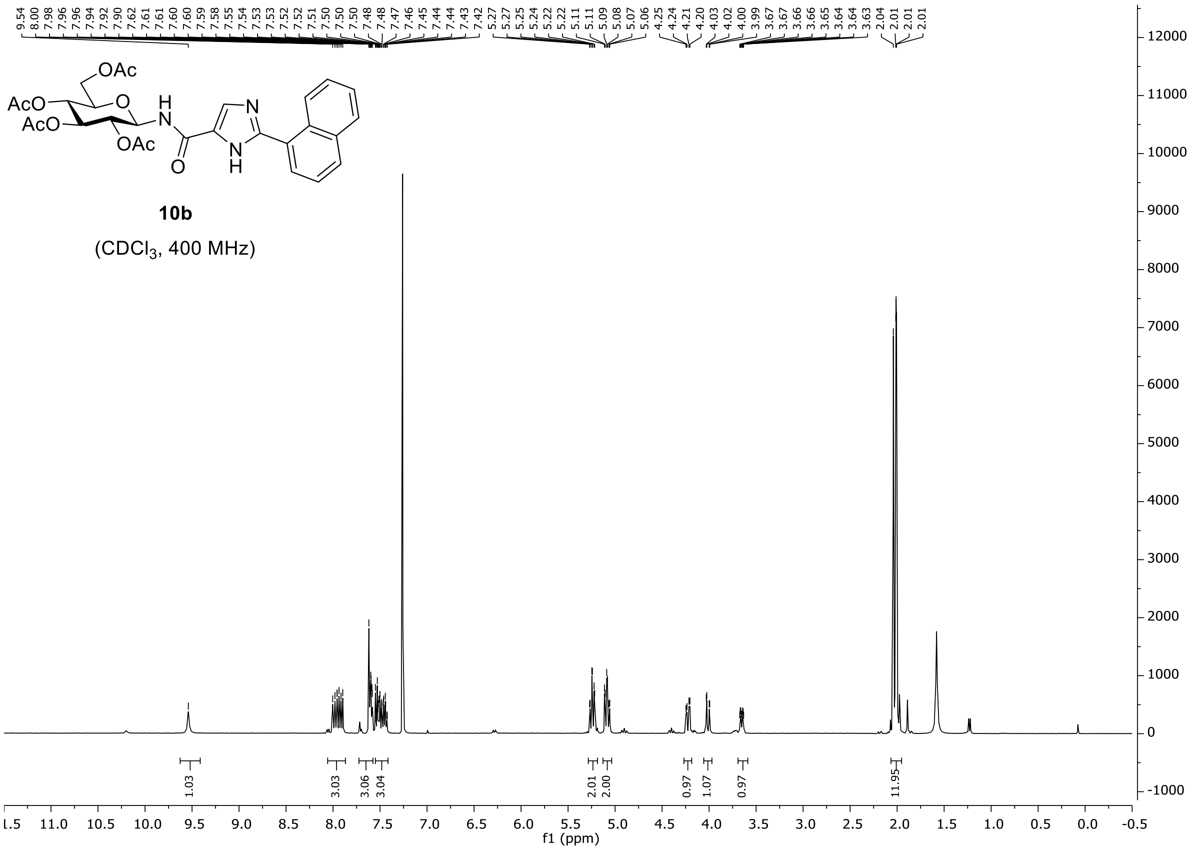

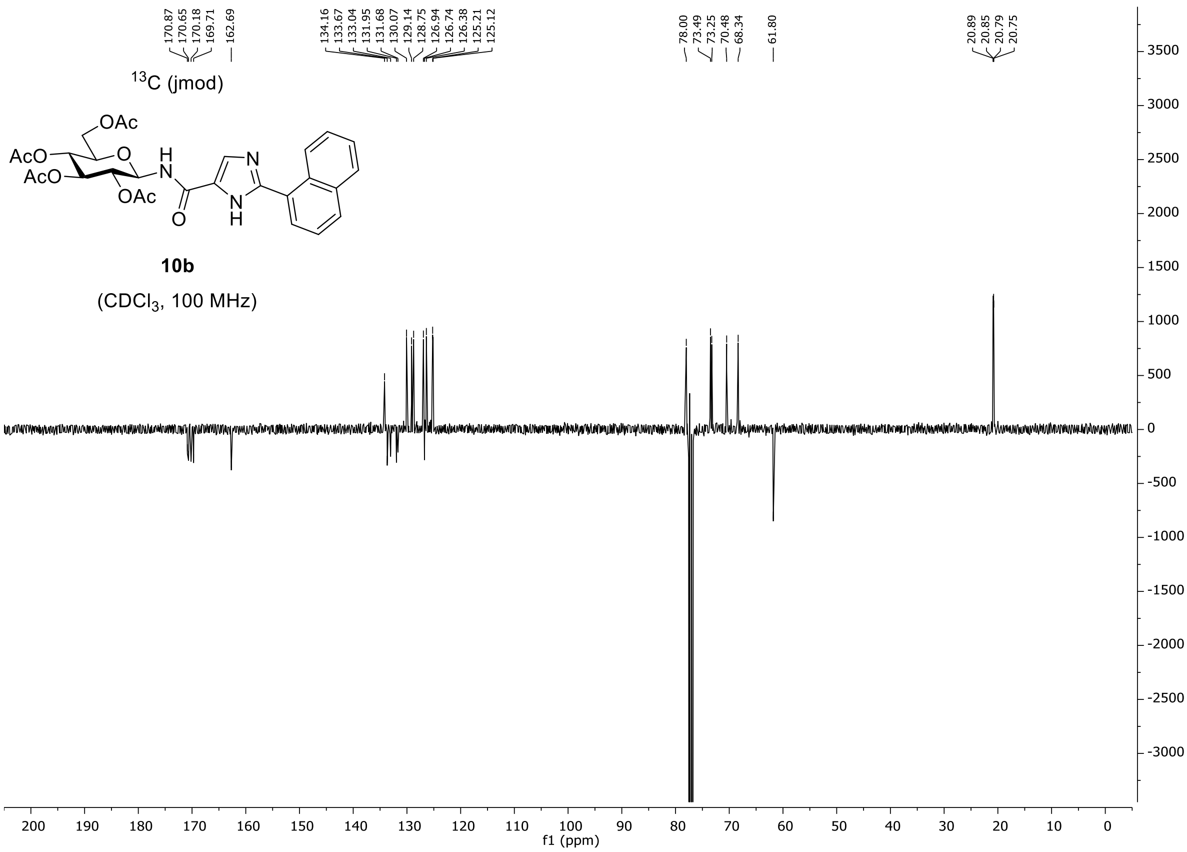

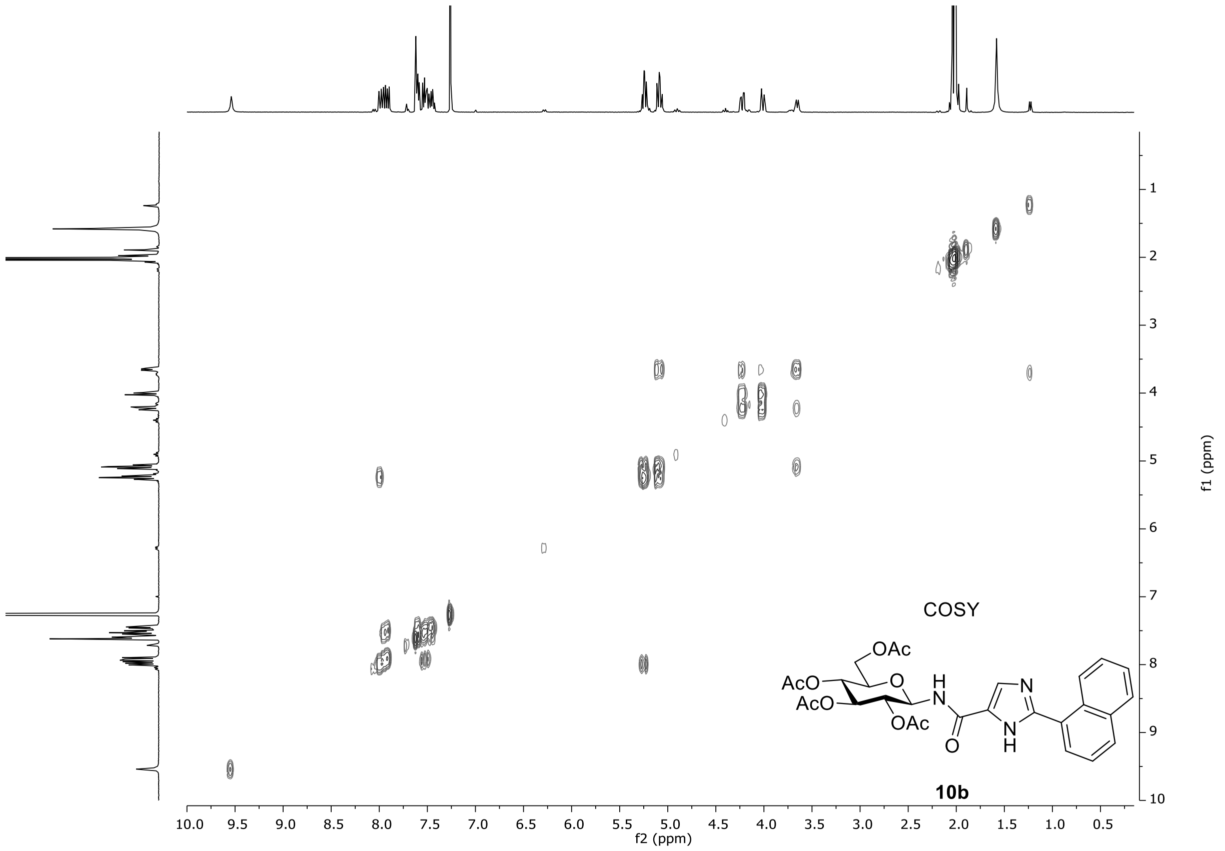
COSY
OAc
AcO
AcO
O
H
N
N
OAc
O
N
H
10b
f1 (ppm)
f2 (ppm)

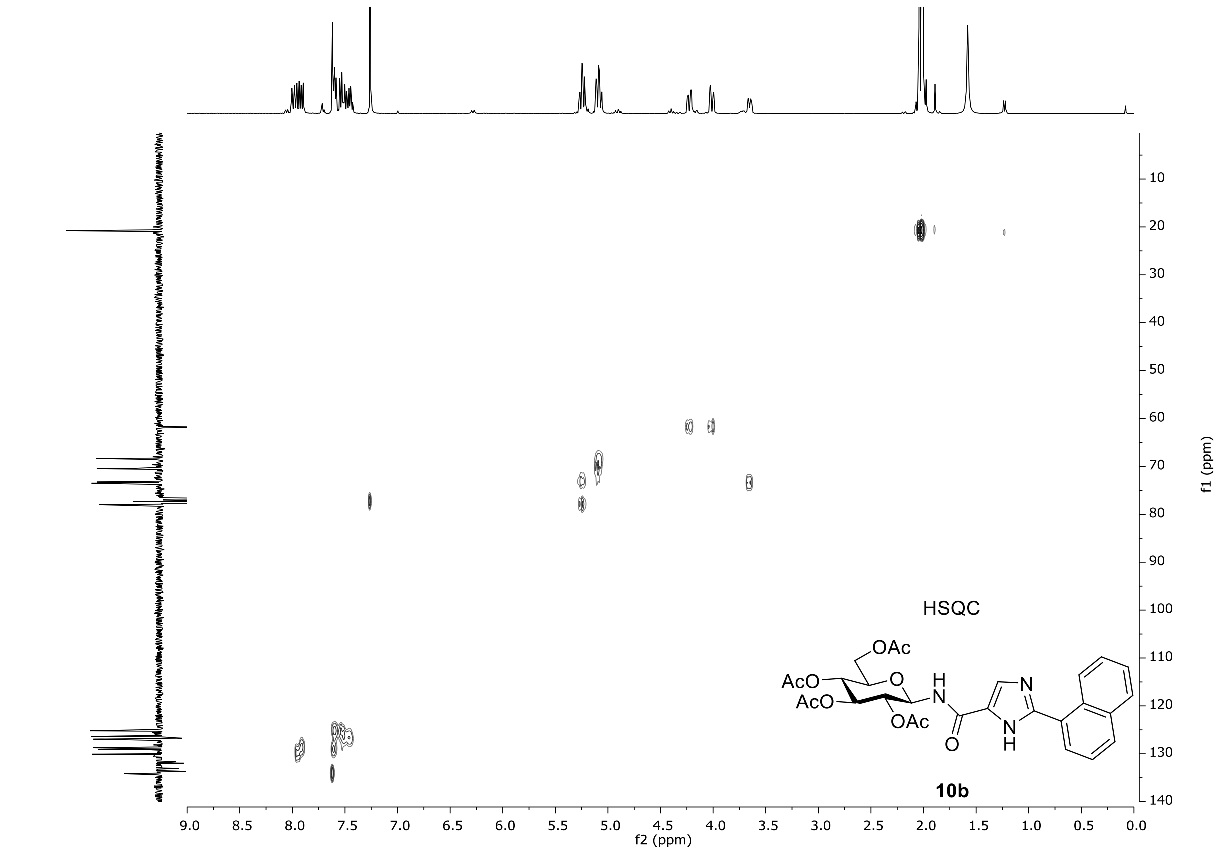
HSQC
OAc
AcO
AcO
O
H
N
N
OAc
O
N
H
10b
f1 (ppm)
f2 (ppm)

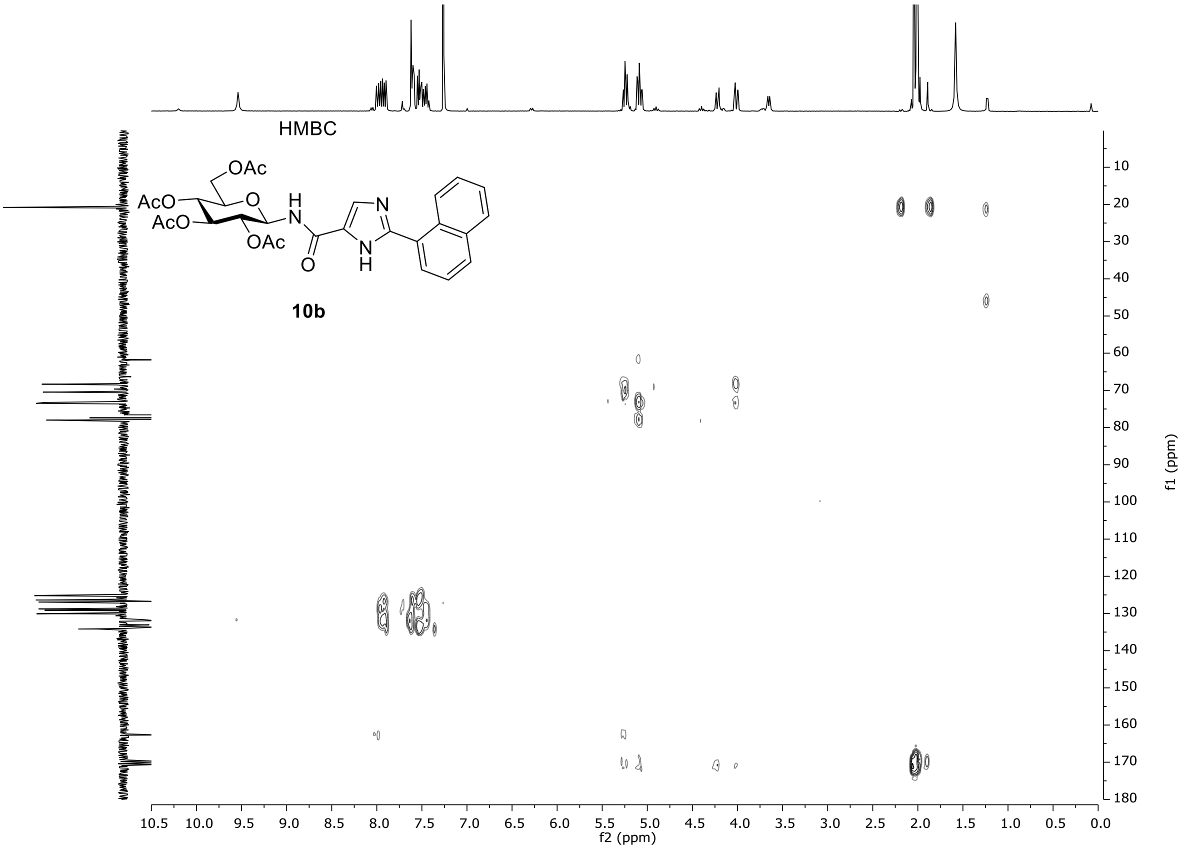
HMBC
OAc
AcO
AcO
O
OAc
H
N
O
N
N
H
10b
f1 (ppm)
f2 (ppm)

N-(2,3,4,6-Tetra-*O*-acetyl-β-D-glucopyranosyl)-2-(2-naphthyl)-1*H*-imidazole-4(5)-carboxamide (10c)

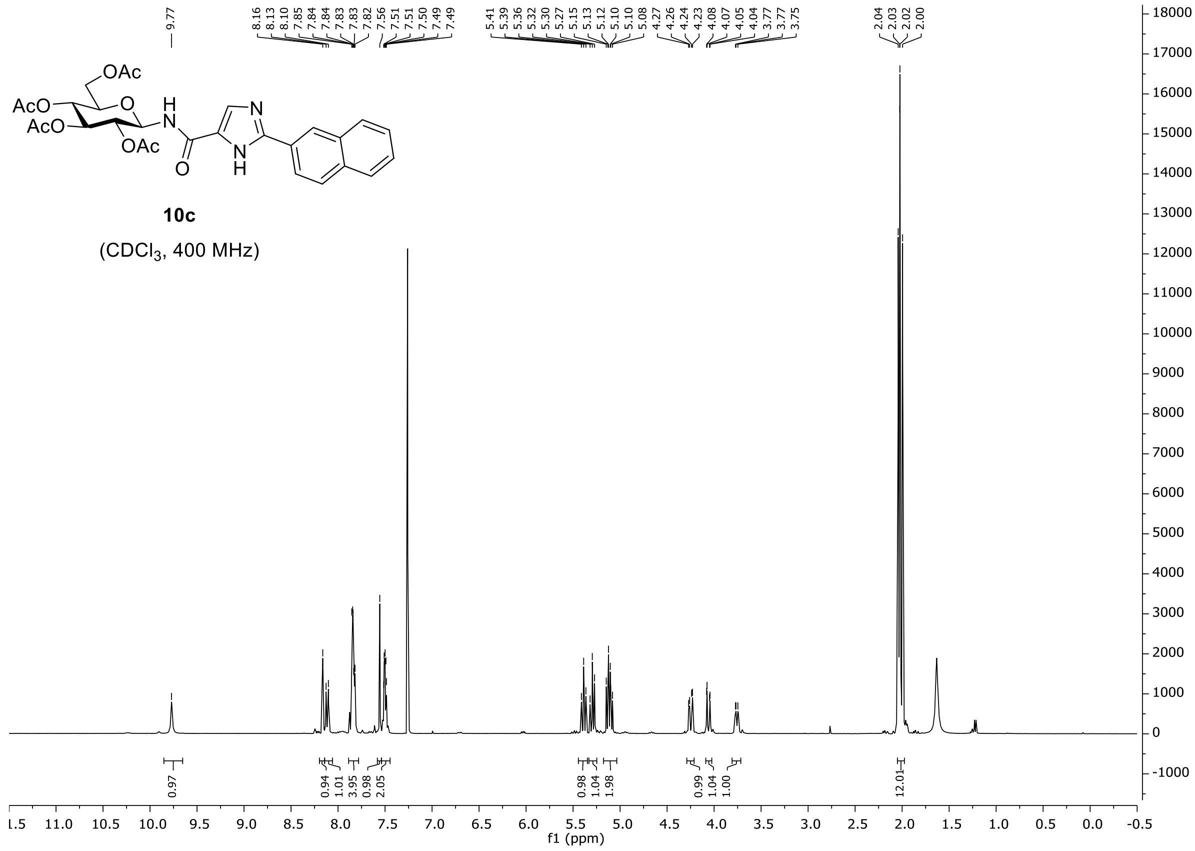

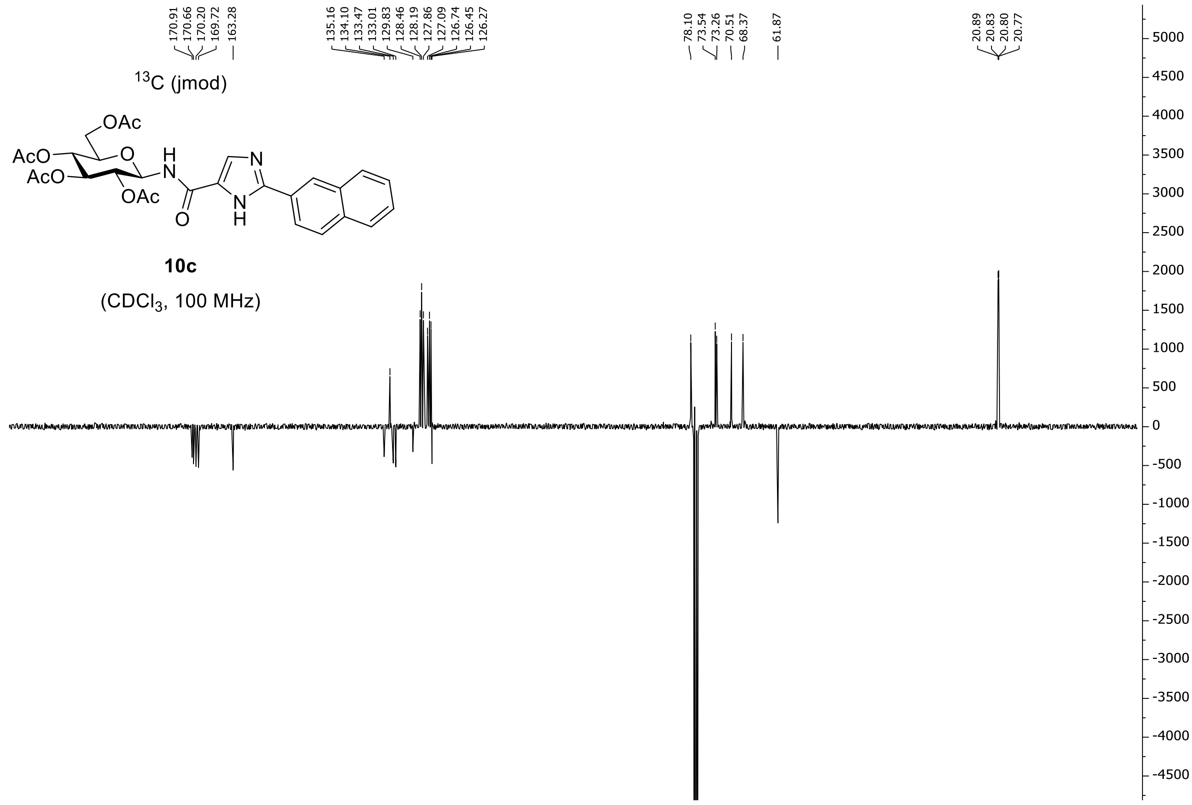

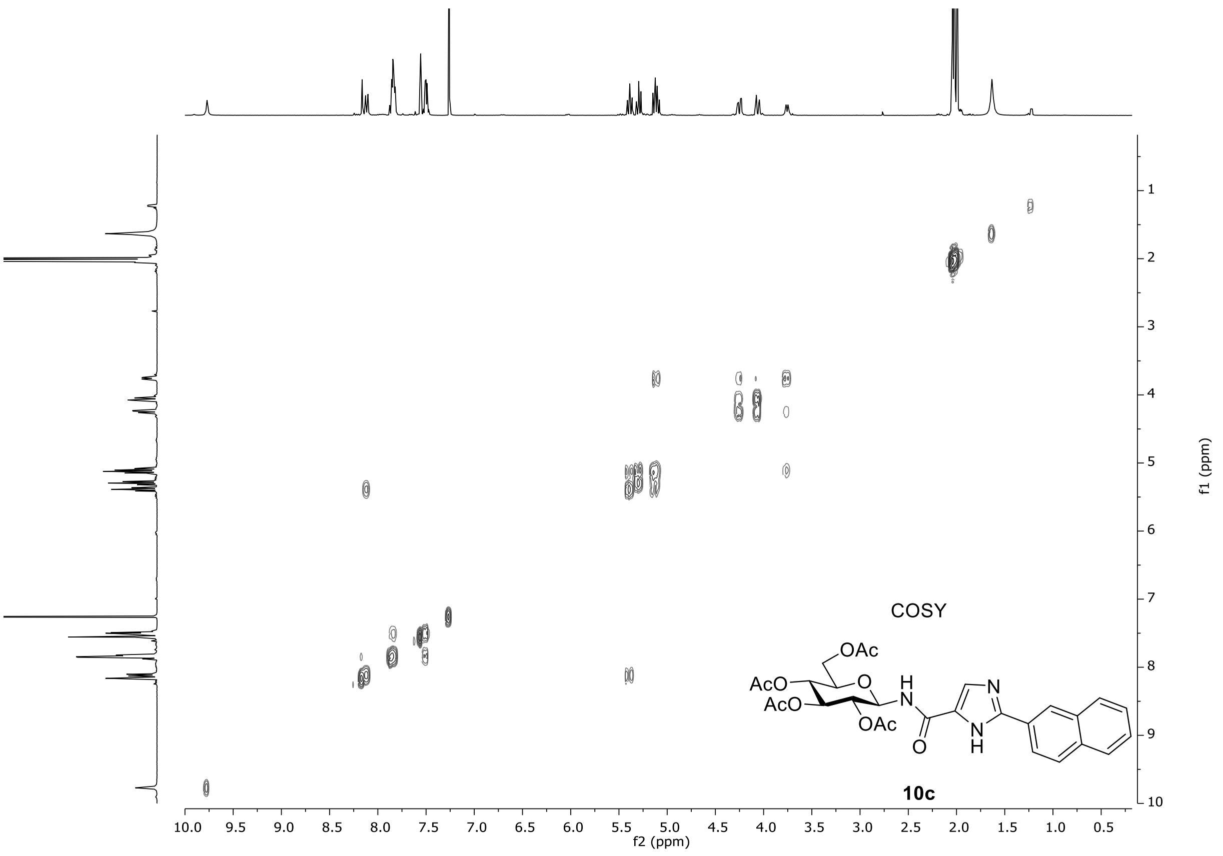
COSY
OAc
AcO
AcO
O
H
N
N
OAc
O
N
H
10c
f2 (ppm)
f1 (ppm)

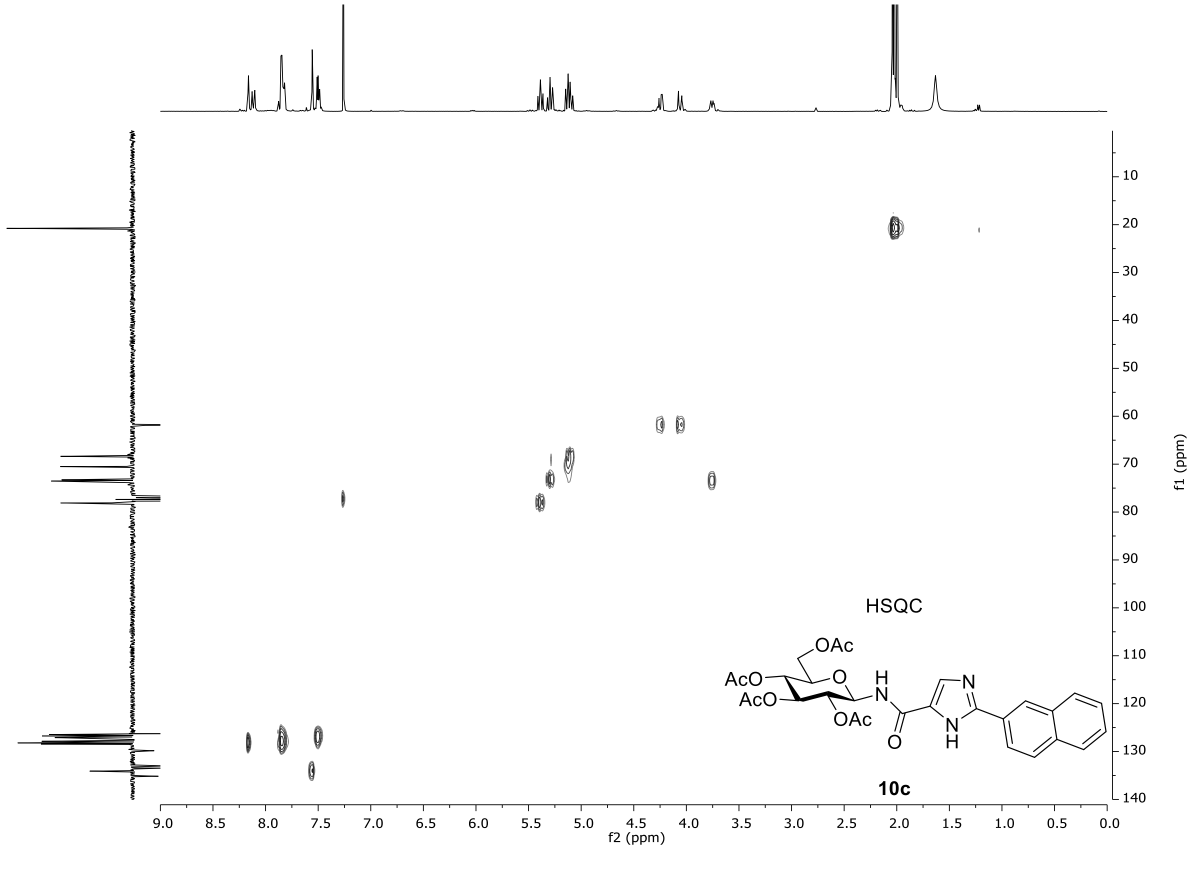
HSQC
OAc
AcO
AcO
O
H
N
N
OAc
O
N
H
10c
f2 (ppm)
f1 (ppm)

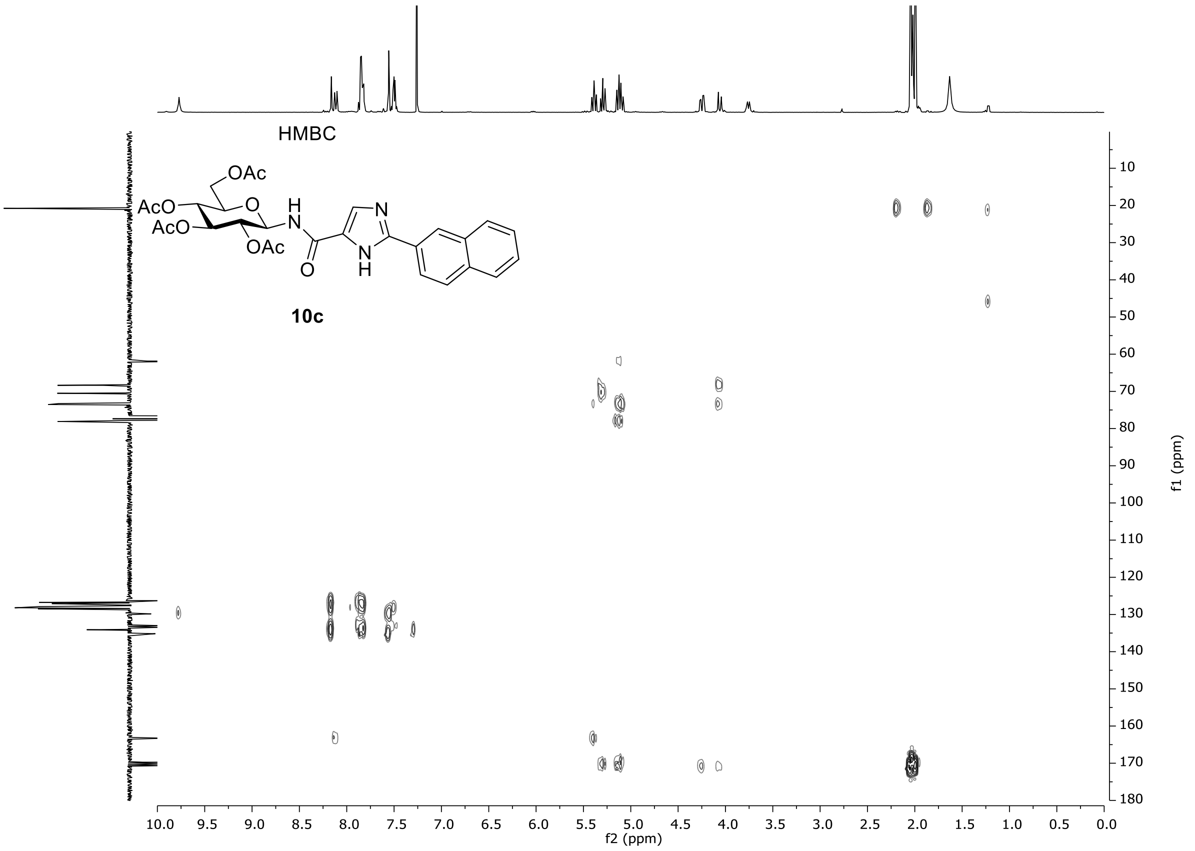
HMBC
OAc
AcO
AcO
OAc
H
N
O
N
N
H
O
10c
f1 (ppm)
f2 (ppm)

N-(2,3,4,6-Tetra-*O*-acetyl-β-D-glucopyranosyl)-4(5)-phenyl-1*H*-imidazole-2-carboxamide (11a)

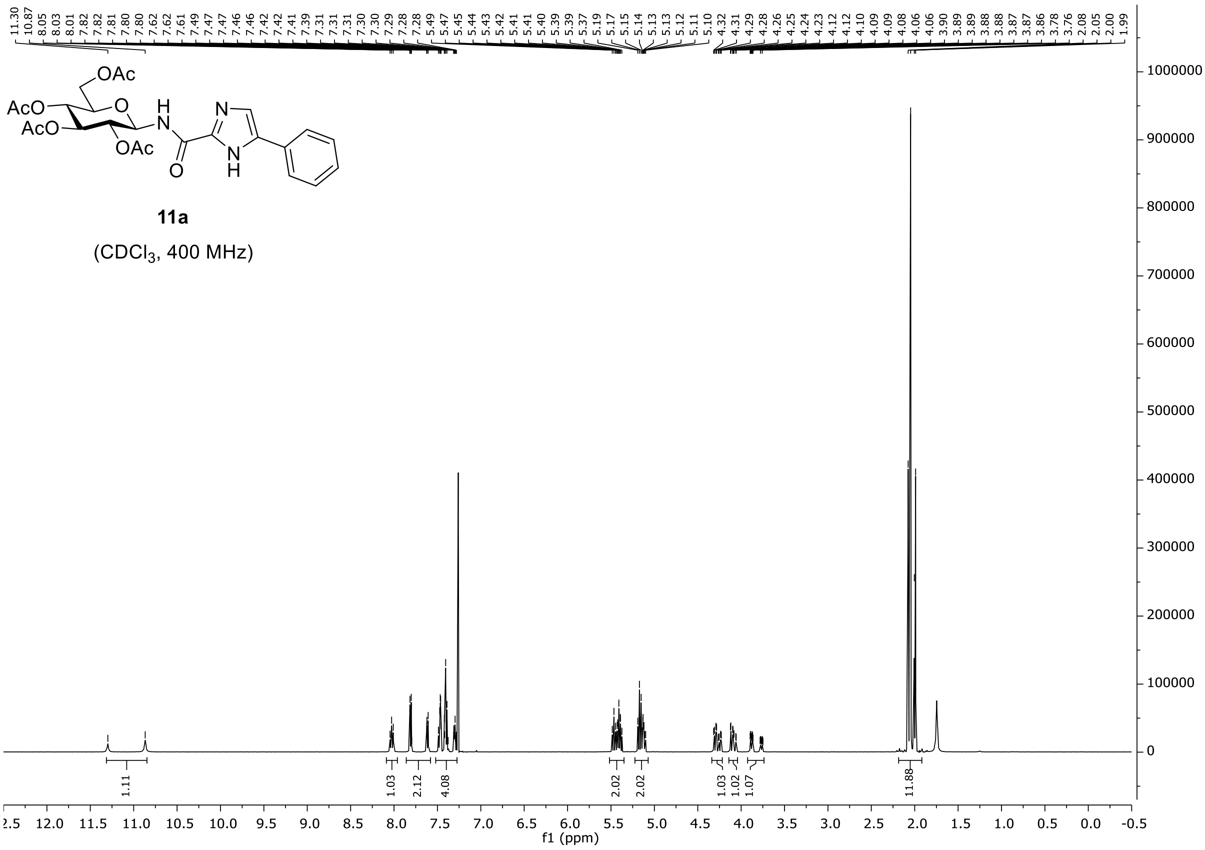

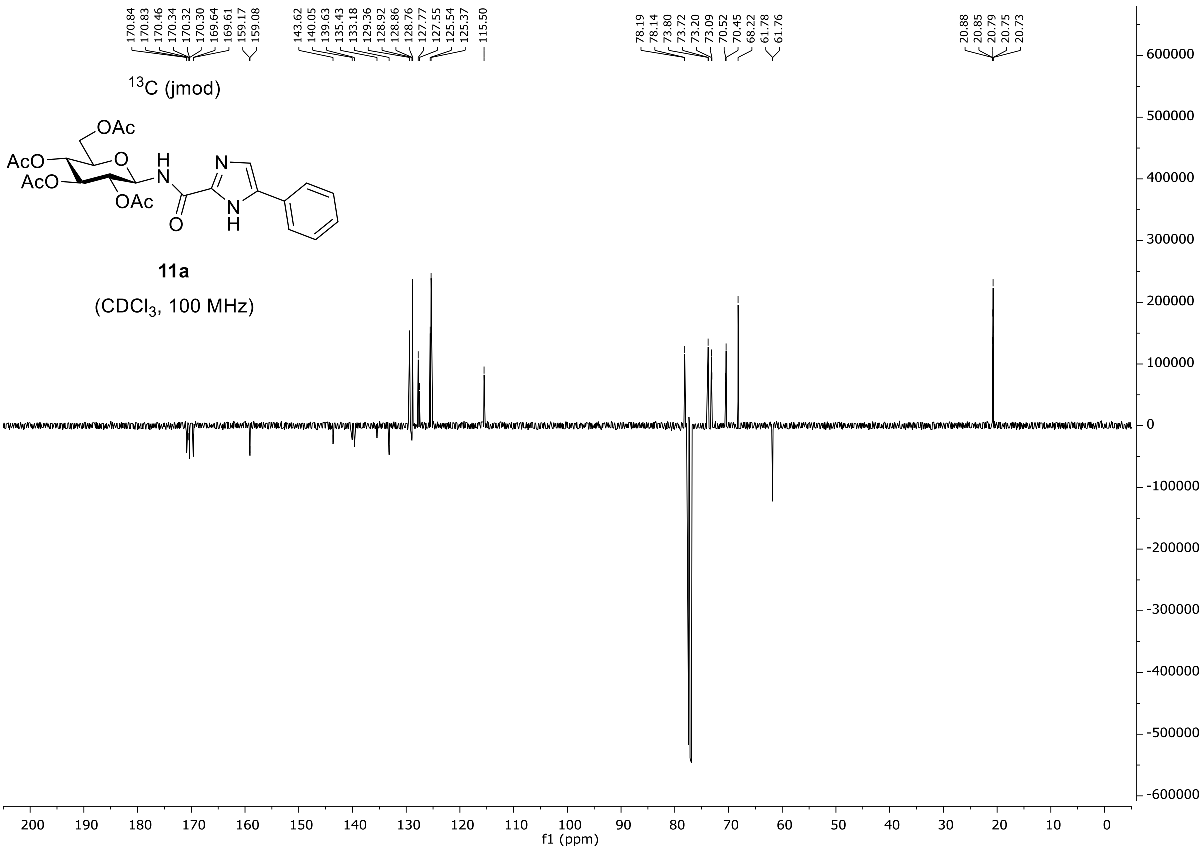

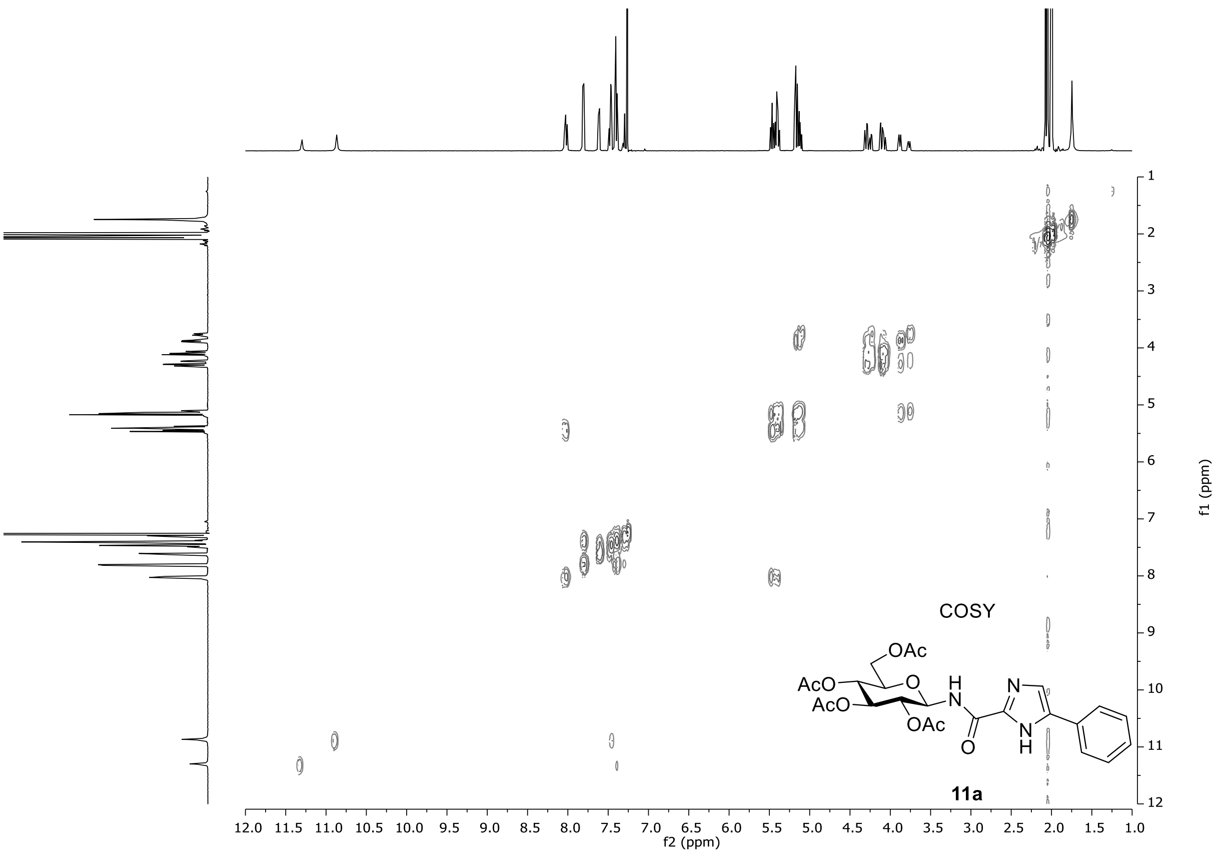
COSY
11a
f2 (ppm)
f1 (ppm)

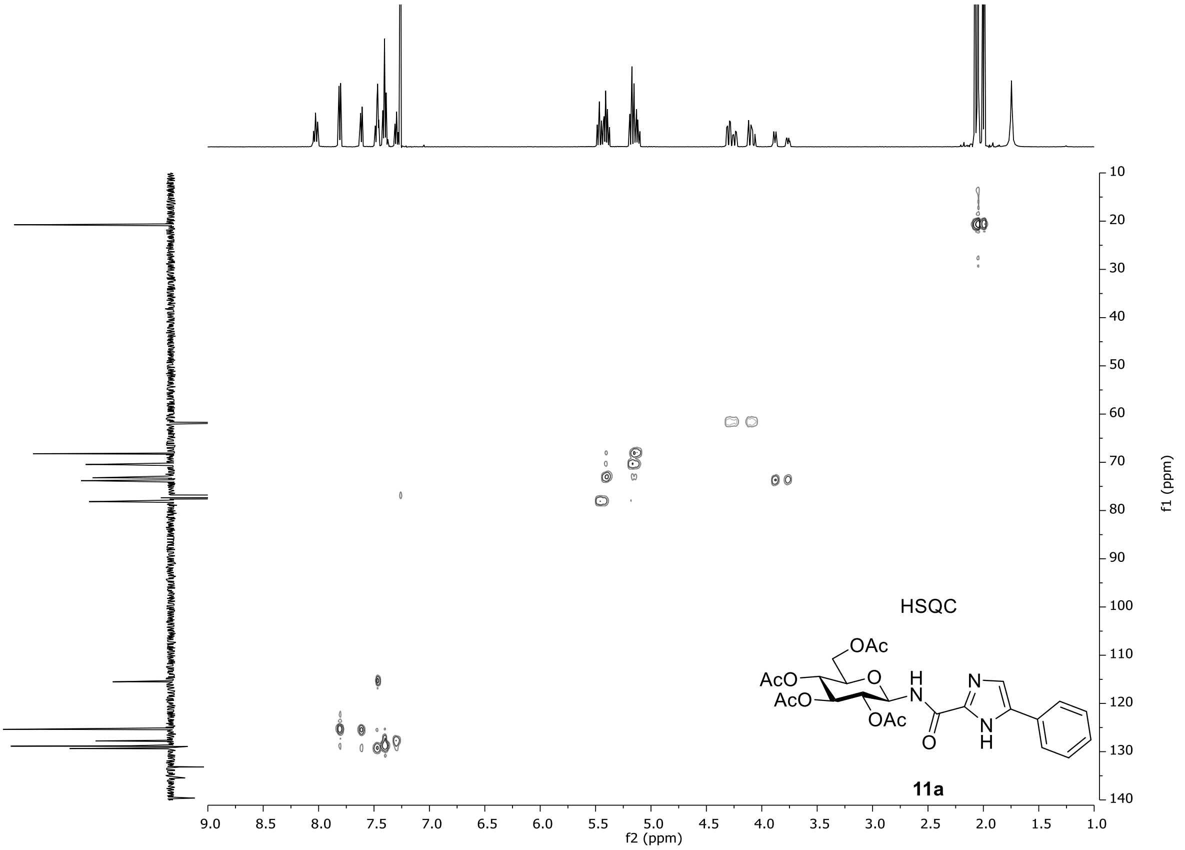
HSQC
11a
f2 (ppm)
f1 (ppm)

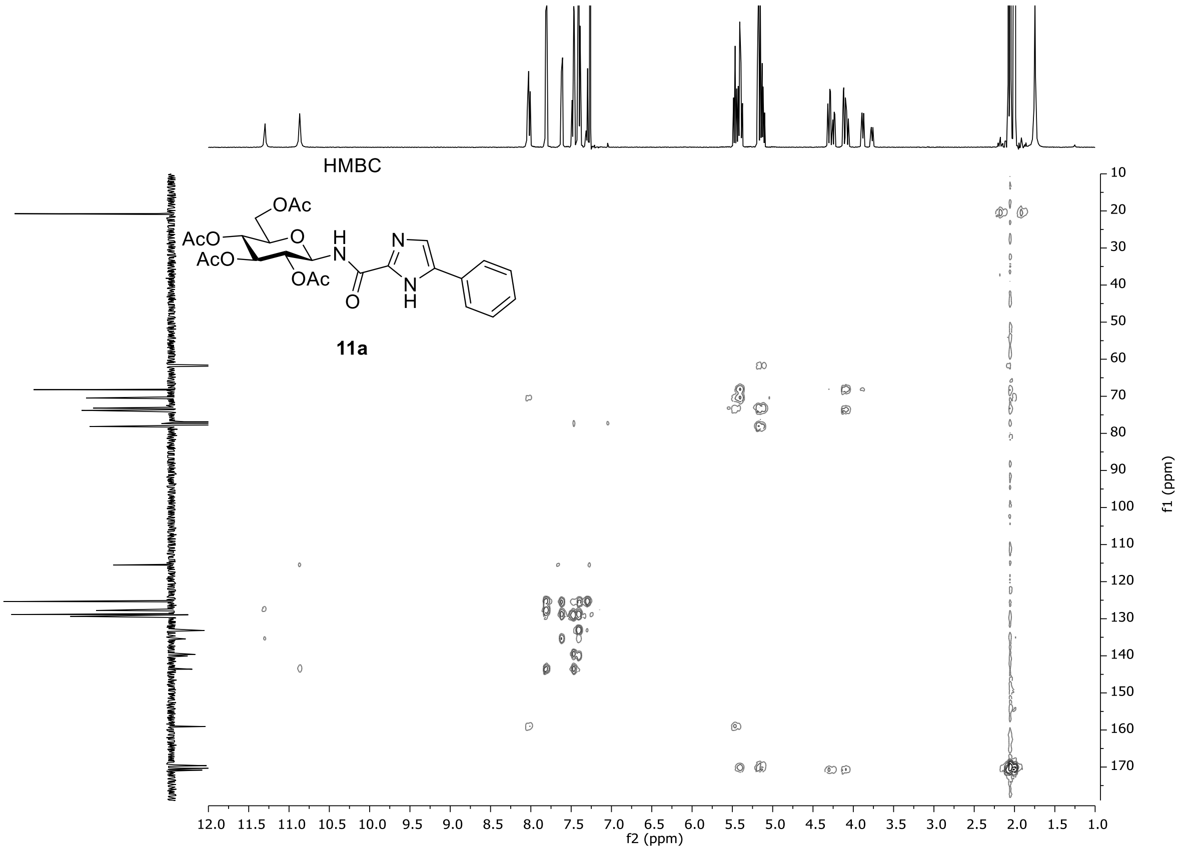
HMBC
11a
f1 (ppm)
f2 (ppm)

***N*-(2,3,4,6-Tetra-*O*-acetyl-β-D-glucopyranosyl)-4(5)-(1-naphthyl)-1*H*-imidazole-2-carboxamide (11b)**

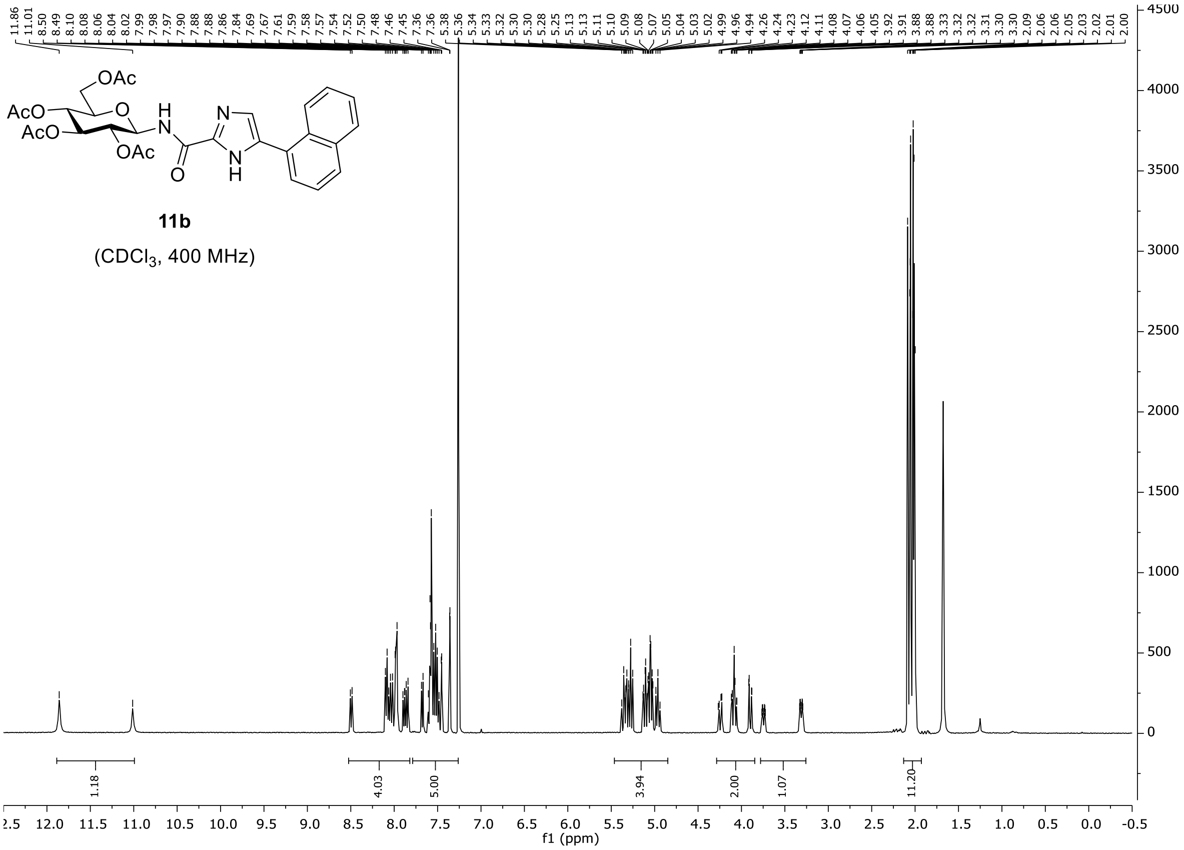

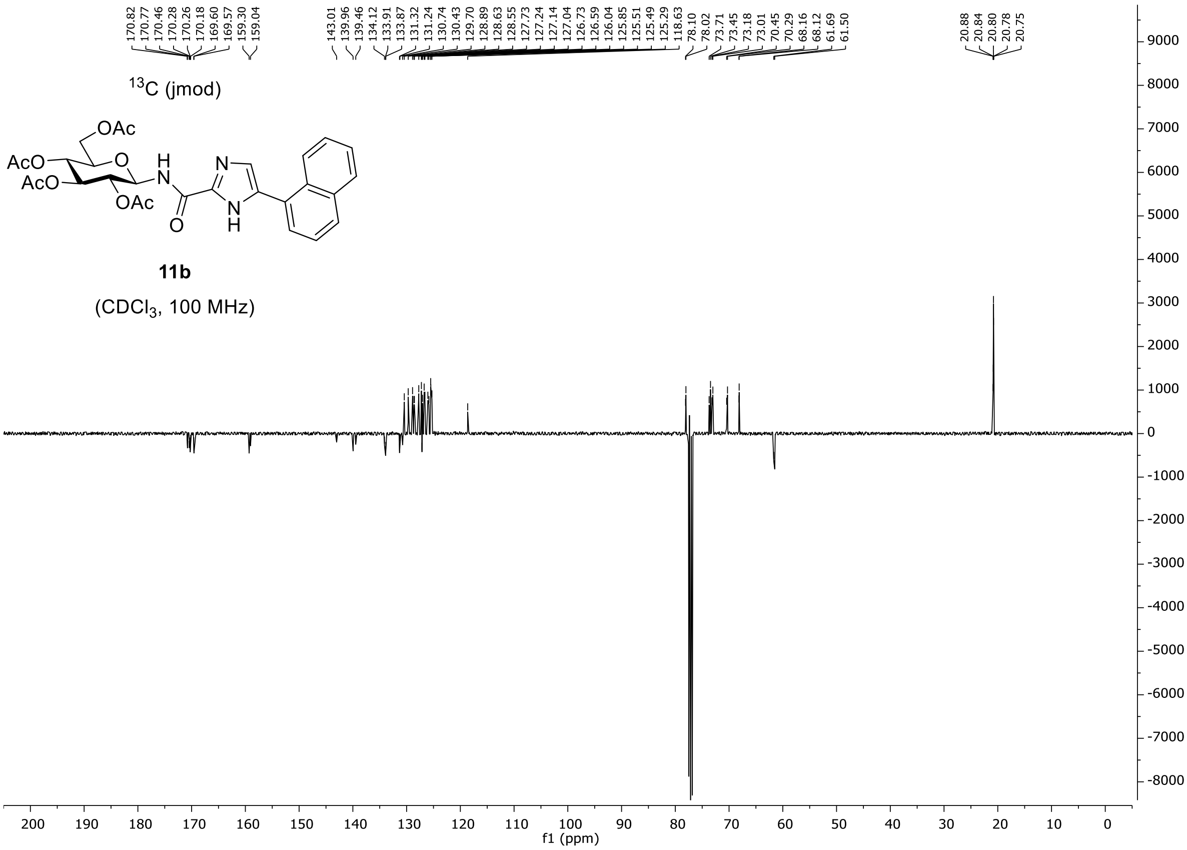

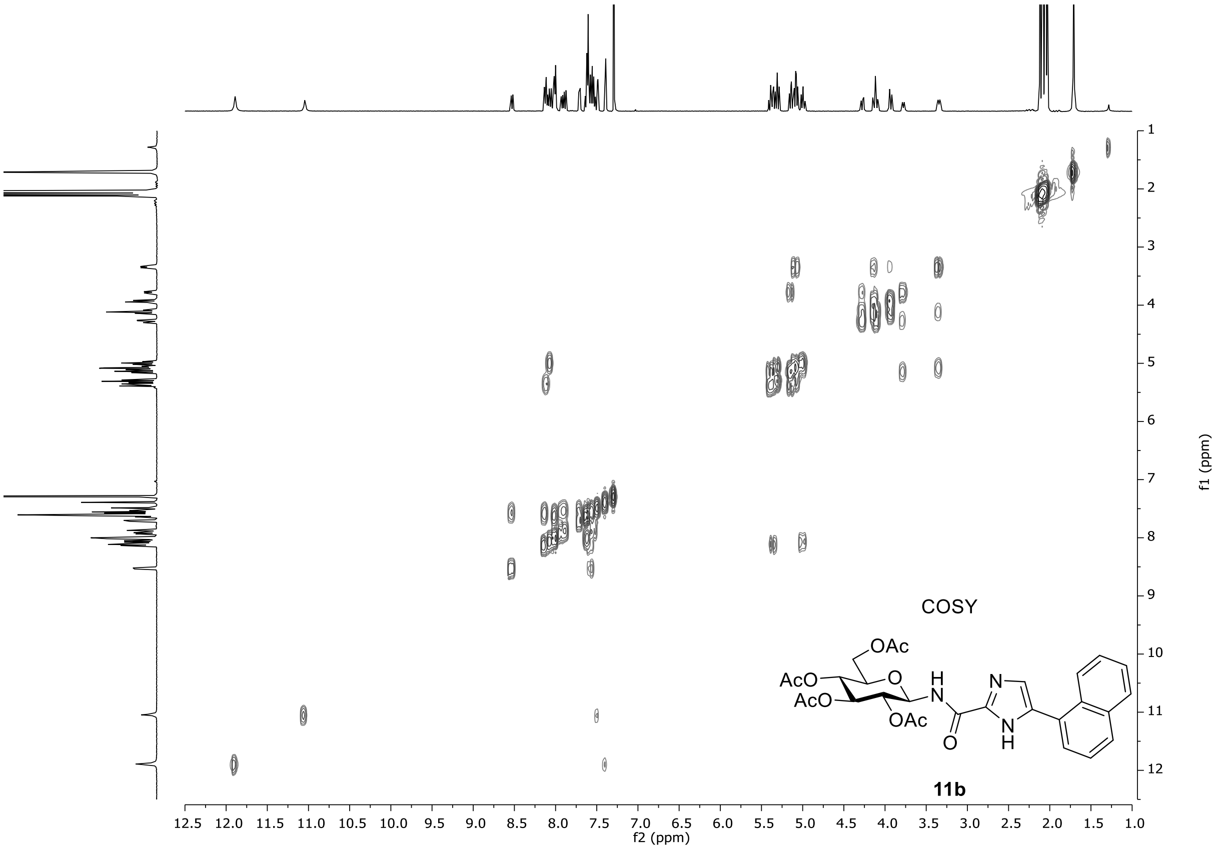
COSY
AcO
AcO
OAc
OAc
O
H
N
N
H
N
O
11b
f1 (ppm)
f2 (ppm)

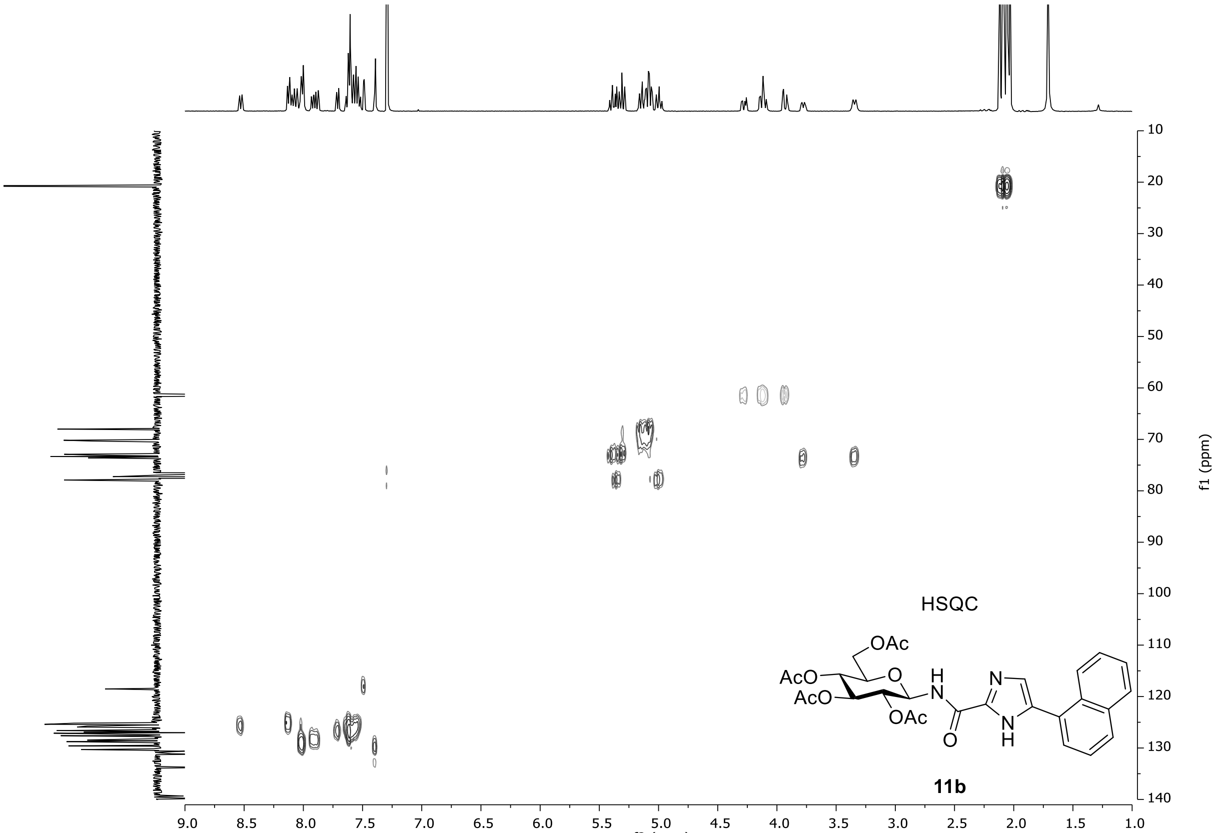
HSQC
AcO
AcO
OAc
OAc
O
H
N
N
H
N
O
11b
f1 (ppm)
f2 (ppm)

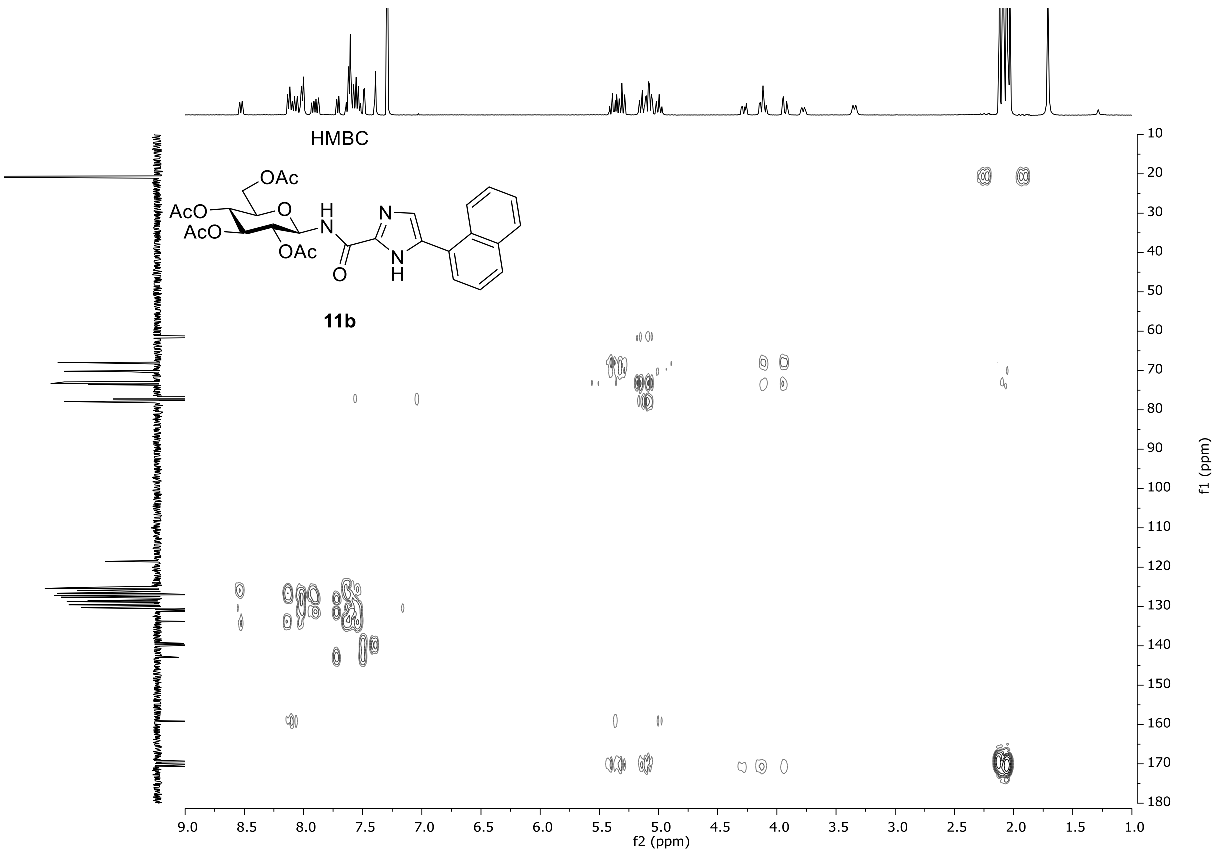

11b

N-(2,3,4,6-Tetra-*O*-acetyl-β-D-glucopyranosyl)-4(5)-(2-naphthyl)-1*H*-imidazole-2-carboxamide (11c)

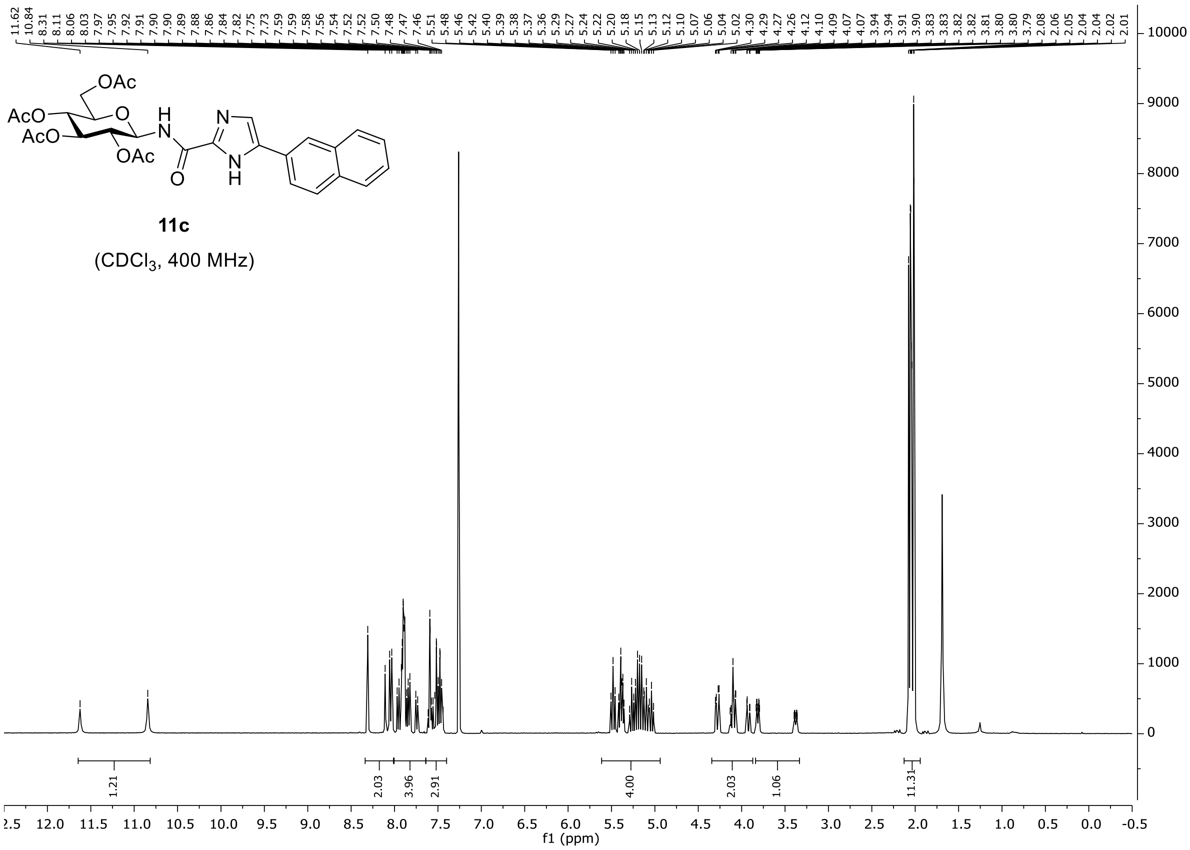

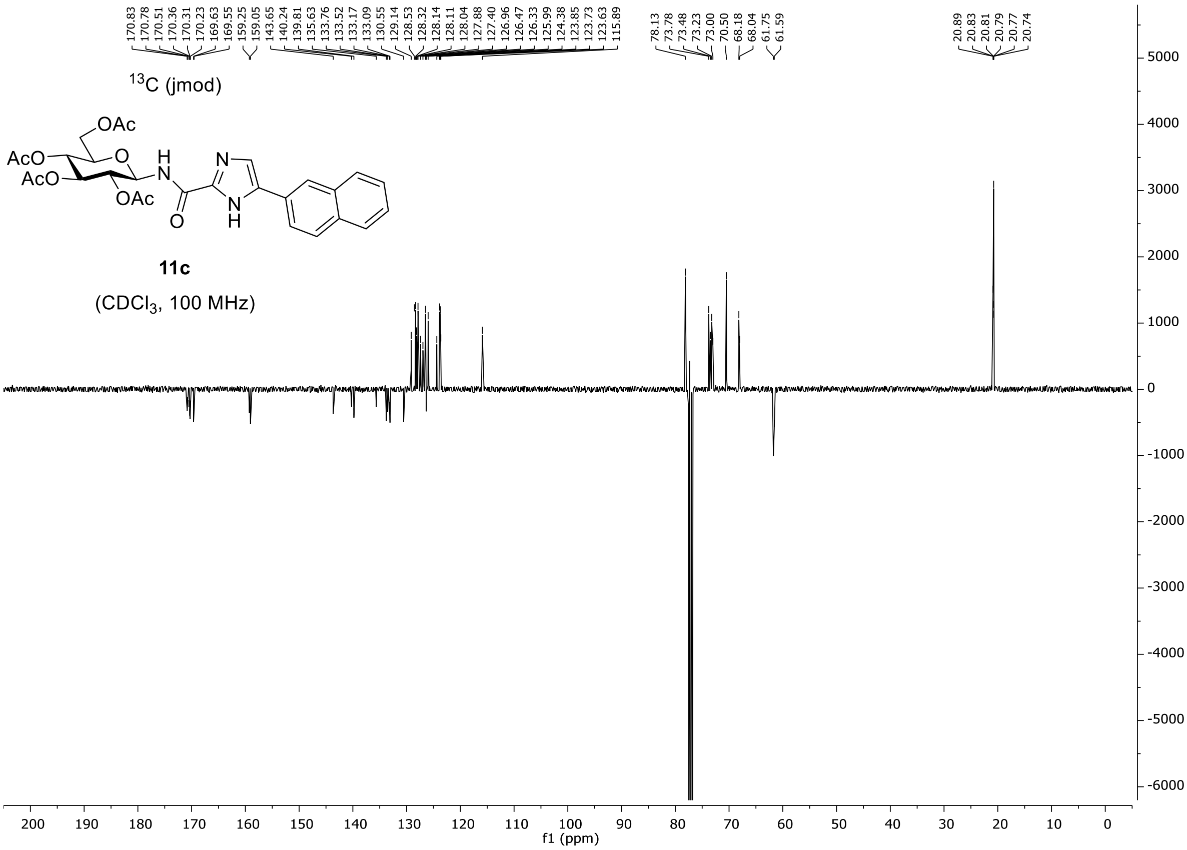

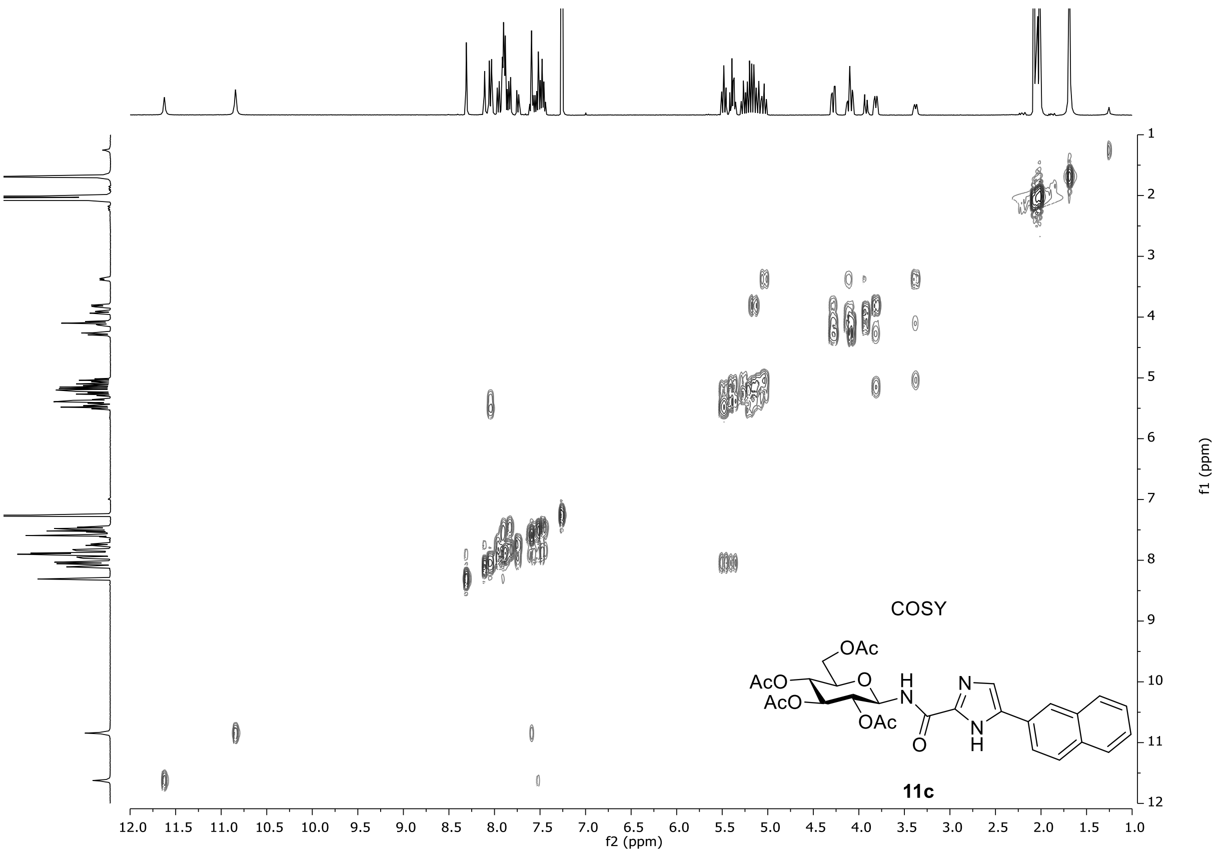
COSY
OAc
AcO
AcO
O
H
N
N
OAc
O
N
H
11c
f1 (ppm)
f2 (ppm)

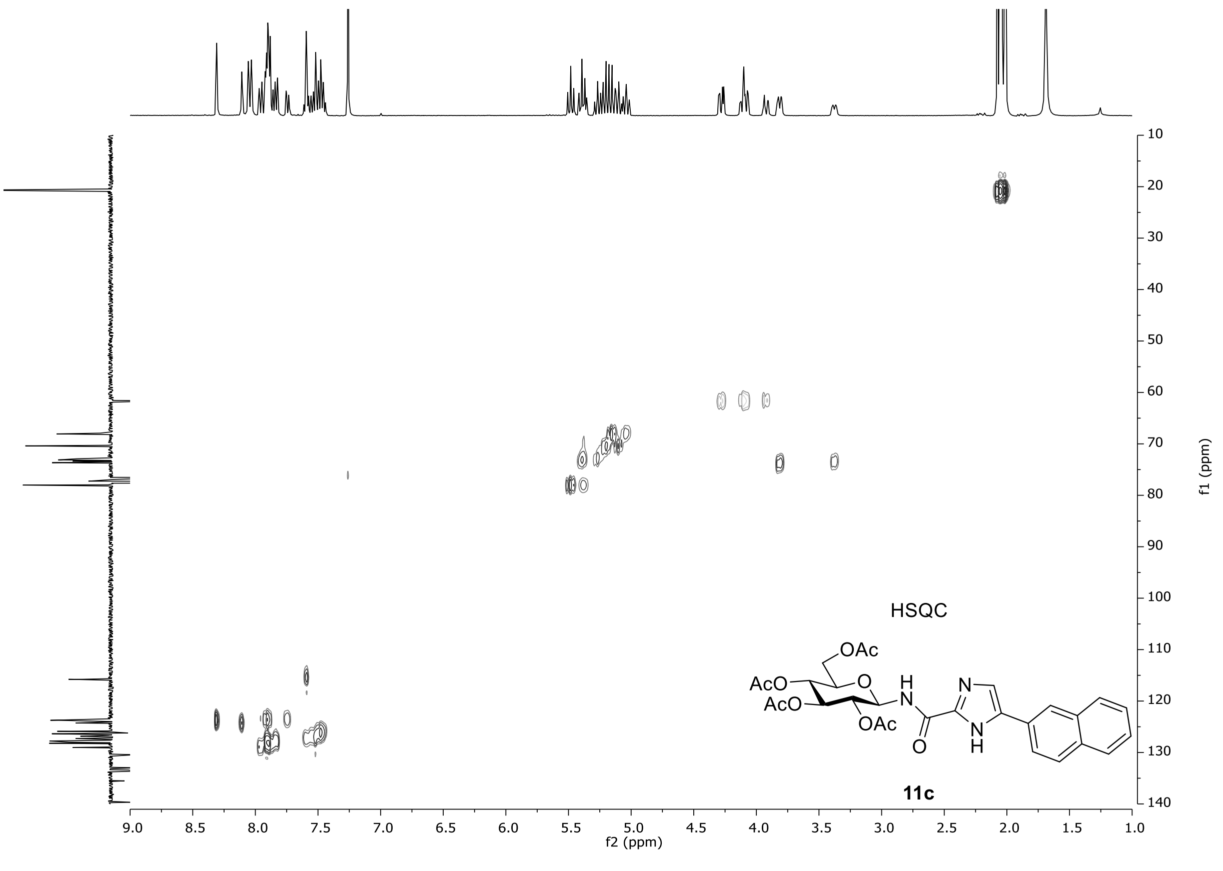
HSQC
OAc
AcO
AcO
O
H
N
N
OAc
O
N
H
11c
f1 (ppm)
f2 (ppm)

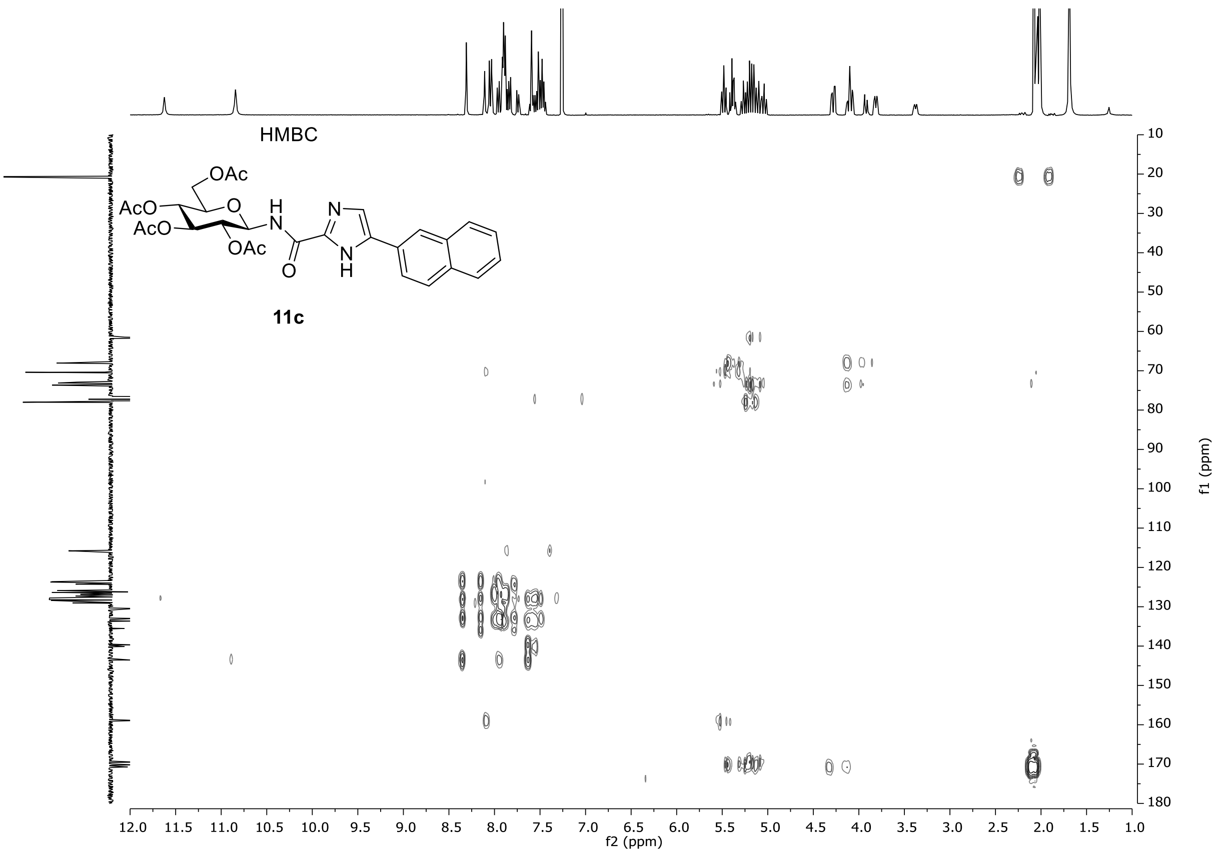
HMBC
11c
f2 (ppm)
f1 (ppm)

N-(β-D-Glucopyranosyl)-2-phenyl-1*H*-imidazole-4(5)-carboxamide (1a)

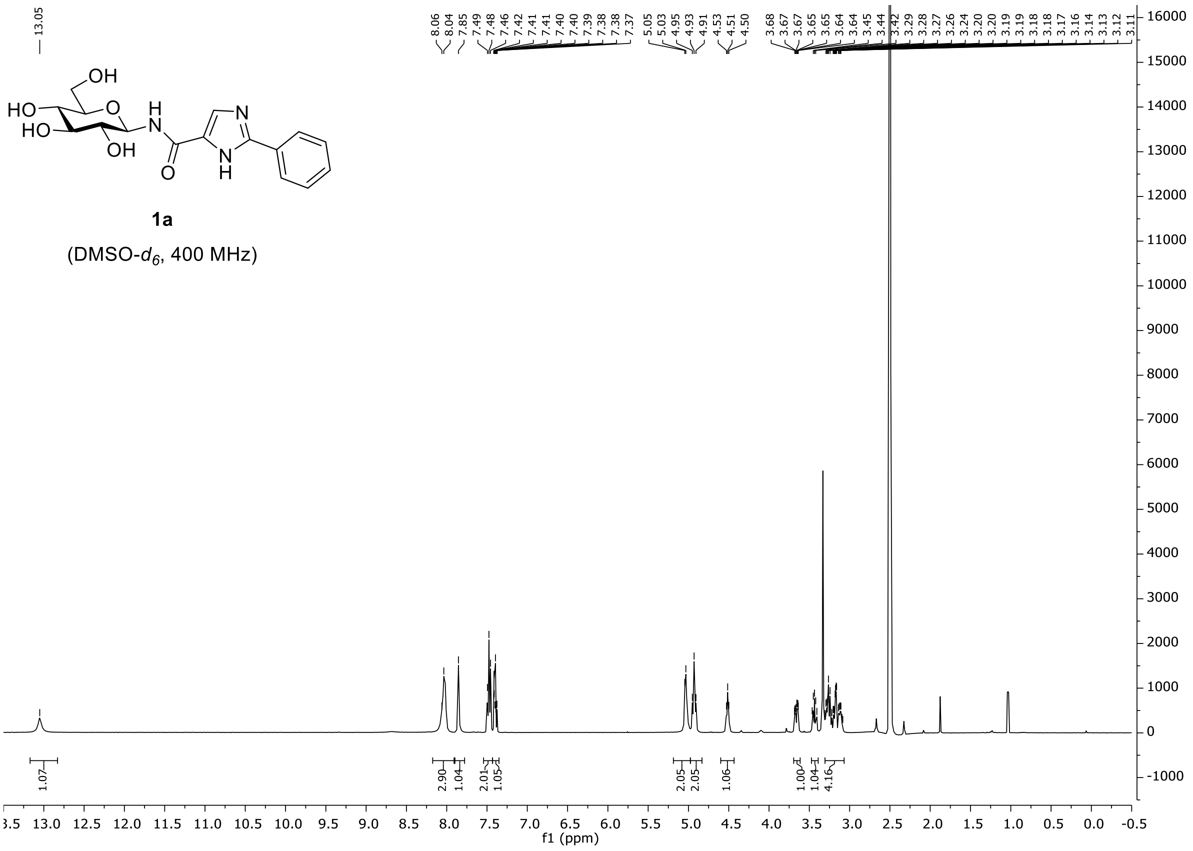

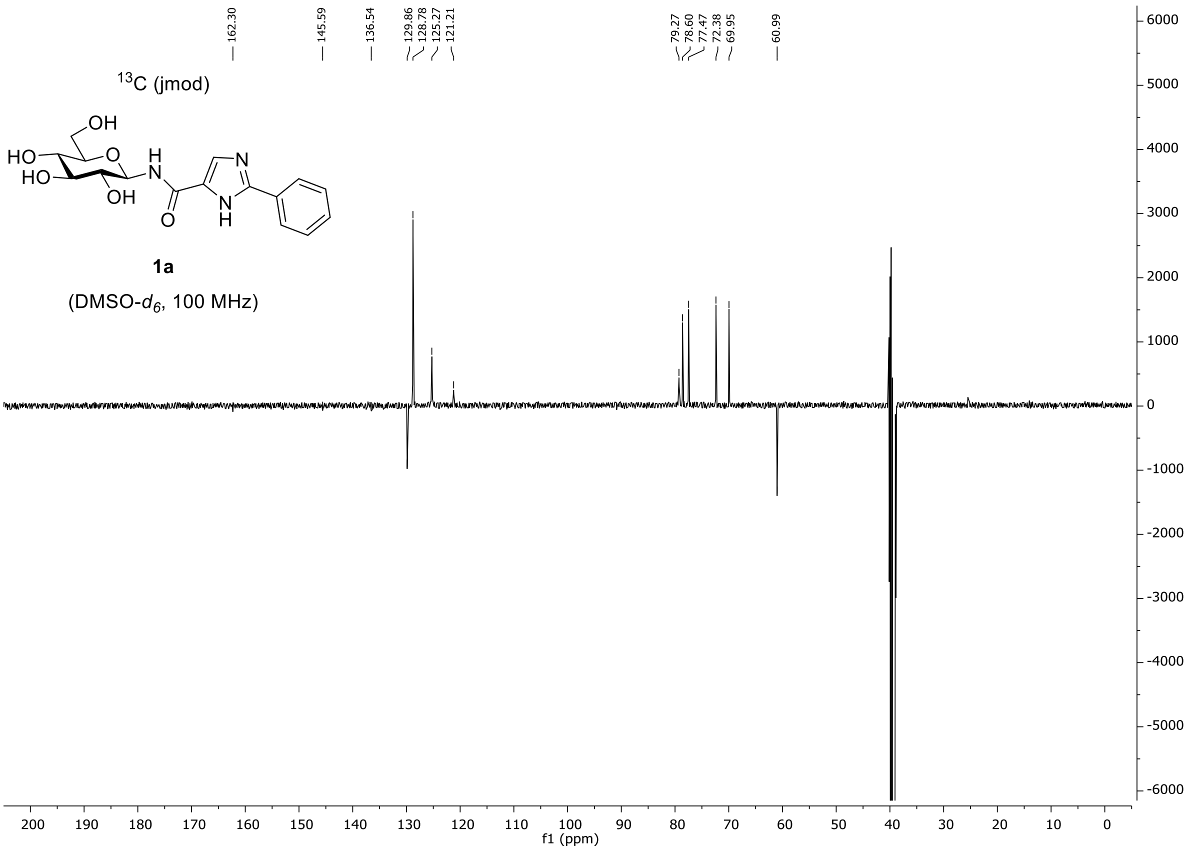

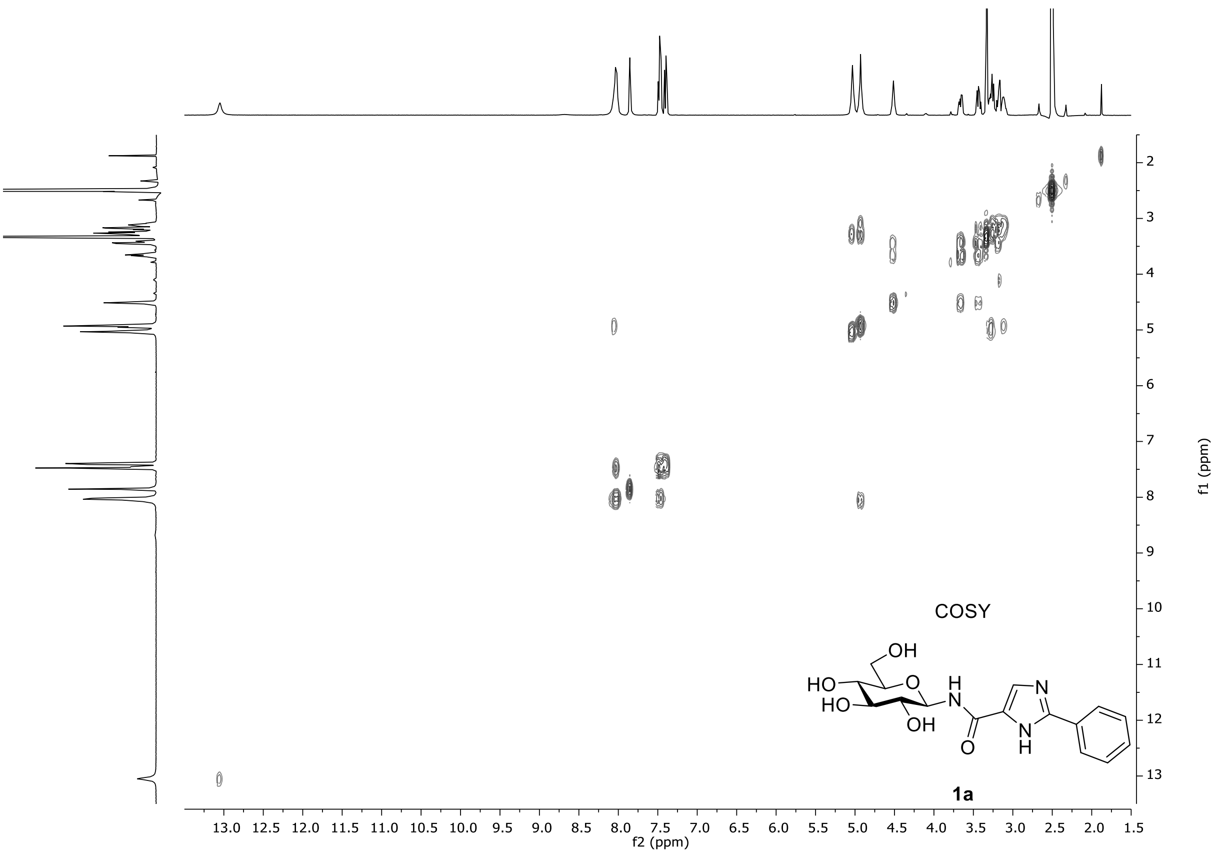
COSY
1a
f2 (ppm)
f1 (ppm)

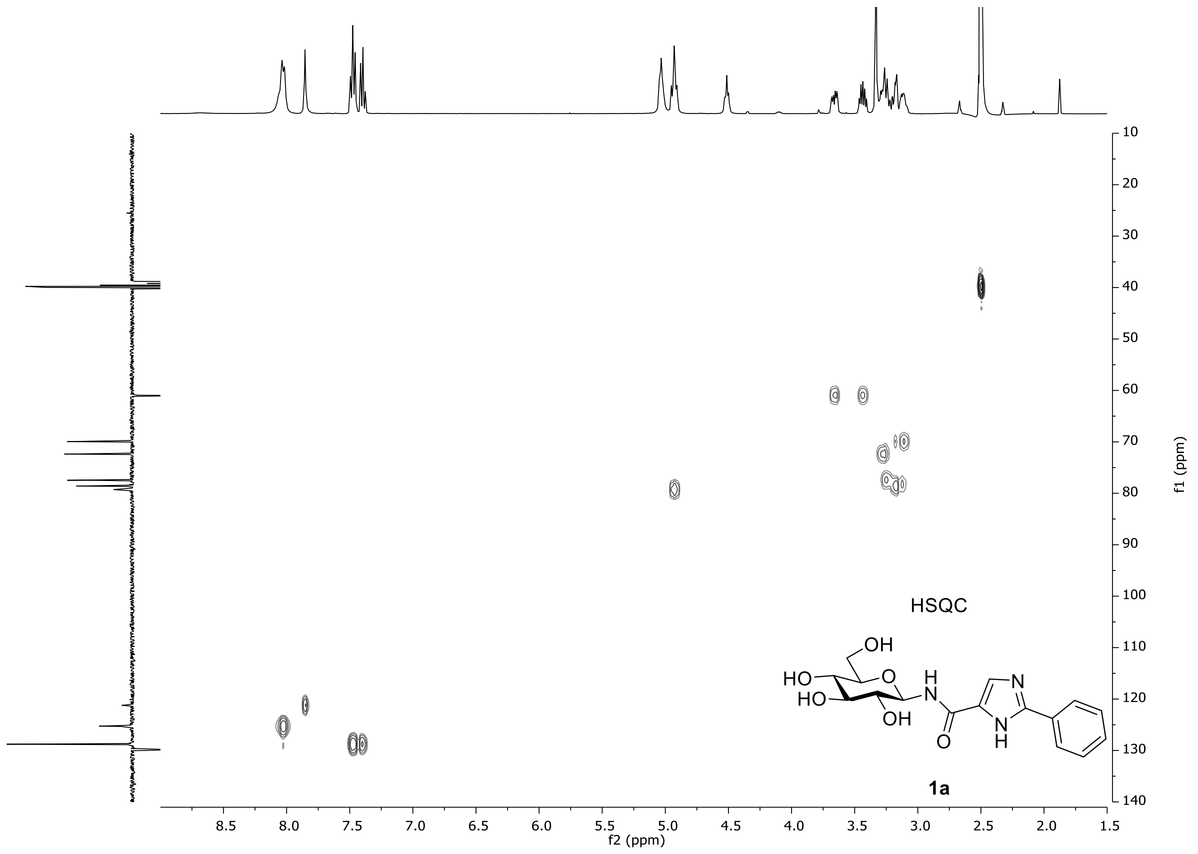
HSQC
1a
f2 (ppm)
f1 (ppm)

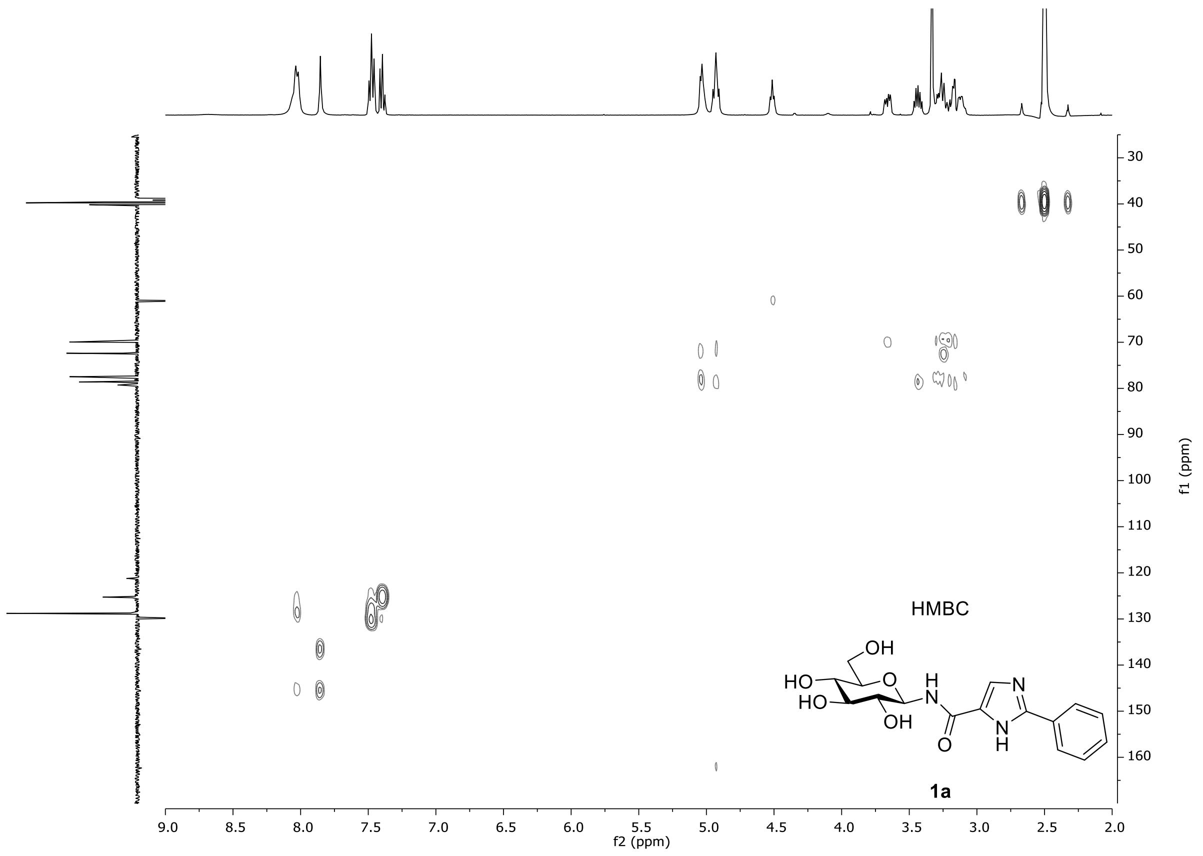
HMBC
1a
f2 (ppm)
f1 (ppm)

N-(β-D-Glucopyranosyl)-2-(1-naphthyl)-1*H*-imidazole-4(5)-carboxamide (1b)

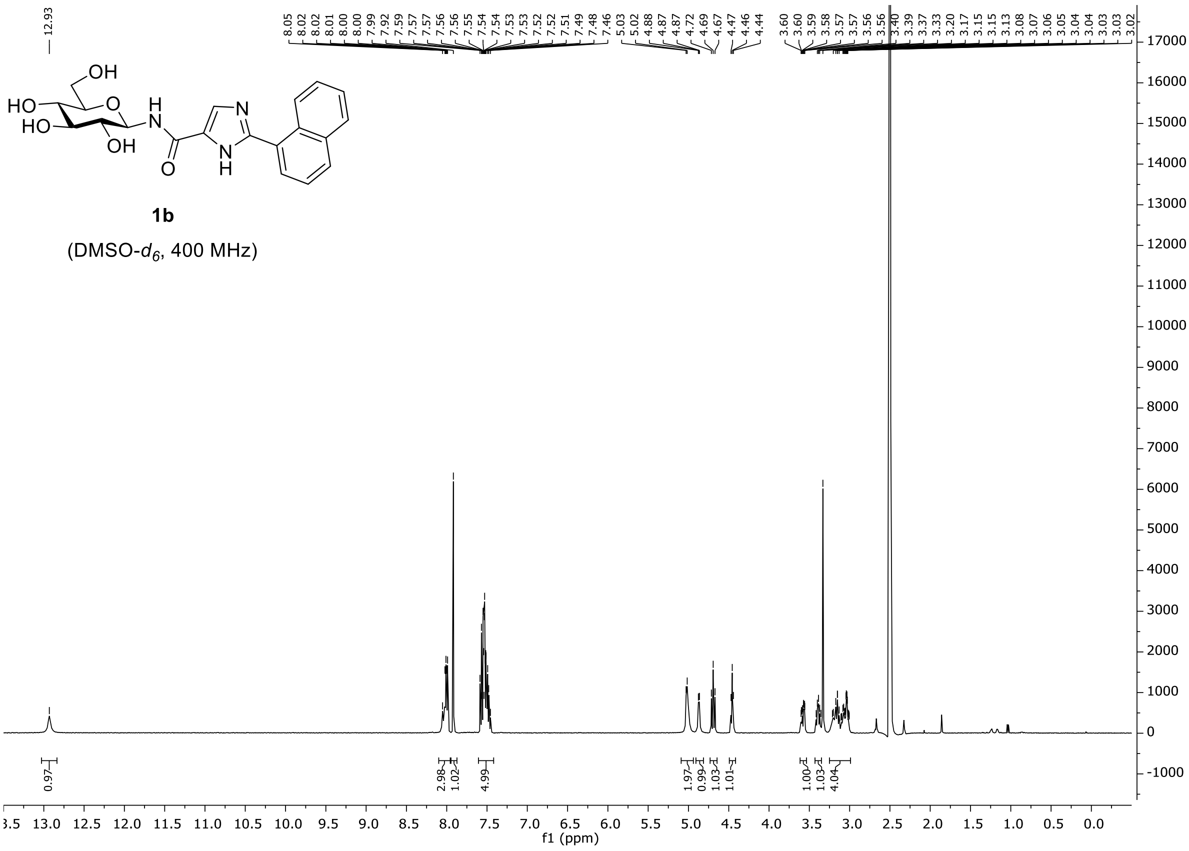

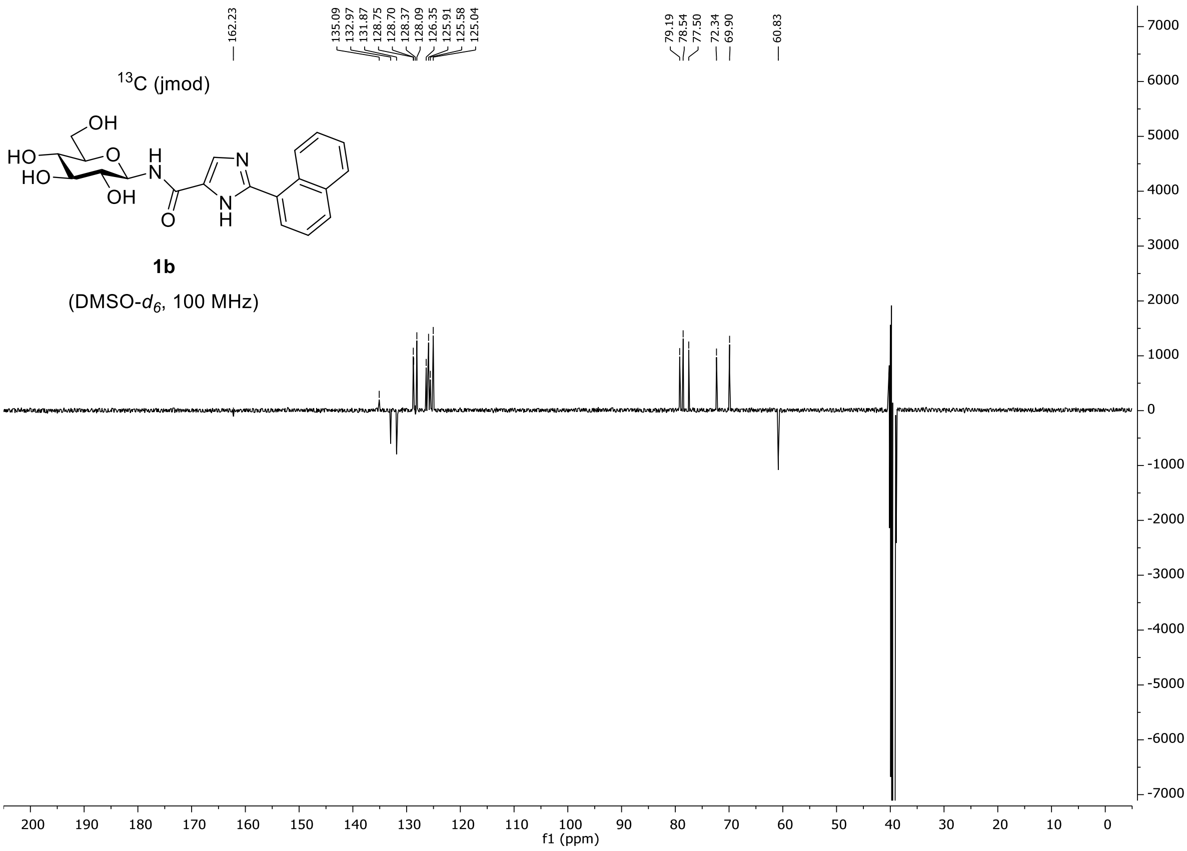

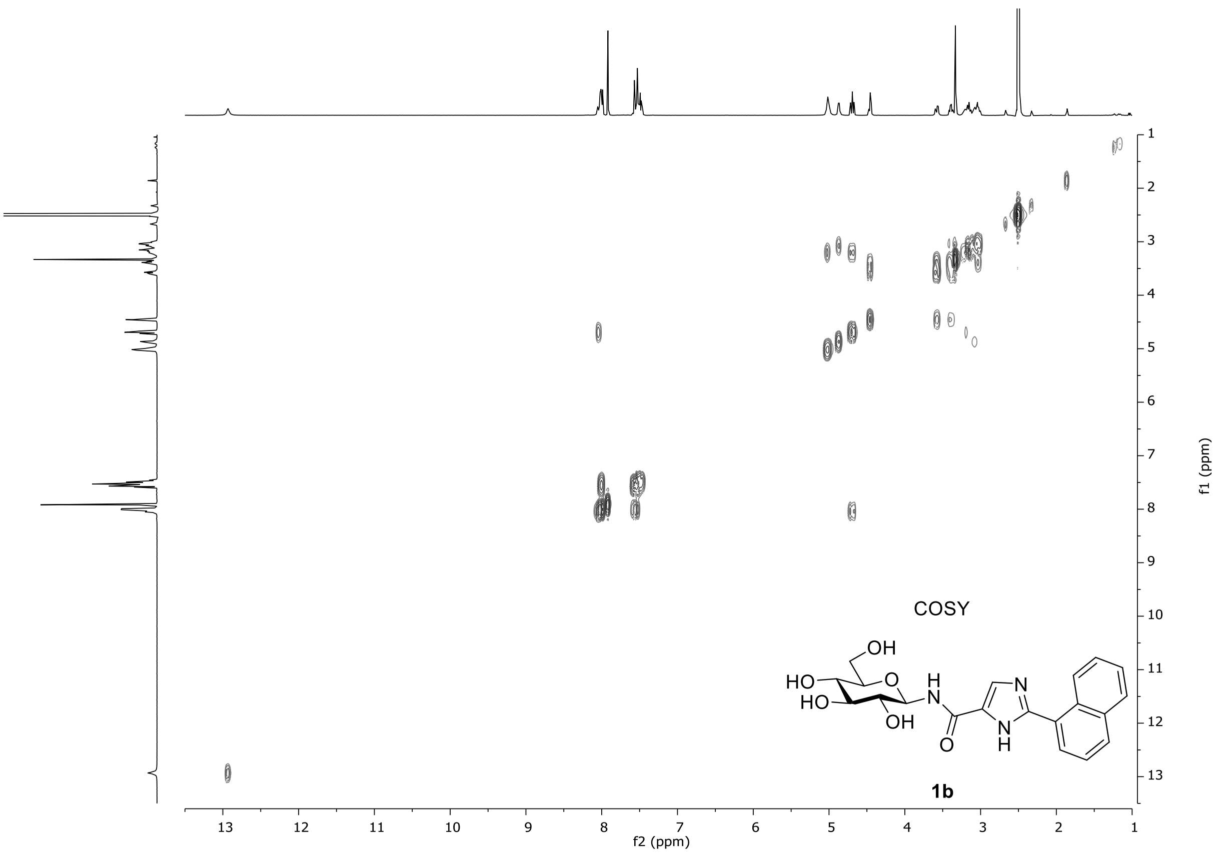
COSY
f2 (ppm)
f1 (ppm)
1b

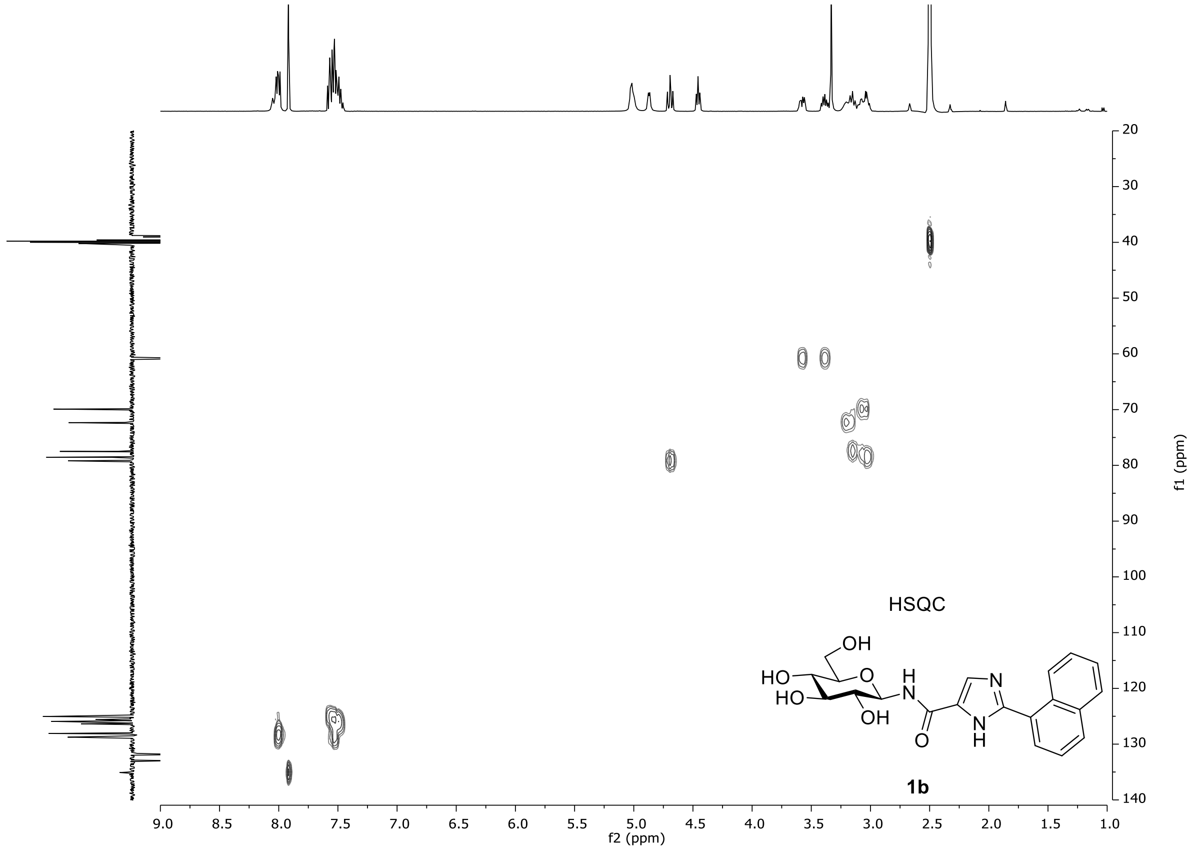
HSQC
f2 (ppm)
f1 (ppm)
1b

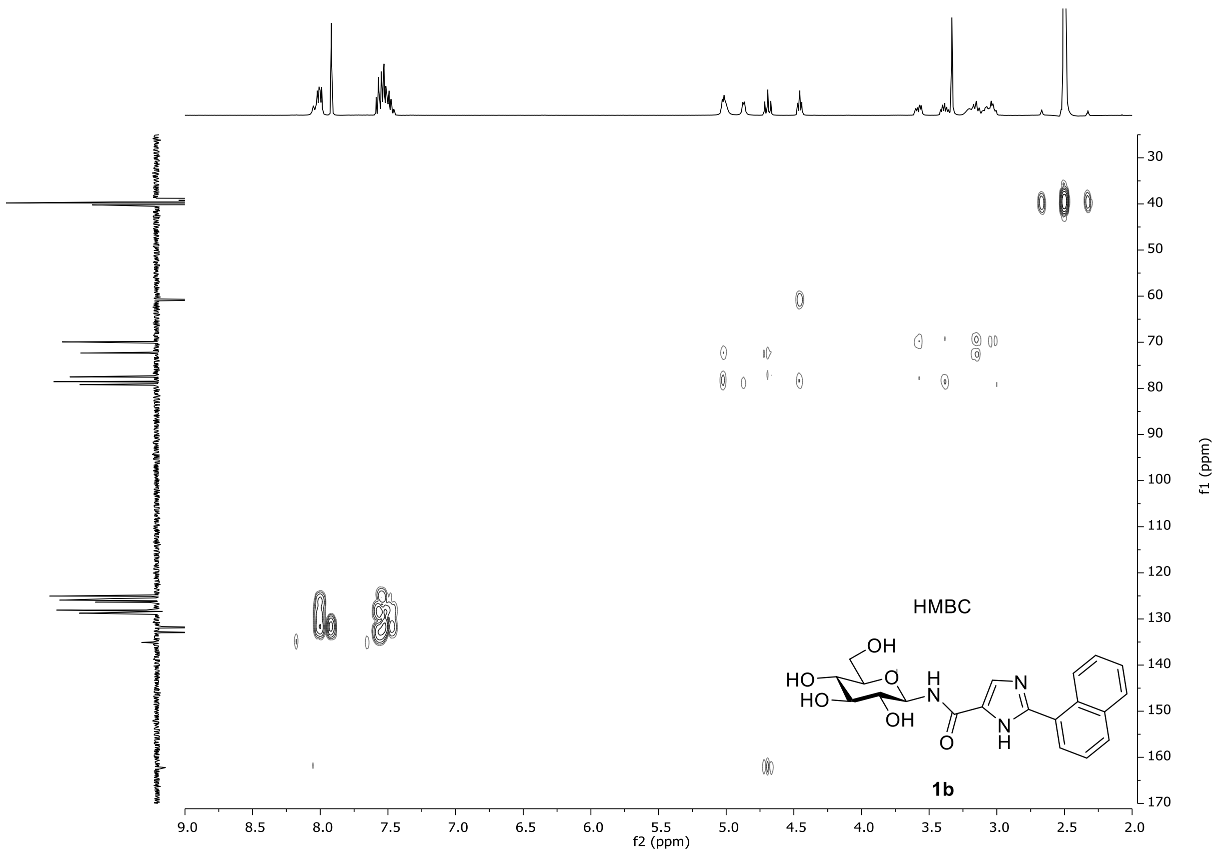
HMBC
1b
f1 (ppm)
f2 (ppm)

N-(β-D-Glucopyranosyl)-2-(2-naphthyl)-1*H*-imidazole-4(5)-carboxamide (1c)

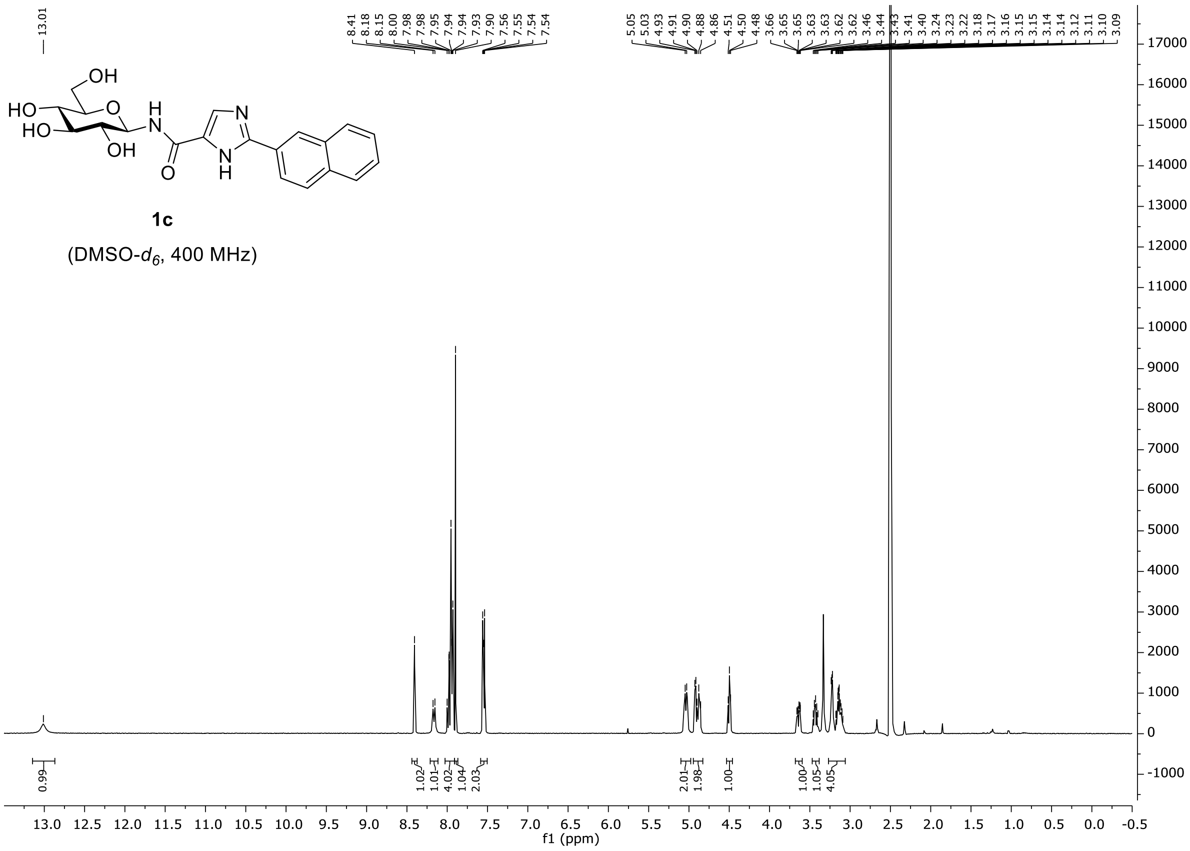

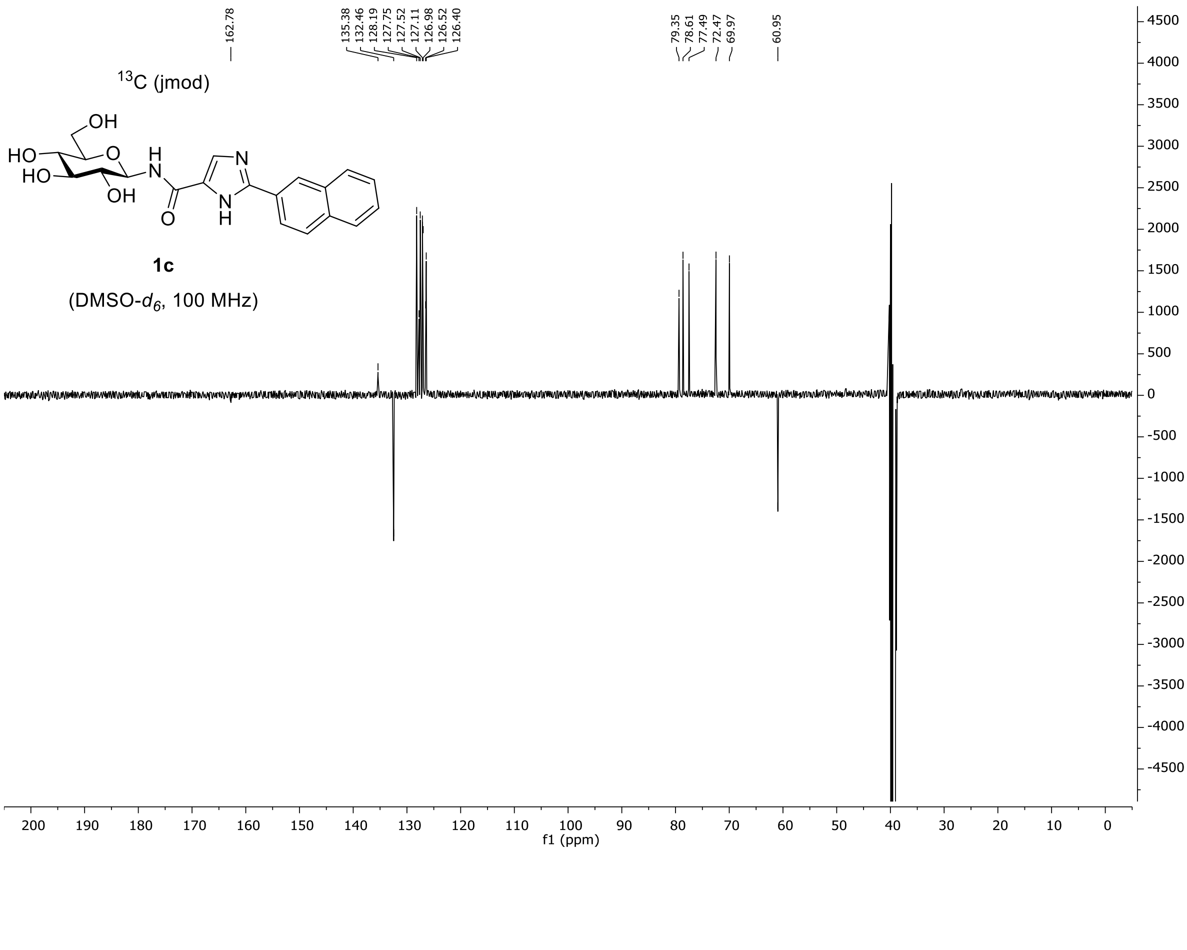

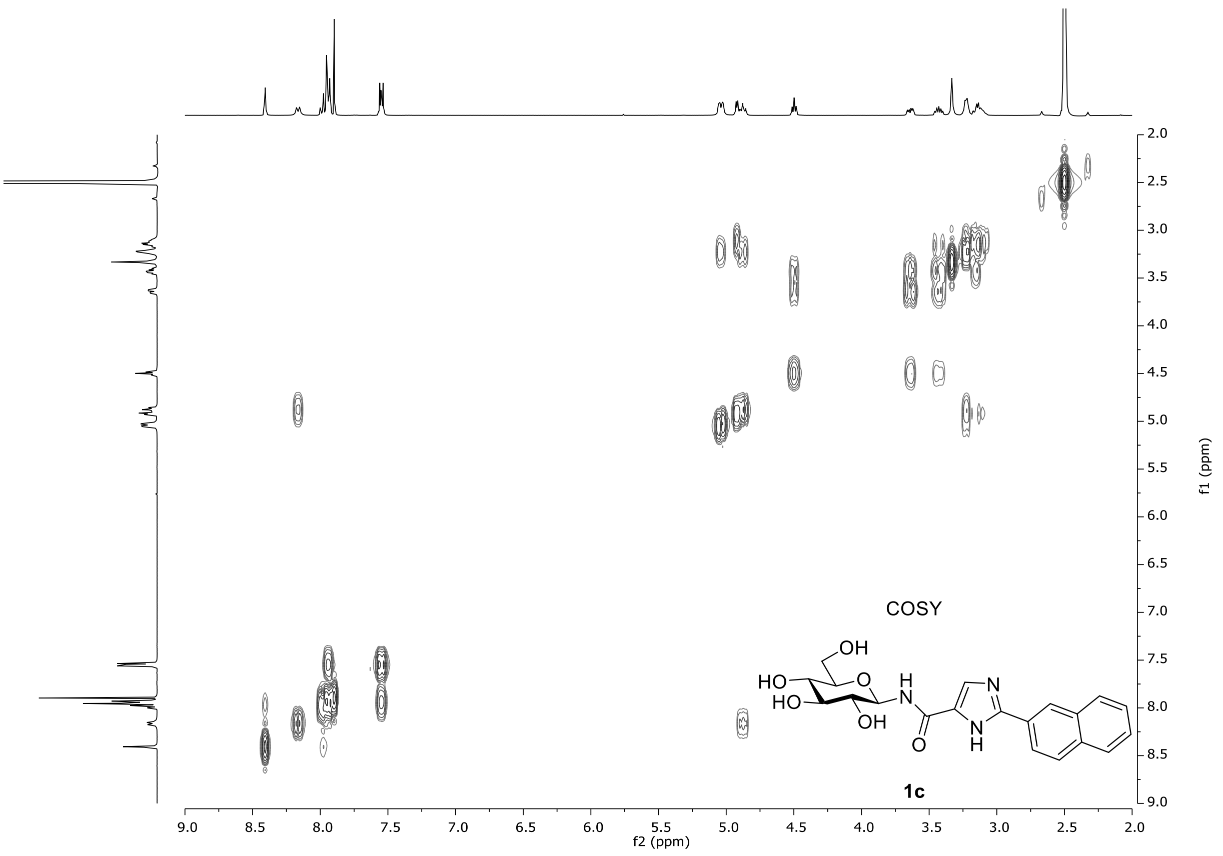
COSY
HO
HO
OH
OH
H
N
O
N
H
N
1c
f1 (ppm)
f2 (ppm)

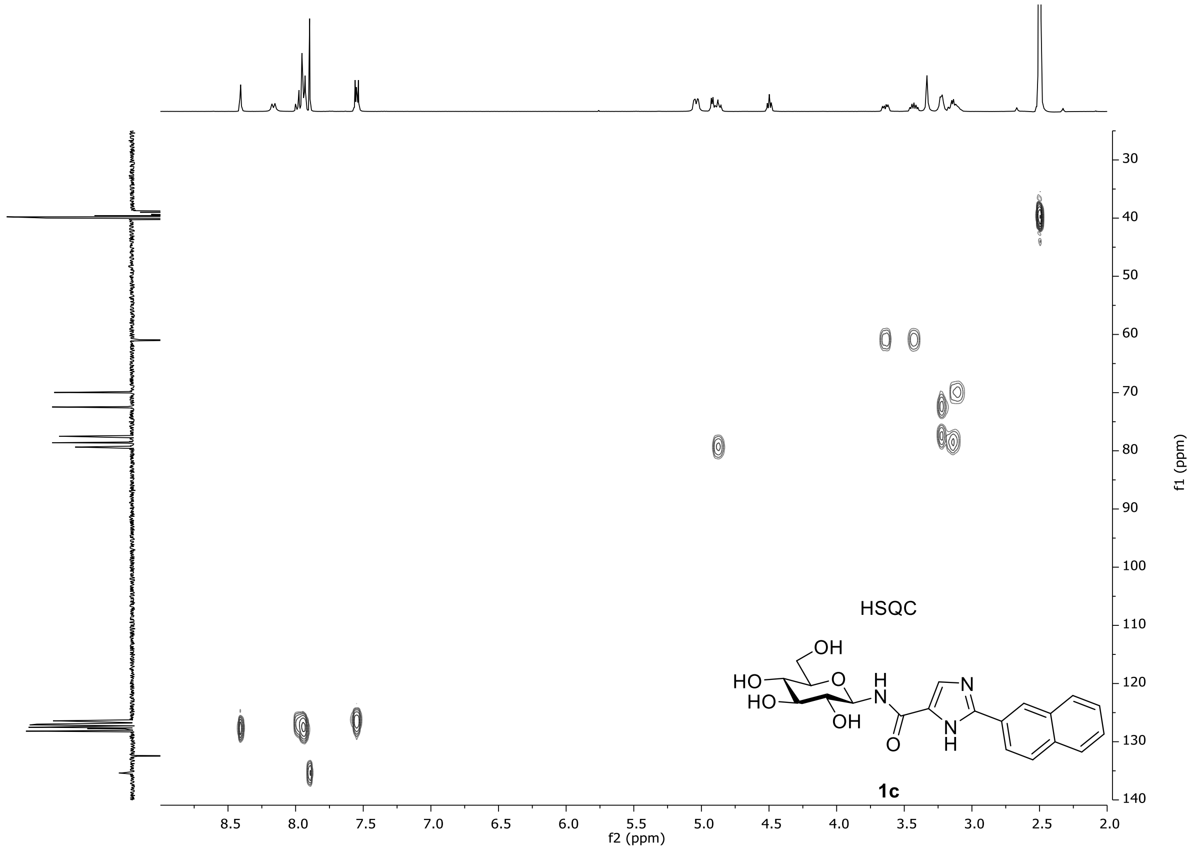
HSQC
HO
HO
OH
OH
H
N
O
N
H
N
1c
f1 (ppm)
f2 (ppm)

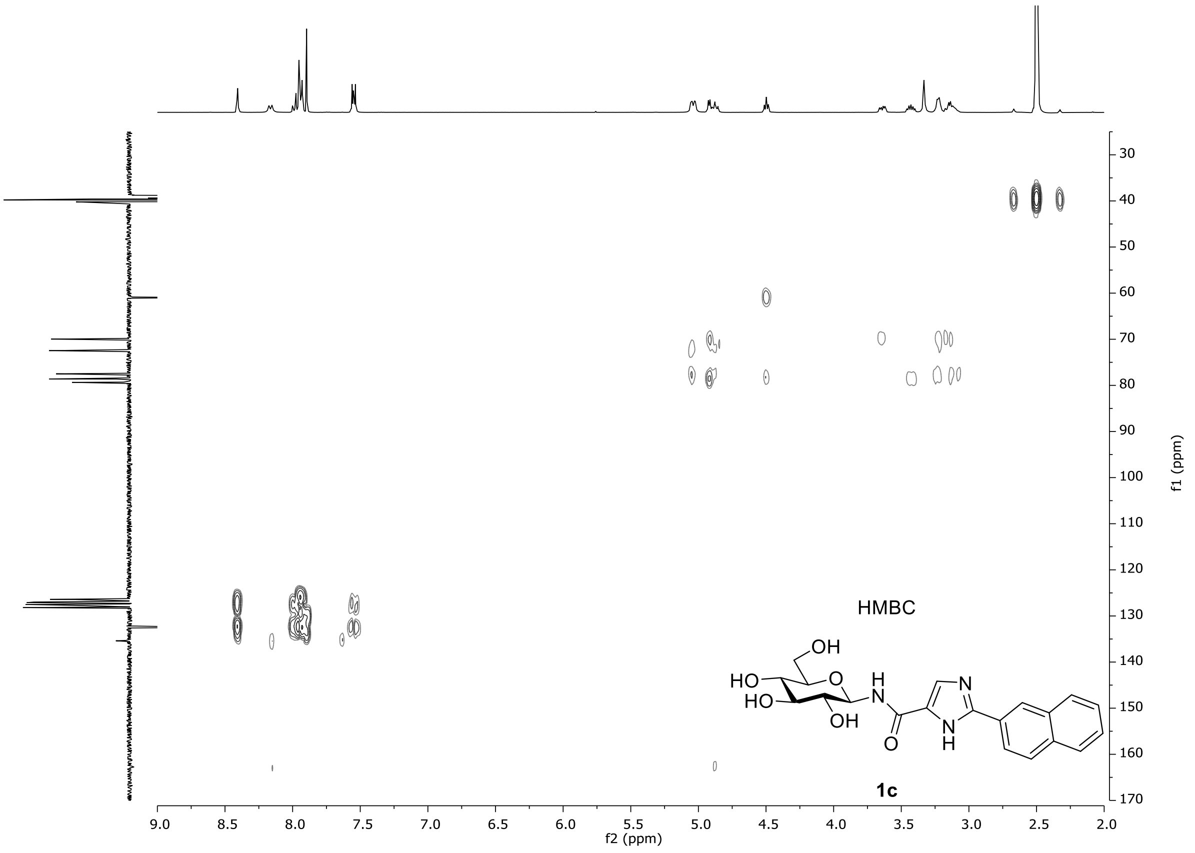
HMBC
1c
f2 (ppm)
f1 (ppm)

N-(β-D-Glucopyranosyl)-4(5)-phenyl-1*H*-imidazole-2-carboxamide (2a)

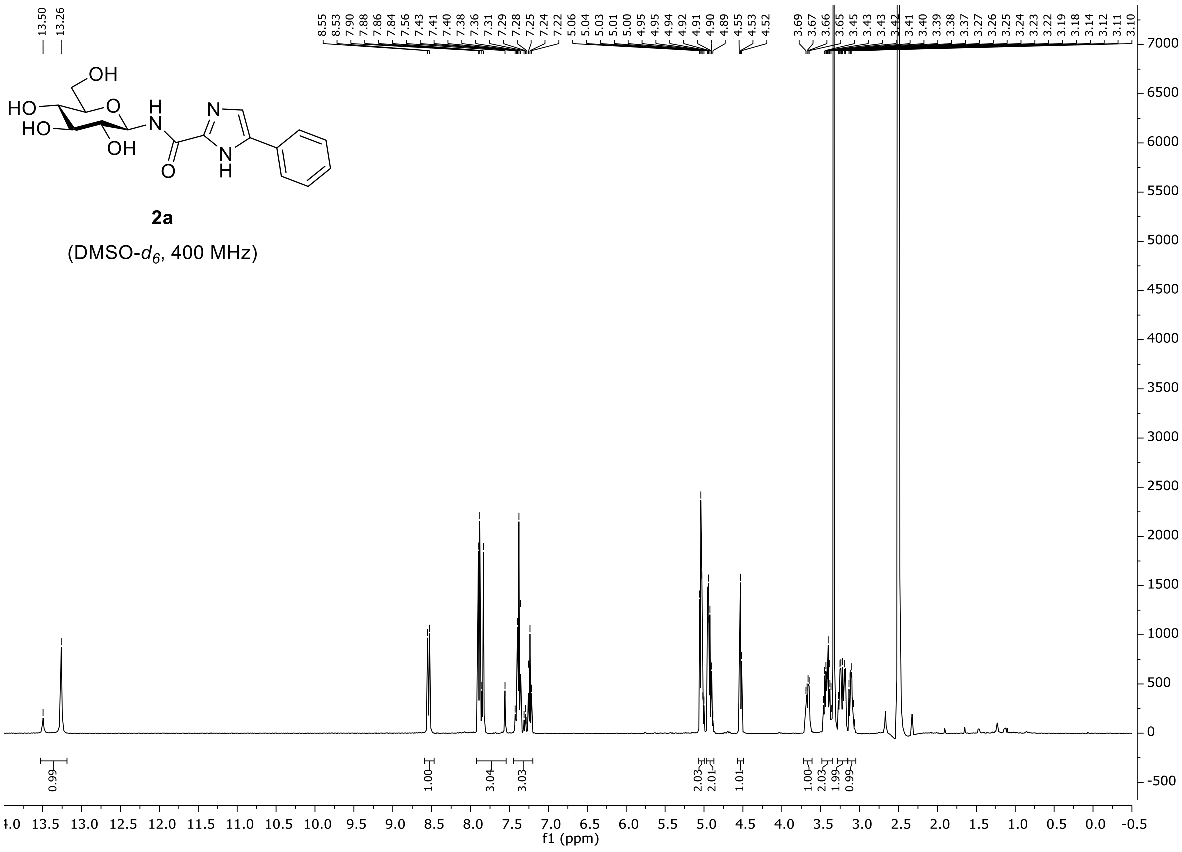

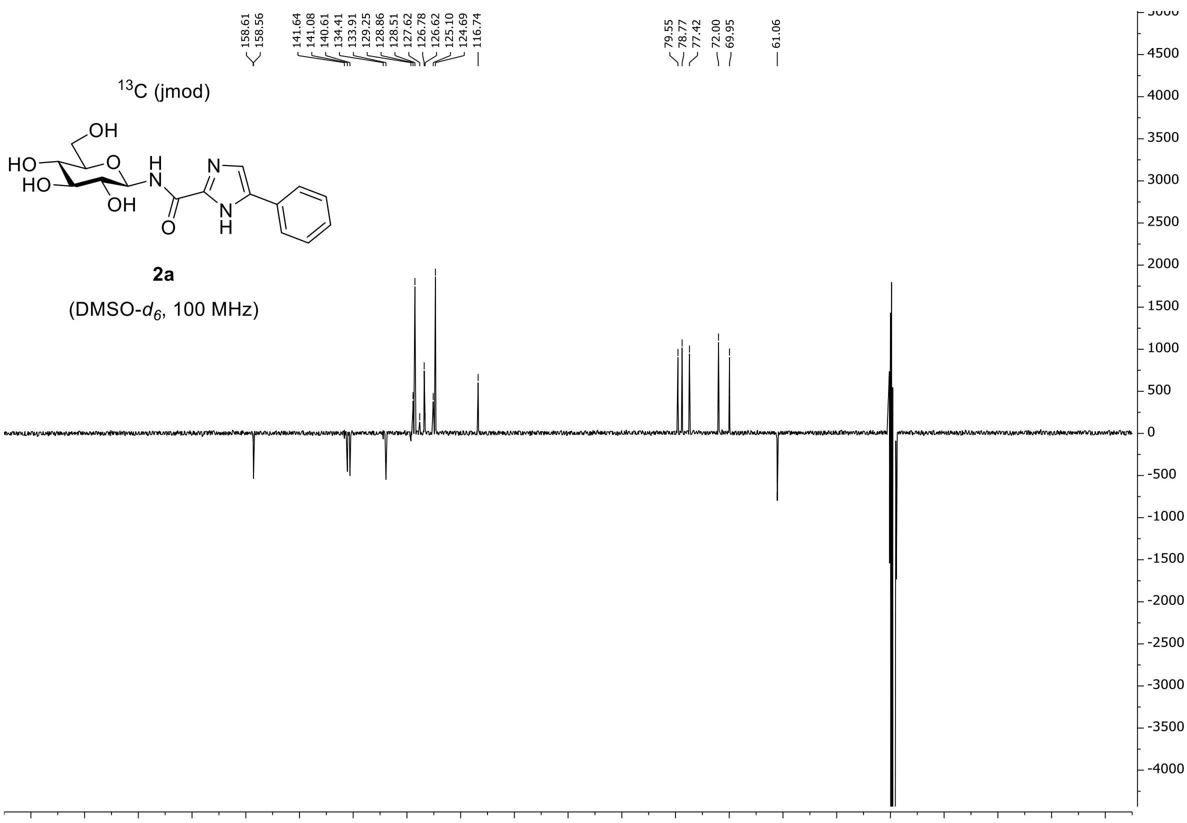

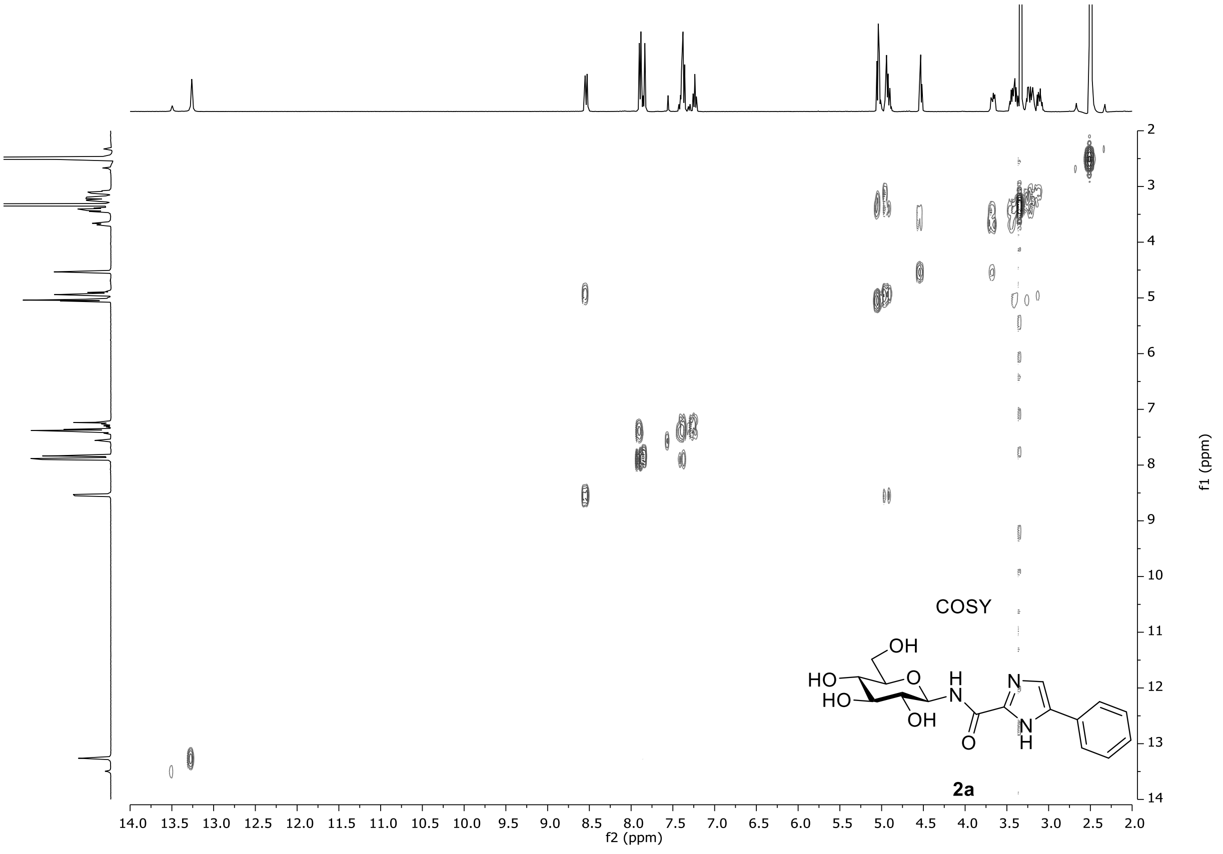
COSY
2a
f2 (ppm)
f1 (ppm)

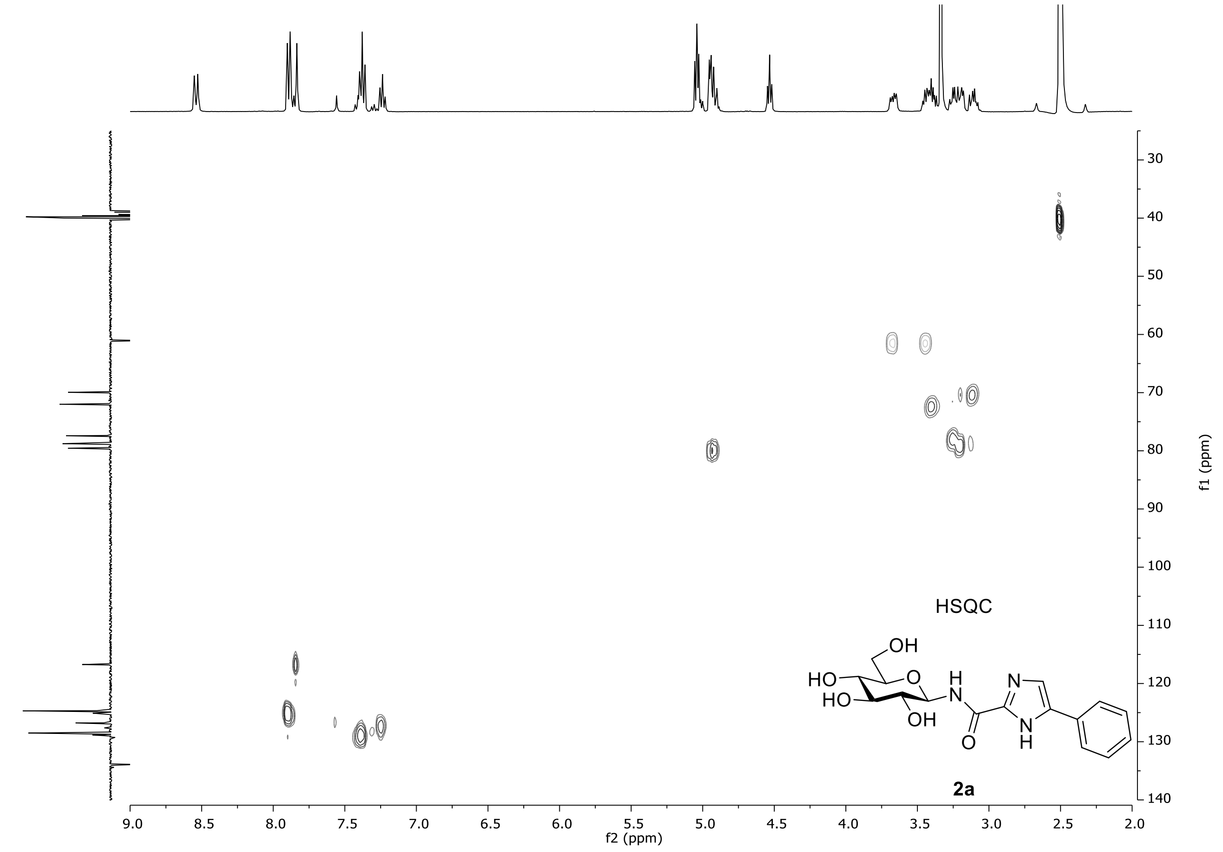
HSQC
2a
f2 (ppm)
f1 (ppm)

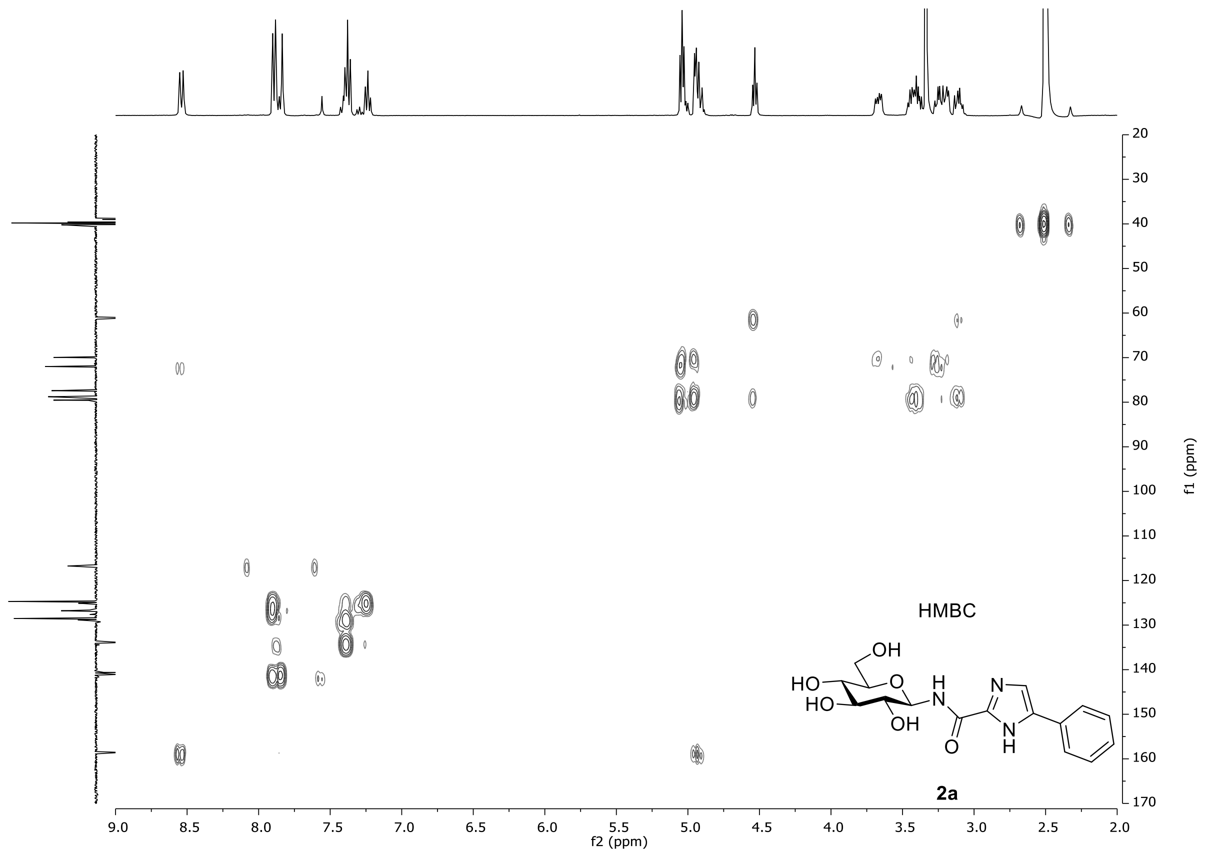
HMBC
2a
f2 (ppm)
f1 (ppm)

N-(β-D-Glucopyranosyl)-4(5)-(1-naphthyl)-1*H*-imidazole-2-carboxamide (2b)

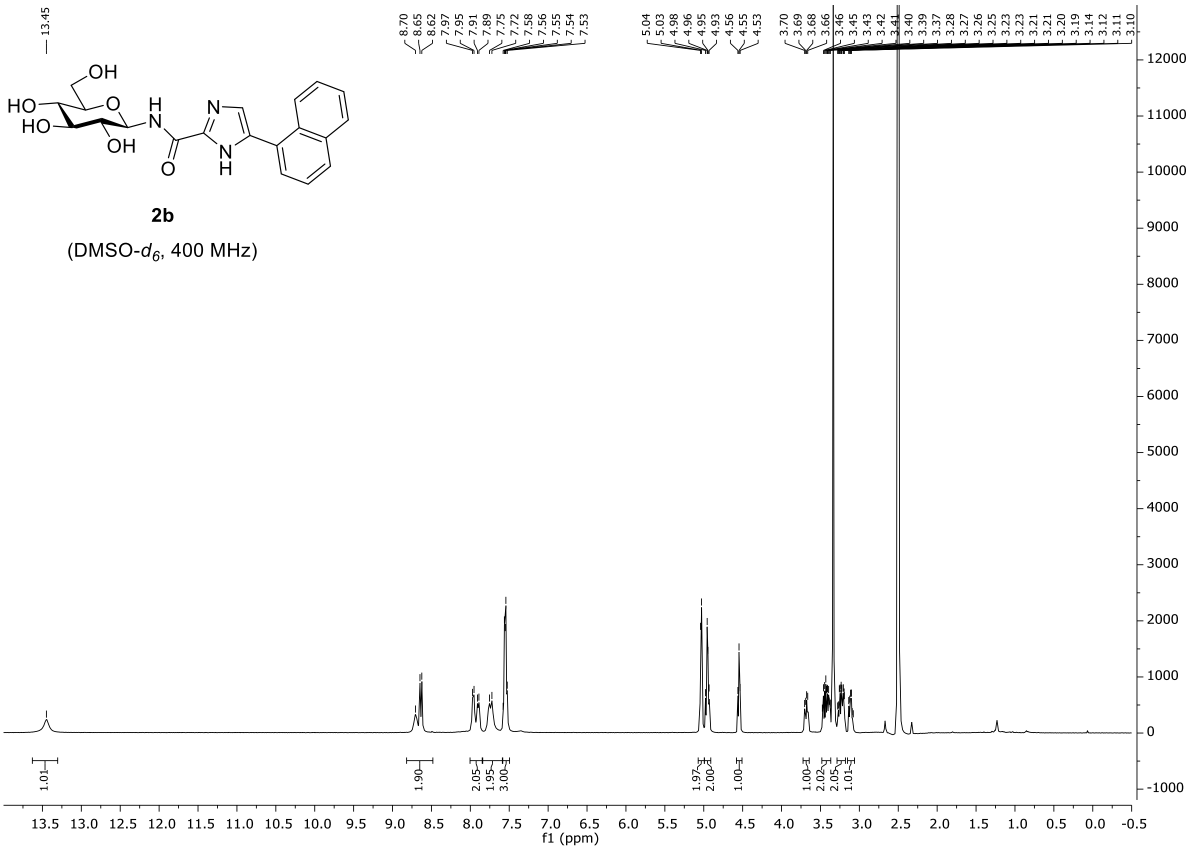

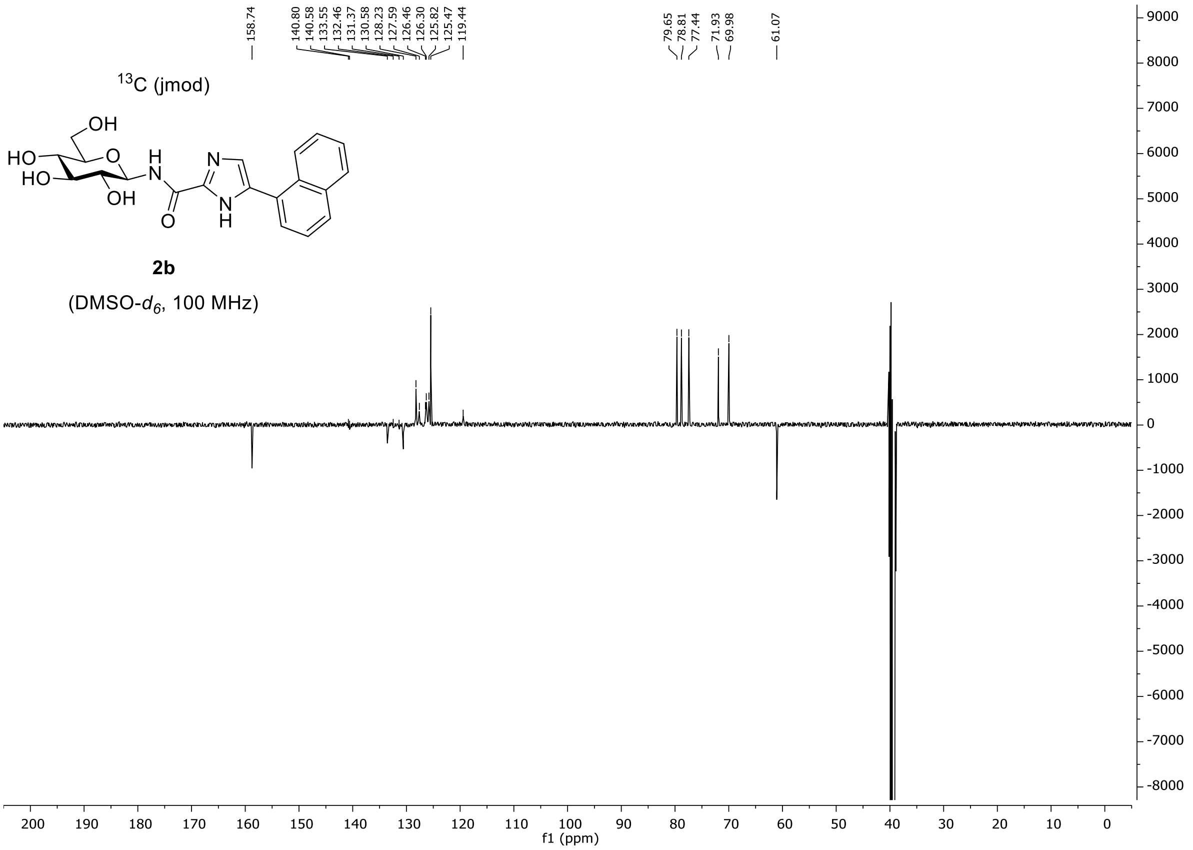

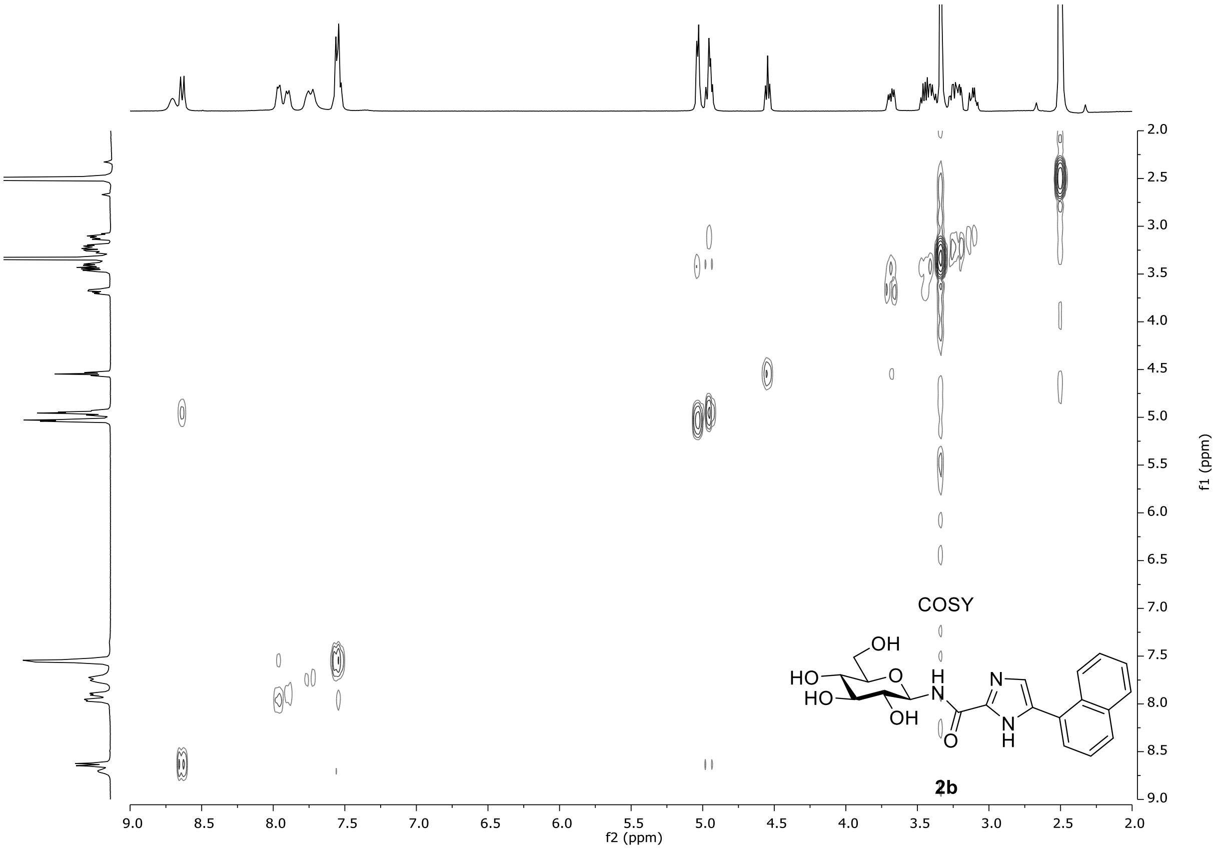
COSY
2b
f2 (ppm)
f1 (ppm)

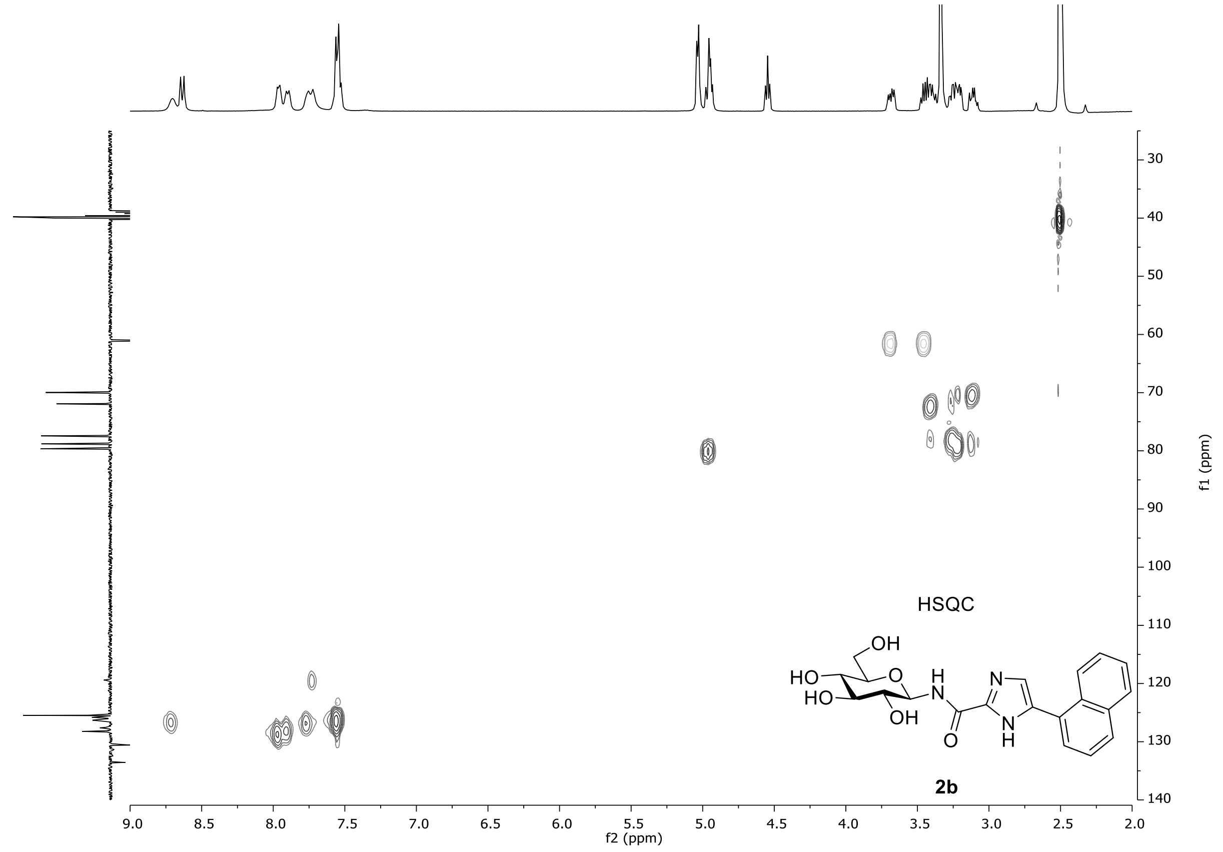
HSQC
2b
f2 (ppm)
f1 (ppm)

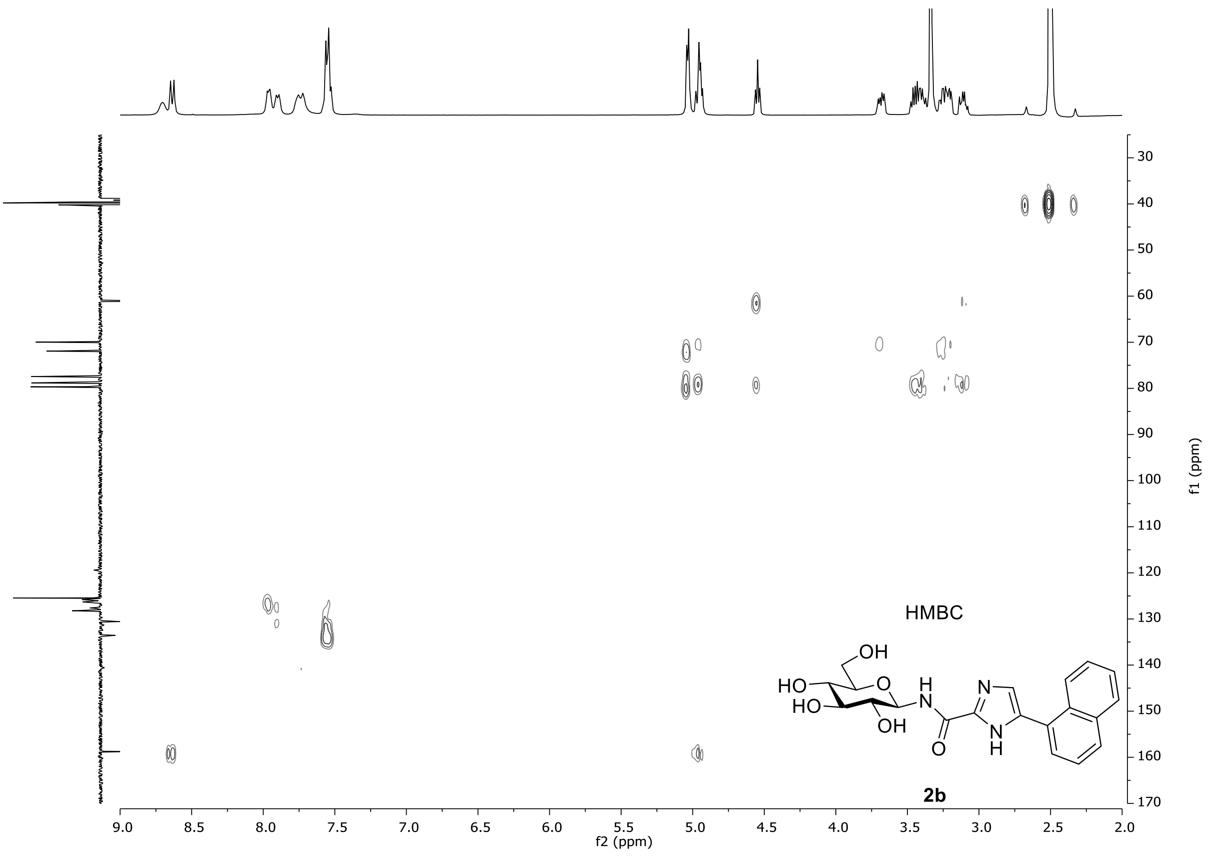

HMBC
2b
f2 (ppm)
f1 (ppm)

N-(β-D-Glucopyranosyl)-4(5)-(2-naphthyl)-1*H*-imidazole-2-carboxamide (2c)

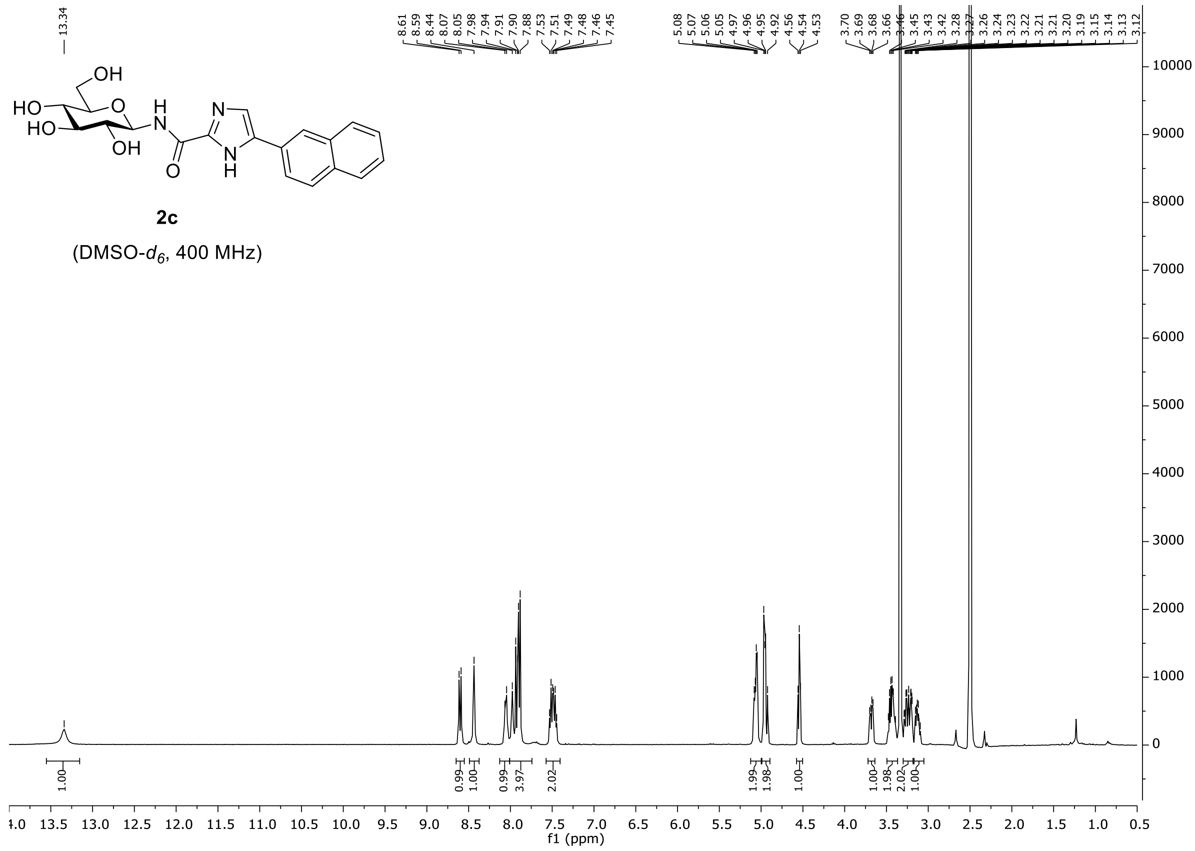

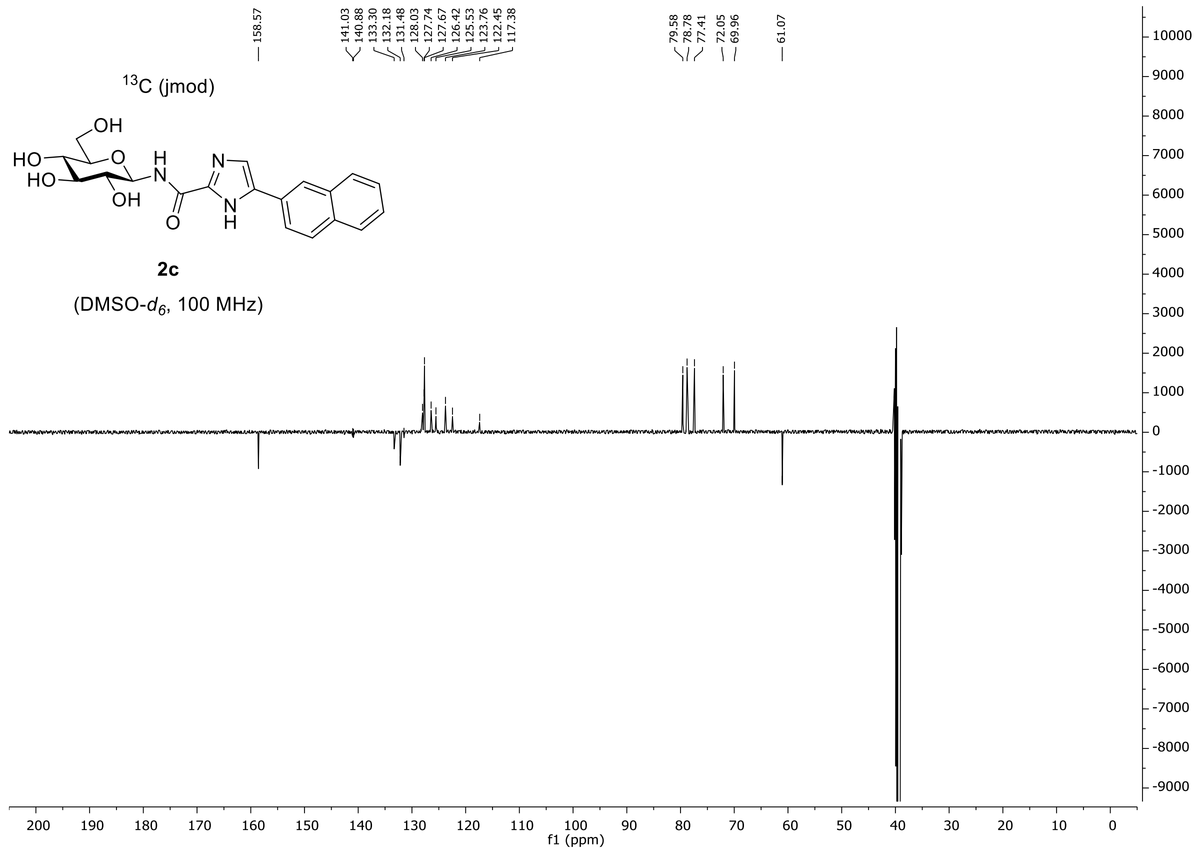

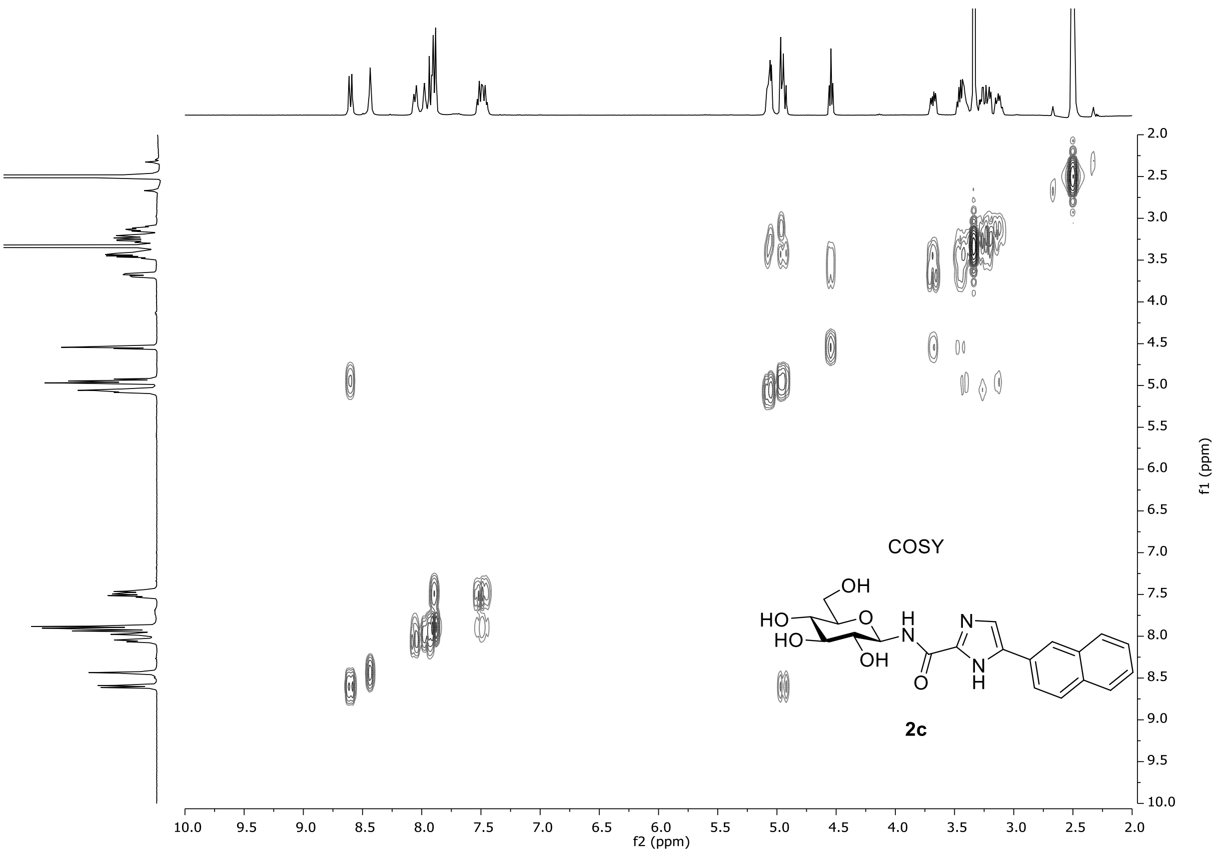
COSY
HO
HO
OH
OH
O
H
N
O
N
N
H
2c
f1 (ppm)
f2 (ppm)

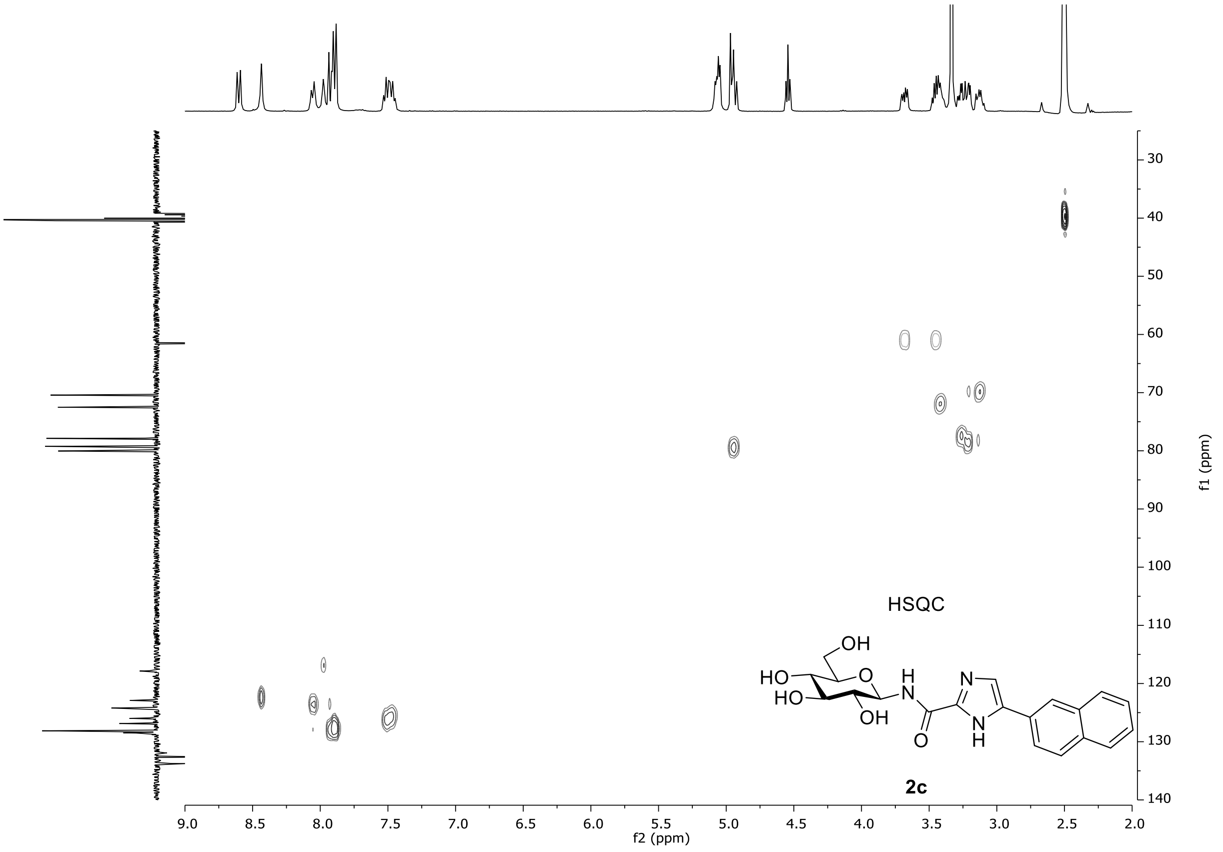
HSQC
HO
HO
OH
OH
O
H
N
O
N
N
H
2c
f1 (ppm)
f2 (ppm)

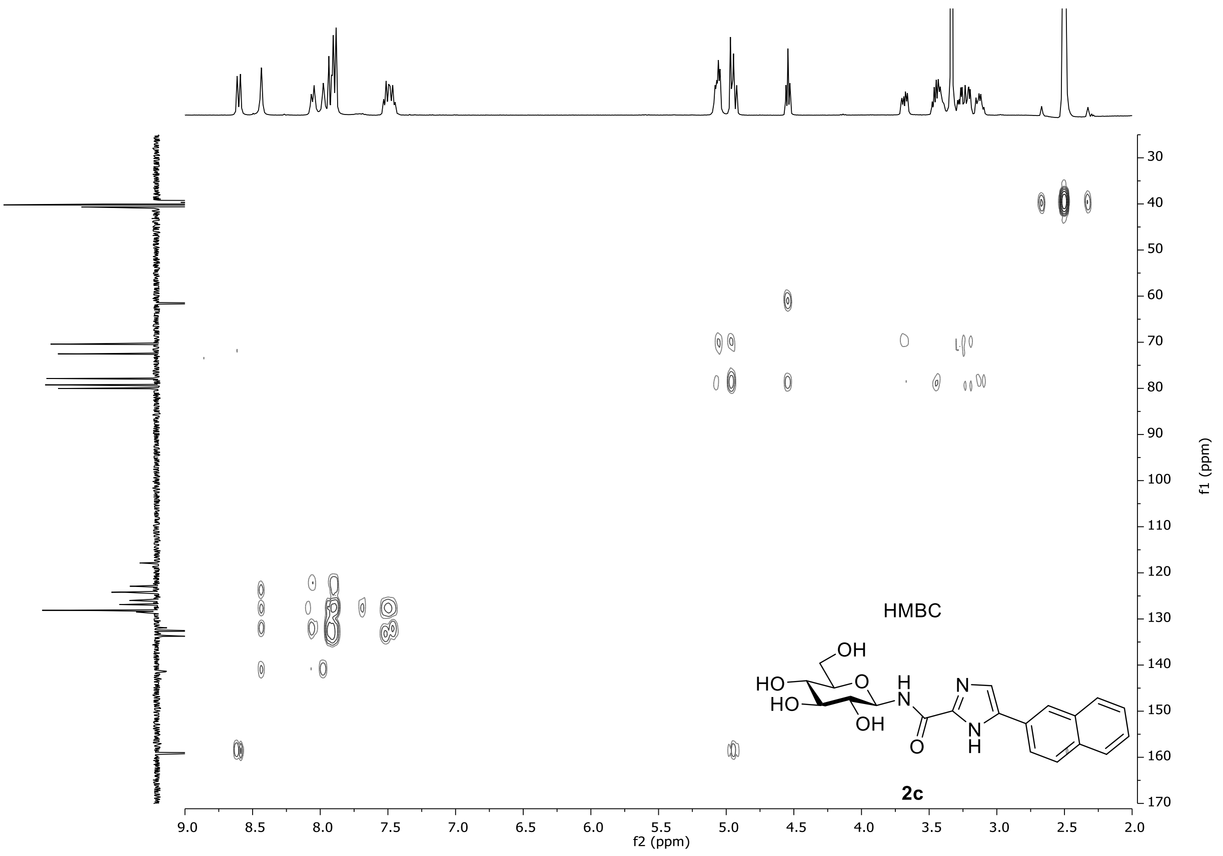
HMBC
2c
f2 (ppm)
f1 (ppm)
